# Anti-Cancer Activity of Novel Dihydrotestosterone-Derived Ring A-Condensed Pyrazoles on Androgen Non-Responsive Prostate Cancer Cell Lines

**DOI:** 10.3390/ijms20092170

**Published:** 2019-05-02

**Authors:** Gergő Mótyán, Mohana Krishna Gopisetty, Réka Eleonóra Kiss-Faludy, Ágnes Kulmány, István Zupkó, Éva Frank, Mónika Kiricsi

**Affiliations:** 1 Department of Organic Chemistry, University of Szeged, Dóm tér 8, H-6720 Szeged, Hungary; motyan@chem.u-szeged.hu (G.M.); elara.elinor.95@gmail.com (R.E.K.-F.); 2 Department of Biochemistry and Molecular Biology, University of Szeged, Közép fasor 52., H-6726 Szeged, Hungary; gmohanakrishna@bio.u-szeged.hu; 3 Department of Pharmacodynamics and Biopharmacy, University of Szeged, Eötvös u. 6, H-6720 Szeged, Hungary; kulmany.agnes@gmail.com (Á.K.); zupko@pharm.u-szeged.hu (I.Z.)

**Keywords:** dihydrotestosterone, heterocyclization, pyrazoles, regioselectivity, prostate cancer, anti-cancer effect, apoptosis

## Abstract

Regioselective synthesis of novel ring A-fused arylpyrazoles of dihydrotestosterone (DHT) was carried out in two steps under facile reaction conditions. Aldol condensation of DHT with acetaldehyde afforded a 2-ethylidene derivative regio- and stereo-selectively, which was reacted with different arylhydrazines in the presence of iodine via microwave-assisted oxidative cyclization reactions. The 17-keto analogs of steroidal pyrazoles were also synthesized by simple oxidation in order to enlarge the compound library available for pharmacological studies and to obtain structure–activity relationship. The antiproliferative activities of the structurally related heteroaromatic compounds were tested in vitro on human cervical and breast adenocarcinoma cell lines (HeLa, MCF-7 and MDA-MB-231) and on two androgen-independent malignant prostate carcinoma cell lines (PC-3 and DU 145). Based on primary cytotoxicity screens and IC_50_ assessment, a structure-function relationship was identified, as derivatives carrying a hydroxyl group on C-17 exhibit stronger activity compared to the 17-one counterparts. Cancer cell selectivity of the derivatives was also determined using non-cancerous MRC-5 cells. Furthermore, the proapoptotic effects of some selected derivatives were verified on androgen therapy refractive p53-deficient PC-3 cells. The present study concludes that novel DHT-derived arylpyrazoles exert cancer cell specific antiproliferative activity and activate apoptosis in PC-3 cells.

## 1. Introduction

Endogenous androgenic-anabolic steroids (AAS), such as testosterone (T) and its more active derivative, dihydrotestosterone (DHT) play a key role in the development and progression of hormone-dependent prostate cancer in men [1] and may also exert a direct activating or inhibitory effect on the growth of certain female breast cancers primarily by influencing the androgen receptor (AR) signaling pathway [2,3].

Since AR signaling is a substantial factor in malignant cell proliferation within the prostate tissue [4,5], the first-line therapy often involves hormone deprivation either via surgical manipulation of the endocrine system or by the exogenous administration of drugs. These procedures will ultimately reduce the levels of sex hormones by inhibiting their production, or block the action of the endogenous ligand by binding to the hormone receptor, thereby preventing its activation [6]. Unfortunately, prostate cancer cells are able to develop different resistance mechanisms over time to maintain AR signaling and to circumvent the reduced synthesis and the blocked action of androgens [7]. As a consequence, a castration resistant stage of the disease evolves, for which there is no life-prolonging therapy to date. AR-signaling plays a crucial role in AR+ breast cancers as well, however, in such cases coordination of the cellular features and actions is quite complex and depends on the presence or absence of other signaling mechanisms [8]. For instance, early studies have demonstrated proliferation promoting as well as antiproliferative effects of androgens on cell activity, depending largely on the specific breast cancer subtype [9].

All of the therapies employ drugs—alone or in combination—mostly for aiming at the hormone-signaling pathway. However occasionally, hormone receptor-independent mechanisms are targeted as well, those in particular, which were compromised during cancer development and progression to modulate cell cycle regulation and support cellular proliferative properties. For example, experimental results revealed that some heterocyclic steroidal derivatives can influence microtubule dynamics [10] and apoptosis [11] in a hormone receptor-independent manner by inhibiting tubulin polymerization and upregulating key factors involved in triggering the intrinsic apoptotic pathway [12,13,14]. The main advantage of aiming these cellular features is to offer a solution for the therapy of hormone-resistant cancers. Yet when steroidal compounds of such mode of action are applied, the primary hormonal effect is undesirable, therefore, structural modifications are needed in order to reduce or eliminate the ability of the molecule to interact with the androgen and estrogen receptors. Based on extensive structure–activity relationship studies and our knowledge about the exact structure and ligand binding site of hormone receptor proteins [15,16], new derivatives lacking the structural features necessary for receptor binding can be properly designed.

**Figure 1:**
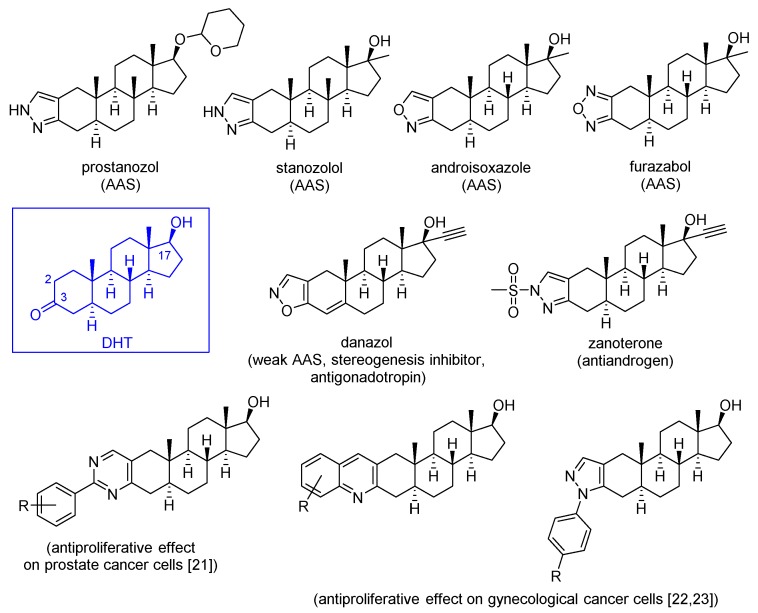
Ring-A condensed heterocyclic derivatives of dihydrotestosterone (DHT) with marked biological activities.

Amongst semi-synthetic androgens, relatively few DHT derivatives with a ring A-fused heterocycle have been synthesized so far (Figure 1). The presence of the 17β-OH group of DHT is essential, while the 3-keto group is favorable for binding to the AR [17]. Thus, the hormonal effect can be reduced or eliminated by a significant structural change at these or nearby positions. Moreover, according to structure–activity relationship studies, steric rather than electronic factors are decisive for the structural requirements at C-2 and/or C-3 in androgens [18]. It is also important to emphasize that DHT and its derivatives are not aromatizable androgens [19], hence, they are not able to exert estrogen-like effects in the body by binding to estrogen receptors. Earlier, structural modifications of DHT were directed to achieve a separation of androgenic and anabolic effects, and therefore some derivatives, such as prostanozol, stanozolol, androisoxazole, furazabol and danazol bearing a 2,3-fused heteroring have been synthesized (Figure 1). These compounds exhibit a better anabolic-to-androgenic ratio than the natural compound, i.e., the androgenic activity is reduced, while the anabolic action is retained or enhanced by the incorporation of the heterocyclic moiety. Nevertheless, it has to be noted that several of these derivatives possess unpredictable endocrinological activities [15]. Interestingly, antiandrogenic effect without any other hormonal activities prevails for zanoterone, a compound with a substitution at the pyrazole-*N* [20]. In spite of the obvious fact that the incorporation of different heterorings to 2,3 position of DHT and to its analogs may significantly alter the bioactivity of the parent molecule, very few examples are to be found in the literature for such kind of transformations. In this respect, we previously demonstrated that different heteroaryl-fused derivatives of DHT exert significant antiproliferative effect in vitro on human cancer cell lines of diverse origins [21,22,23].

In view of the potential biological significance of heterocyclic DHT analogs, and the well-known antiproliferative potential of numerous steroids containing pyrazoline or pyrazole scaffolds [24,25,26,27], we now describe a facile, one-pot heterocyclization/oxidation sequence of a DHT-derived steroidal enone with different arylhydrazines in the presence of iodine under microwave (MW) conditions. The stepwise sequence, i.e., the cyclocondensation of the unsaturated ketone to pyrazolines, and the subsequent oxidation to pyrazoles was also investigated. Finally, the 17-keto derivatives were synthesized as well in order to enlarge the compound library available for pharmacological studies. All of the synthesized compounds were primarily screened in vitro for their antiproliferative activity on five different cancer cell lines, namely DU 145, PC-3, HeLa, MCF-7 and MDA-MB-231 and on non-cancerous MRC-5 fibroblasts. Our results indicate that several compounds exhibit cancer cell specific and dose dependent inhibition of cell growth. Based on these data, five compounds featuring outstanding cancer cell specificity were selected for further experiments i.e., to determine IC_50_ values. Annexin V–propidium iodide staining and quantitative-real time PCR were performed to examine in details the proapoptotic activity of selected ring A-fused arylpyrazole derivatives of DHT on androgen therapy refractive p53 deficient PC-3 cells.

## 2. Results and Discussion

### 2.1. Synthetic Studies

For the synthesis of steroidal ring A-fused pyrazole derivatives, DHT was first subjected to aldol condensation with an excess of acetaldehyde in alkaline ethanol at low temperature in order to obtain the starting α,β-enone suitable for heterocyclization. Although the similar aldol-type Claisen–Schmidt reaction of cyclic ketones with different arylaldehydes is frequently applied for the efficient synthesis of arylidene ketones [28,29], the use of acetaldehyde is fairly unusual due to its greater reactivity and ability to undergo undesirable self-condensation [30]. Nevertheless, the transformation led to the regioselective formation of 2-ethylidene-DHT (**1**) in good yield (70%) after purification by column chromatography (Table 1). The expected (E)-configuration along the double bond was evidenced by NOESY correlations between the 1β-H and 21-CH_3_ protons (Supplementary Material).

**Scheme 1:**
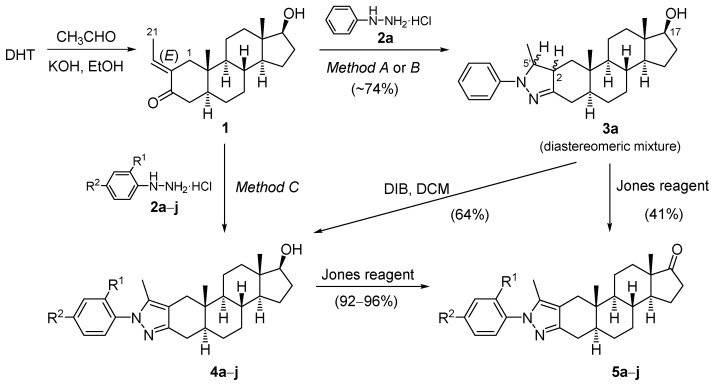
Synthesis of ring A-fused arylpyrazole derivatives of DHT. Reagents and conditions: Method A: p-toluenesulfonic acid (PTSA) (0.5 equiv.), EtOH, reflux, 78 °C, 1 h; Method B: PTSA (0.5 equiv.), EtOH, MW, 100 °C, 2 min; Method C: I_2_ (0.5 equiv.), EtOH, MW, 100 °C, 2 min.

Preliminary ring-closure experiments on **1** with phenylhydrazine hydrochloride **2a** in the presence of p-toluenesulfonic acid (PTSA) were first carried out both under conventional heating (Method A) and MW conditions (Method B), and a mixture of pyrazoline diastereomers of **3a** was obtained owing to the formation of two new chiral centers on C-2 and C-5′ (Scheme 1). The MW-assisted method greatly shortened the reaction time, but the yields of the desired products were only slightly better than in the thermally-induced version. The stereoisomers of **3a** could not be separated by column chromatography and spontaneous oxidation of the pyrazoline moiety to the corresponding pyrazole during purification was also observed. In view of the tendency of pyrazolines to undergo autoxidation, further experiments toward their stereoselective synthesis were not performed. Mild oxidation of the diastereomeric mixture of **3a** to a single heteroaromatic pyrazole (**4a**) was successfully achieved with diacetoxyiodobenzene (DIB) in dichloromethane (DCM) at room temperature, but the product was obtained only with a moderate yield (64%) after purification (Scheme 1). At the same time, complete oxidation of **3a** with the Jones reagent in acetone affected both the pyrazoline ring and the 17-OH group, which resulted in the corresponding heteroaromatic 17-keto derivative **5a** with a yield of 41%.

In order to avoid the multistep synthesis and to improve the yield of the desired pyrazole **4a**, a one-pot procedure, involving the I_2_-mediated oxidative cyclization [31] of **1** with different arylhydrazines (**2a**–**j**) has been developed under MW condition (Scheme 1, Method C). The improved practical protocol led to ring A-fused arylpyrazoles (**4a**–**j**) in excellent yields (81–89%) without the necessity of isolating the less stable pyrazoline intermediates (Table 1, entries 1–10). Subsequent oxidation of the 17β-hydroxy derivatives (**4a**–**j**) with the Jones reagent also made the 17-keto analogs (**5a**–**j**) readily available for pharmacological comparison.

**Figure 2:**
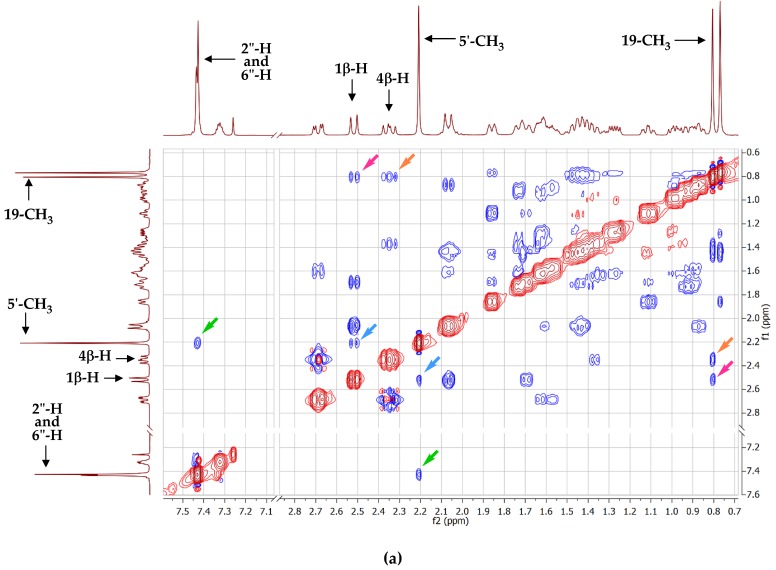

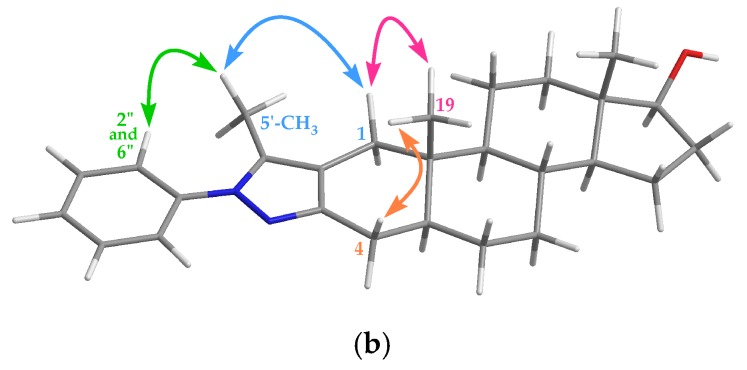
(**a**) Partial NOESY spectrum of compound **4a**; (**b**) NOESY correlations between protons observed for **4a**.

The structure of the synthesized compounds was confirmed by ^1^H and ^13^C NMR measurements, which indicated the presence of the characteristic signals of the aromatic ring from the arylhydrazine reagent and the tri-substituted pyrazole moiety formed by the ring-closure reaction (Supplementary Material). The acid-catalyzed heterocyclization of arylhydrazines with α,β-enones can occur either by 1,2- [32] or 1,4-addition [33] leading to two different regio-isomeric pyrazoles. Therefore, 2D NMR spectra were recorded for a representative compound (**4a**) in order to determine the exact structure. After identification of the related ^1^H and ^13^C signals with the help of HSQC (Heteronuclear Single Quantum Correlation) and HMBC (Heteronuclear Multiple Bond Correlation) spectra, the orientation of the pyrazole ring was determined on the basis of through-space (NOESY) correlations. Thus, spatial proximity was evidenced by cross-peaks between 2”-H–5′-CH_3_, 5′-CH_3_–1β-H, 1β-H–19-CH_3_ and 4β-H–19-CH_3_ supporting the exclusive formation of regio-isomer **4a** (Figure 2). Taking into account that the terminal nitrogen in arylhydrazines is more nucleophilic than the internal one [23], the reaction of **1** with **2a**–**j** is considered to proceed via hydrazone intermediates followed by intramolecular ring-closure. The NMR spectra recorded for the oxidized products (**5a–j**) also confirmed the expected structures (Supplementary Material). The only difference compared to the ^1^H spectra of compounds **4a–j** is the disappearance of the triplet signal of 17α-H at 3.65 ppm. At the same time, the signal of carbonyl-C could be observed at around 221 ppm in the ^13^C NMR spectra instead of the C-17 signal at 81.9 ppm characteristic for **4a–j**.

### 2.2. Pharmacological Studies

#### 2.2.1. Cell Type Specific Antiproliferative Action of DHT-Derived Arylpyrazoles

**Figure 3:**
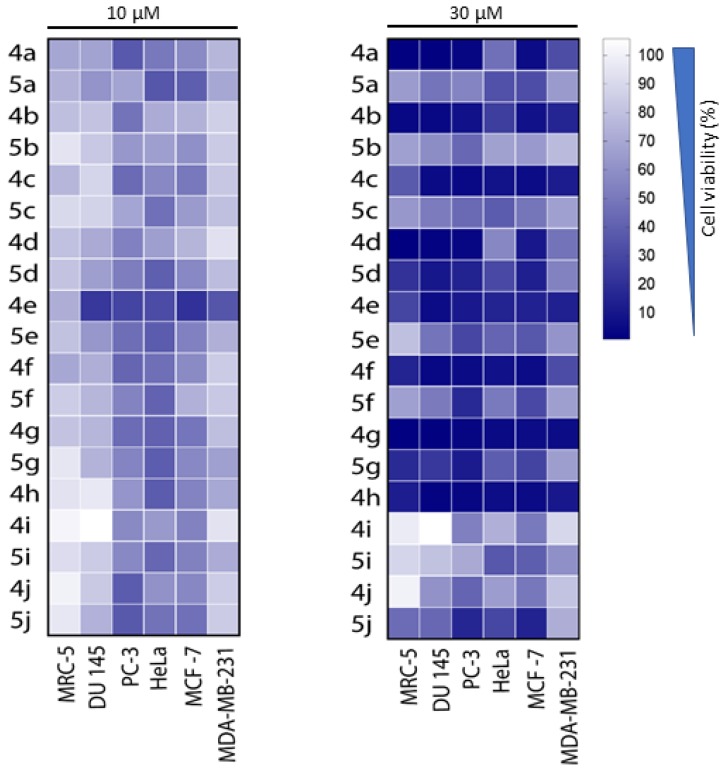
Primary antiproliferative action of the synthesized DHT derivatives on different cell lines.

To perform pharmacological studies, the synthesized DHT derivatives were dissolved in DMSO (dimethyl sulfoxide) in a final concentration of 5 mM. One compound (**5h**) was non-soluble in the applied solvent, thus was excluded from the pharmacological evaluations. Cancer cell specific growth inhibition exhibited by the ring-condensed pyrazole derivatives applied in 10 μM and in 30 μM concentrations was screened and the effect of each compound on cell viability was assessed. For this, PC-3 and DU 145 prostate cancer cells, MCF-7 and MDA-MB-231 breast cancer cells, HeLa cervical cancer cells and non-cancerous MRC-5 fibroblasts were used. Based on viability data a heat map (Figure 3) was constructed showing differential activities exhibited by the analogs depending on the tested cell type (represented as viability in %). All compounds showed concentration-dependent antiproliferative action as 30 µM concentrations were generally more effective than 10 µM. On the heat map representing viability results following treatments with DHT derivatives at 30 µM concentration a remarkable structure–function relationship can be recognized (Figure 3). Most of the derivatives of the series **4**, which carry a hydroxyl group on C-17 (**4a**–**j**) exhibited more potent cancer cell growth inhibition compared to the 17-one counterparts (**5a**–**j**), thus, the presence of the OH-group at C-17 seems to have a determining role in the biological activity of these pharmacological candidates. Among all examined compounds, **4e** showed the utmost specificity in cancer cell toxicity, which manifested already at 10 µM concentration. In addition to **4e**, the primary screen also resulted in several positive hits regarding prostate cancer or in most cases rather PC-3 cell specificity. Among them especially **4a**, **4b**, **4c**, **4g**, **4i**, **4j**, **5f**, **5g** and **5j** featured significantly higher antiproliferative potency against PC-3 prostate cancer cells compared to MRC-5 non-cancerous fibroblasts at 10 µM and **4i**, **4j**, **5b**, **5e**, **5f** and **5j** at 30 µM concentration.

Therefore, as a hit validation, the IC_50_ values of five selected compounds (**4a**, **4e**, **4g**, **4j** and **5j**) were determined on all the applied cancer cell lines and on non-cancerous fibroblasts (Figure S1a and S1b). The IC_50_ values of the five compounds were generally markedly lower on cancer cell lines than on non-cancerous MRC-5 cells (Table 2). The selective toxic effects of these compounds were especially prominent on PC-3 cells, marked by the lowest IC_50_ values.

Comparing the performance of these compounds with clinically applied chemotherapeutic drugs like cisplatin—based on the IC_50_ values of cisplatin (230.9 ± 1.37 µM on PC-3, 126.8 ± 1.18 µM on DU 145 and 237.3 ± 1.16 µM on MRC-5 cells) assessed previously [21]—we observe that the tested DHT derivatives all performed better than cisplatin on the examined prostate cancer cell lines (Table 2). These findings are highly relevant, especially related to PC-3 cells, since these cells are considered to have a high metastatic potential, lack functional p53 and are relatively unresponsive to androgens. Therefore, our further experiments were conducted only on PC-3 prostate cancer cells with two selected compounds **4e** and **4j**.

#### 2.2.2. Apoptosis-Inducing Properties of Selected DHT Derivatives on PC-3 Prostate Cancer Cells

The counter-balance of cell proliferation and death is necessary to maintain normal cell turnover, hence they are programmed and regulated precisely. Upregulation of proliferation and/or downregulation of cell death mechanisms are generally associated with cancer development [34]. Among various programmed cell death mechanisms, deregulation of apoptosis drives the growth of a large number of cancers including prostate cancer. It has already been shown that PC-3 cells have unique cellular features to cope with various stress situations partly due to the inability to express the tumor suppressor p53. This protein normally serves as a multifunctional platform to overcome cellular impairments such as DNA damage, oxidative stress, activation of oncogenes, hypoxia and heat shock [35]. Activation of p53 leads to enhanced transcription of p53 responsive genes, which mediate cell cycle arrest and initiate apoptosis [36].

**Figure 4:**
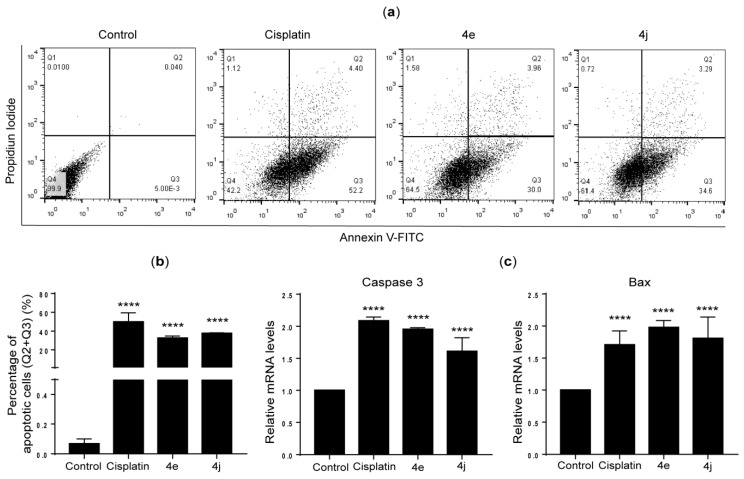
Selected DHT derivatives induce apoptosis in PC-3 prostate cancer cells. (**a**) Dot plots of annexin V/PI-based apoptosis detection and (**b**) quantification of apoptotic cells. (**c**) Bar graph of relative mRNA levels of caspase 3 and Bax in PC-3 cells. Data are presented as the mean ± standard deviation (SD). **** p < 0.0001 (Fisher’s LSD test).

A structurally related and similarly effective cell growth inhibiting androstane derivative elicited apoptosis as evidenced by increased hypodiploid (subG1) cell population on MDA-MB-231 breast cancer cells [23]. Therefore, we aimed to find out whether DHT-derived pyrazoles are capable of inducing apoptosis and/or necrosis in p53 deficient PC-3 cells and whether these mechanisms lay behind the observed anti-cancer effects of the selected derivatives. Along this line, PC-3 prostate cancer cells were treated with either **4e**, **4j** or for comparison, with cisplatin. We performed annexin V/PI apoptosis detection assay and the results (Figure 4a) indicate that **4e** and **4j** treatments induced significant apoptosis marked by the high percentage of annexin V-positive cells (Q2 + Q3) 33.96% and 37.89%, respectively, whereas less than 1% annexin V-positive cells were detected in the untreated control (Figure 4b). Cisplatin treatment also led to a prominent apoptosis in our experimental set-up. The percentage of only PI-positive cells (Q1) was not changed following treatments, therefore DHT derivatives do not seem to induce necrosis directly. To further verify apoptosis activation in PC-3 cells, we performed quantitative real-time PCR and examined the transcriptional activation of apoptotic markers caspase 3 and Bax. Our results (Figure 4c) show significantly increased caspase 3 and Bax mRNA expression levels, supporting the activation of apoptotic cell death pathway in **4e** and **4j** as well as in cisplatin treated PC-3 cells compared to untreated control. These results suggest that both derivatives (**4e** and **4j**) induce apoptosis in PC-3 prostate cancer cells.

Many cancer cells are capable of removing the tumor suppressing effect of p53, in some cases by point mutations in the DNA binding domain or via deletion of p53 chromosomal locus from the genome. In PC-3 cells the single copy of the p53 gene has a deletion which causes a frame-shift and a new in-frame stop codon leading to the lack of functional p53 expression [37]. This p53 inactivation renders the drug-induced apoptosis-based elimination of cancer cells rather difficult, manifesting significant chemoresistance towards anticancer drugs, and establishing a strong correlation between p53 status and therapeutic outcome [38,39]. However, it has been shown that other mechanisms such as disruption of mitochondrial function, endoplasmic reticulum stress or autophagy can also induce apoptosis in cancer cells by stimulating p53-independent events [40,41,42,43]. Given that nearly 50% of all human cancers have been characterized by impaired p53 function, p53-independent pathways could be exploited in chemotherapy drug design and development. Since we observed prominent antiproliferative actions and massive induction of apoptotic cell death following **4e** and **4j** treatments, we believe that PC-3 cells can be effectively eliminated by selected DHT derivatives. The fact that p53-independent apoptosis can be triggered by these compounds, enhances their potential as cancer therapy candidates.

## 3. Materials and Methods

### 3.1. Chemistry

#### 3.1.1. General Information 

Reagents and materials were purchased from commercial suppliers (TCI, Tokyo, Japan; Alfa Aesar, Haverhill, MA, USA and Sigma-Aldrich Corporation, St. Louis, MO, USA). All solvents were dried and purified according to standard procedures. MW-assisted reactions were carried out with a CEM Discover SP instrument (CEM Corporation, Matthews, NC, USA) using a maximum power of 200 W with dynamic control program. Thin layer chromatography (TLC) was carried out on Kieselgel-G (Si 254 F, Merck KGaA, Darmstadt, Germany) plates (0.25 mm thick). The spots were detected by spraying with phosphomolybdic acid (5%) in aqueous phosphoric acid (50%) or visualized with UV light 254 nm. The products were purified with preparative column chromatography on Merck silica gel 60, 40–63 μm (Merck KGaA). Melting points (Mps) were measured on an SRS Optimelt digital device (Stanford Research Systems Inc, Sunnyvale, CA, USA). Elementary analysis data were obtained with a PerkinElmer CHN analyzer model 2400 (PerkinElmer Inc, Waltham, MA, USA). NMR spectra were recorded at 298 K with a Bruker DRX 500 instrument (Bruker, Billerica, MA, USA). Chemical shifts are reported in ppm (δ scale) and coupling constants (J) in Hz. The ^1^H resonance signals are indicated as a singlet (s), a doublet (d), a double doublet (dd), a triplet (t), a triplet of doublets (td) or a multiplet (m). ^13^C NMR spectra are ^1^H-decoupled. The J-MOD pulse sequence was applied to determine multiplicities. An HPLC/MSD system was used for automated flow injection analyses. Components of the system: An Agilent 1100 micro vacuum degasser (Agilent Technologies, Santa Clara, CA, USA) a quaternary pump, a micro-well plate autoinjector and a 1946A MSD equipped with an electrospray ion source (ESI) operated in positive ion mode. ESI parameters: Nebulizing gas N_2_, at 35 psi; drying gas N_2_, at 350 °C and 12 L/min; capillary voltage 3000 V; fragmentor voltage 70 V. The MSD was operated in scan mode with a mass range of m/z 60−620. Samples (0.2 μL) were injected directly into the solvent flow (0.3 mL/min) of CH_3_CN/H_2_O 70:30 (*v/v*) supplemented with 0.1% formic acid with automated needle wash. Agilent LC/MSD Chemstation software (C.01.08, Agilent Technologies Inc) was used to control the system.

#### 3.1.2. Synthesis of 2-Ethylidene-DHT (1)

DHT (2.9 g, 10 mmol) and KOH (561 mg, 10 mmol) were dissolved in absolute EtOH (40 mL), and the homogenous solution was cooled to approximately −10 °C in an ice bath containing sodium chloride. (Cooling was applied during the whole procedure.) Afterward, acetaldehyde (1.01 mL, 18 mmol) was added to the mixture and it was stirred at the given temperature. After 1.5 h, a further amount of acetaldehyde (0.25 mL, 4.5 mmol) was added to the reaction mixture and it was stirred for 2 h. After completion of the reaction, the pH was adjusted to around 7 with 1 M H_2_SO_4_ solution in EtOH, and the mixture was evaporated in vacuo. The residual oil was dissolved in EtOAc and washed with water (2 x 10 mL), then the combined organic layers were dried over anhydrous Na_2_SO_4_ and evaporated in vacuo. The crude product was purified by column chromatography with EtOAc/CH_2_Cl_2_ = 2:98. Yield: 2.2 g (white solid); Mp 137–141 °C; Anal. Calcd. for C_21_H_32_O_2_ (316.49): C, 79.70; H, 10.19. Found: C, 79.84; H, 10.25. ^1^H NMR (CDCl_3_, 500 MHz): δ 0.76 (s, 3H, 18-H_3_), 0.81 (s, 3H, 19-H_3_), 0.83–1.01 (overlapping m, 3H), 1.11 (m, 1H), 1.18–1.31 (overlapping m, 2H), 1.35–1.47 (overlapping m, 4H), 1.58–1.70 (overlapping m, 5H), 1.72 (dd, 3H, *J* = 7.2 Hz, *J* = 2.2 Hz, 21-H_3_), 1.84 (d, 1H, *J* = 15.2 Hz, 1α-H), 1.87 (m, 1H), 2.06 (m, 1H, 16α-H), 2.14 (dd, 1H, *J* = 18.5 Hz, *J* = 13.2 Hz, 4β-H), 2.33 (dd, 1H, *J* = 18.5 Hz, *J* = 5.2 Hz, 4α-H), 2.76 (d, 1H, *J* = 15.2 Hz, 1β-H), 3.65 (t, 1H, *J* = 8.6 Hz, 17α-H), 6.74 (m, 1H, 20-H); ^13^C NMR (CDCl_3_, 125 MHz): δ 11.0 (C-18), 11.8 (C-19), 13.6 (C-21), 20.9 (C-11), 23.4 (C-15), 28.4 (C-6), 30.5 (C-16), 31.0 (C-7), 35.4 (C-8), 35.6 (C-10), 36.6 (C-12), 39.7 (C-1), 42.5 (C-5), 42.7 (C-4), 42.8 (C-13), 50.9 (C-14), 53.7 (C-9), 81.8 (C-17), 135.7 (C-20), 136.1 (C-2), 200.8 (C-3); ESI-MS 317 [M+H]^+^.

#### 3.1.3. General Procedure for the One-Pot Synthesis of Ring A-Condensed Pyrazoles 4a–j under MW Irradiation (Method C)

To a solution of **1** (187 mg, 0.60 mmol) in absolute EtOH (5 mL), I_2_ (152 mg, 0.6 mmol) and (substituted) phenylhydrazine hydrochloride (**2a–j**, 1.20 mmol) were added and the mixture was irradiated in a closed vessel at 100 °C for 2 min. After completion of the reaction, the mixture was poured into saturated aqueous solution of Na_2_S_2_O_3_ (10 mL) and extracted with CH_2_Cl_2_ (3× 10 mL). The combined organic layers were dried over anhydrous Na_2_SO_4_ and concentrated in vacuo. The crude product was purified by column chromatography with EtOAc/CH_2_Cl_2_ = 5:95.

17β-Hydroxy-1′-phenyl-5′-methylpyrazolo[3′,4′:3,2]-5α-androstane (**4a**)

According to the general procedure, phenylhydrazine hydrochloride (**2a**, 174 mg) was used. Yield: 206 mg (85%, white solid); Mp 241–245 °C; Anal. Calcd. for C_27_H_36_N_2_O (404.60): C, 80.15; H, 8.97. Found: C, 80.02; H, 8.90. ^1^H NMR (CDCl_3_, 500 MHz): δ 0.77 (s, 3H, 18-H_3_), 0.81 (s, 3H, 19-H_3_), 0.84–1.02 (overlapping m, 3H, 9α-H, 7α-H and 14α-H), 1.11 (td, 1H, *J* = 12.7, *J* = 3.7 Hz, 12α-H), 1.27 (m, 1H, 15β-H), 1.33–1.51 (overlapping m, 4H, 6β-H, 8β-H, 16β-H and 11β-H), 1.53–1.66 (overlapping m, 3H, 5α-H, 15α-H and 6α-H), 1.67–1.75 (overlapping m, 2H, 11α-H and 7β-H), 1.86 (m, 1H, 12β-H), 2.05 (d + m, 2H, *J* = 15.0 Hz, 1α-H and 16α-H), 2.21 (s, 3H, 5′-CH_3_), 2.34 (dd, 1H, *J* = 16.8 Hz, *J* = 11.9 Hz, 4β-H), 2.52 (d, 1H, *J* = 15.0 Hz, 1β-H), 2.67 (dd, 1H, *J* = 16.8 Hz, *J* = 5.1 Hz, 4α-H), 3.65 (t, 1H, *J* = 8.6 Hz, 17α-H), 7.32 (m, 1H, 4″-H), 7.43 (d, 4H, *J* = 4.4 Hz, 2″-H, 3″-H, 5″-H and 6″-H); ^13^C NMR (CDCl_3_, 125 MHz): δ 10.9 (5′-CH_3_), 11.0 (C-18), 11.8 (C-19), 20.8 (C-11), 23.4 (C-15), 27.4 (C-4), 29.2 (C-6), 30.5 (C-16), 31.3 (C-7), 34.8 (C-1), 35.8 (C-8), 36.2 (C-10), 36.7 (C-12), 42.4 (C-5), 42.8 (C-13), 50.9 (C-14), 54.0 (C-9), 81.9 (C-17), 115.1 (C-2), 124.5 (2C, C-2″ and C-6″), 127.1 (C-4″), 129.0 (2C, C-3″ and C-5″), 135.4 (C-5′), 139.6 (C-1″), 148.4 (C-3); ESI-MS 405 [M+H]^+^.

17β-Hydroxy-1′-(2″-tolyl)-5′-methylpyrazolo[3′,4′:3,2]-5α-androstane (**4b**)

According to the general procedure, 2-tolylhydrazine hydrochloride (**2b**, 190 mg) was used. Yield: 221 mg (88%, white solid); Mp 225–227 °C; Anal. Calcd. for C_28_H_38_N_2_O (418.63): C, 80.34; H, 9.15. Found: C, 80.27; H, 9.22. ^1^H NMR (CDCl_3_, 500 MHz): δ 0.77 (s, 3H, 18-H_3_), 0.81 (s, 3H, 19-H_3_), 0.85–1.01 (overlapping m, 3H, 9α-H, 7α-H and 14α-H), 1.11 (td, 1H, *J* = 13.0, *J* = 3.7 Hz, 12α-H), 1.27 (m, 1H, 15β-H), 1.35–1.51 (overlapping m, 4H, 6β-H, 8β-H, 16β-H and 11β-H), 1.56–1.65 (overlapping m, 3H, 5α-H, 15α-H and 6α-H), 1.67–1.76 (overlapping m, 2H, 11α-H and 7β-H), 1.86 (m, 1H, 12β-H), 1.95 (s, 3H), 2.04 (s, 3H), 2.08 (d + m, 2H, *J* = 15.2 Hz, 1α-H and 16α-H), 2.21 (s, 3H, 5′-CH_3_), 2.34 (dd, 1H, *J* = 16.2 Hz, *J* = 12.4 Hz, 4β-H), 2.52 (d, 1H, *J* = 15.2 Hz, 1β-H), 2.67 (dd, 1H, *J* = 16.2 Hz, *J* = 4.5 Hz, 4α-H), 3.65 (t, 1H, *J* = 7.8 Hz, 17α-H), 7.19–7.33 (overlapping m, 4H, 3″-H, 4″-H, 5″-H and 6″-H); ^13^C NMR (CDCl_3_, 125 MHz): δ 9.7 (5′-CH_3_), 11.1 (C-18), 11.8 (C-19), 17.3 (2″-CH_3_), 20.8 (C-11), 23.4 (C-15), 27.5 (C-4), 29.3 (C-6), 30.6 (C-16), 31.4 (C-7), 34.8 (C-1), 35.8 (C-8), 36.3 (C-10), 36.7 (C-12), 42.5 (C-5), 42.8 (C-13), 51.0 (C-14), 54.2 (C-9), 81.9 (C-17), 113.1 (C-2), 126.3 (CH), 128.0 (CH), 128.8 (CH), 130.8 (CH), 136.1, 136.2 and 138.6 (C-5′, C-1″ and C-2″), 147.8 (C-3); ESI-MS 419 [M+H]^+^.

17β-Hydroxy-1′-(4″-tolyl)-5′-methylpyrazolo[3′,4′:3,2]-5α-androstane (**4c**)

According to the general procedure, 4-tolylhydrazine hydrochloride (**2c**, 190 mg) was used. Yield: 206 mg (82%, white solid); Mp 247–248 °C; Anal. Calcd. for C_28_H_38_N_2_O (418.63): C, 80.34; H, 9.15. Found: C, 80.45; H, 9.24. ^1^H NMR (CDCl_3_, 500 MHz): δ 0.77 (s, 3H, 18-H_3_), 0.81 (s, 3H, 19-H_3_), 0.83–1.01 (overlapping m, 3H, 9α-H, 7α-H and 14α-H), 1.11 (td, 1H, *J* = 12.7, *J* = 3.5 Hz, 12α-H), 1.28 (m, 1H, 15β-H), 1.34–1.50 (overlapping m, 4H, 6β-H, 8β-H, 16β-H and 11β-H), 1.54–1.66 (overlapping m, 3H, 5α-H, 15α-H and 6α-H), 1.67–1.76 (overlapping m, 2H, 11α-H and 7β-H), 1.86 (m, 1H, 12β-H), 2.06 (d + m, 2H, *J* = 15.0 Hz, 1α-H and 16α-H), 2.18 (s, 3H, 5′-CH_3_), 2.34 (dd, 1H, *J* = 16.8 Hz, *J* = 12.3 Hz, 4β-H), 2.38 (4″-CH_3_), 2.51 (d, 1H, *J* = 15.0 Hz, 1β-H), 2.67 (dd, 1H, *J* = 16.8 Hz, *J* = 4.8 Hz, 4α-H), 3.65 (t, 1H, *J* = 8.4 Hz, 17α-H), 7.22 (d, 2H, *J* = 8.1 Hz, 3″-H and 5″-H), 7.30 (d, 2H, *J* = 8.1 Hz, 2″-H and 6″-H); ^13^C NMR (CDCl_3_, 125 MHz): δ 10.8 (5′-CH_3_), 11.0 (C-18), 11.8 (C-19), 20.8 (C-11), 21.0 (4″-CH_3_), 23.4 (C-15), 27.5 (C-4), 29.2 (C-6), 30.6 (C-16), 31.4 (C-7), 34.9 (C-1), 35.8 (C-8), 36.3 (C-10), 36.8 (C-12), 42.5 (C-5), 42.8 (C-13), 51.0 (C-14), 54.1 (C-9), 81.9 (C-17), 114.7 (C-2), 124.5 (2C, C-2″ and C-6″), 129.5 (2C, C-3″ and C-5″), 136.9, 137.2 and 137.4 (C-5′, C-1″ and C-4″), 148.2 (C-3); ESI-MS 419 [M+H]^+^.

17β-Hydroxy-1′-(2″,4″-dimethylphenyl)-5′-methylpyrazolo[3′,4′:3,2]-5α-androstane (**4d**)

According to the general procedure, 2,4-dimethylphenylhydrazine hydrochloride (**2d**, 207 mg) was used. Yield: 208 mg (80%, white solid); Mp 209–211 °C; Anal. Calcd. for C_29_H_40_N_2_O (432.65): C, 80.51; H, 9.32. Found: C, 80.40; H, 9.21. ^1^H NMR (CDCl_3_, 500 MHz): δ 0.77 (s, 3H, 18-H_3_), 0.81 (s, 3H, 19-H_3_), 0.84–1.02 (overlapping m, 3H, 9α-H, 7α-H and 14α-H), 1.11 (td, 1H, *J* = 12.7, *J* = 3.5 Hz, 12α-H), 1.28 (m, 1H, 15β-H), 1.34–1.51 (overlapping m, 4H, 6β-H, 8β-H, 16β-H and 11β-H), 1.55–1.65 (overlapping m, 3H, 5α-H, 15α-H and 6α-H), 1.66–1.75 (overlapping m, 2H, 11α-H and 7β-H), 1.85 (m, 1H, 12β-H), 1.93 (s, 3H), 1.98 (s, 3H), 2.07 (d + m, 2H, *J* = 15.0 Hz, 1α-H and 16α-H), 2.32 (m, 1H, 4β-H), 2.35 (s, 3H, 4″-CH_3_), 2.50 (d, 1H, *J* = 15.0 Hz, 1β-H), 2.65 (dd, 1H, *J* = 16.6 Hz, *J* = 4.4 Hz, 4α-H), 3.64 (t, 1H, *J* = 8.4 Hz, 17α-H), 7.01–7.10 (overlapping m, 3H, 3″-H, 5″-H and 6″-H); ^13^C NMR (CDCl_3_, 125 MHz): δ 9.7 (5′-CH_3_), 11.1 (C-18), 11.7 (C-19), 17.2 (2″-CH_3_), 20.8 (C-11), 21.1 (4″-CH_3_), 23.4 (C-15), 27.5 (C-4), 29.3 (C-6), 30.6 (C-16), 31.4 (C-7), 34.8 (C-1), 35.8 (C-8), 36.3 (C-10), 36.8 (C-12), 42.5 (C-5), 42.8 (C-13), 51.0 (C-14), 54.2 (C-9), 81.9 (C-17), 113.0 (C-2), 126.9, 127.7 and 131.4 (C-3″, C-5″ and C-6″), 135.8, 135.9, 136.2 and 138.6 (C-5′, C-1″, C-2″ and C-4″), 147.7 (C-3); ESI-MS 433 [M+H]^+^.

17β-Hydroxy-1′-(4″-methoxyphenyl)-5′-methylpyrazolo[3′,4′:3,2]-5α-androstane (**4e**)

According to the general procedure, 4-methoxyphenylhydrazine hydrochloride (**2e**, 210 mg) was used. Yield: 232 mg (89%, white solid); Mp 257–259 °C; Anal. Calcd. for C_28_H_38_N_2_O_2_ (434.62): C, 77.38; H, 8.81. Found: C, 77.49; H, 8.89. ^1^H NMR (CDCl_3_, 500 MHz): *δ* 0.77 (s, 3H, 18-H_3_), 0.80 (s, 3H, 19-H_3_), 0.84–1.02 (overlapping m, 3H, 9α-H, 7α-H and 14α-H), 1.11 (td, 1H, *J* = 12.7, *J* = 3.2 Hz, 12α-H), 1.28 (m, 1H, 15β-H), 1.33–1.50 (overlapping m, 4H, 6β-H, 8β-H, 16β-H and 11β-H), 1.52–1.65 (overlapping m, 3H, 5α-H, 15α-H and 6α-H), 1.67–1.75 (overlapping m, 2H, 11α-H and 7β-H), 1.86 (m, 1H, 12β-H), 2.06 (d + m, 2H, *J* = 15.0 Hz, 1α-H and 16α-H), 2.15 (s, 3H, 5′-CH_3_), 2.34 (dd, 1H, *J* = 16.5 Hz, *J* = 12.2 Hz, 4β-H), 2.50 (d, 1H, *J* = 15.0 Hz, 1β-H), 2.66 (dd, 1H, *J* = 16.5 Hz, *J* = 4.7 Hz, 4α-H), 3.65 (t, 1H, *J* = 8.4 Hz, 17α-H), 3.83 (4″-OMe), 6.94 (d, 2H, *J* = 8.4 Hz, 3″-H and 5″-H), 7.32 (d, 2H, *J* = 8.4 Hz, 2″-H and 6″-H); ^13^C NMR (CDCl_3_, 125 MHz): *δ* 10.7 (5′-CH_3_), 11.0 (C-18), 11.8 (C-19), 20.8 (C-11), 23.4 (C-15), 27.5 (C-4), 29.2 (C-6), 30.6 (C-16), 31.4 (C-7), 34.9 (C-1), 35.8 (C-8), 36.3 (C-10), 36.8 (C-12), 42.5 (C-5), 42.8 (C-13), 51.0 (C-14), 54.1 (C-9), 55.5 (4″-OMe), 81.9 (C-17), 114.1 (2C, C-2″ and C-6″), 114.4 (C-2), 126.1 (2C, C-3″ and C-5″), 133.1 (C-1″), 135.3 (C-5′), 148.0 (C-3), 158.6 (C-4″); ESI-MS 435 [M+H]^+^.

17β-Hydroxy-1′-(4″-fluorophenyl)-5′-methylpyrazolo[3′,4′:3,2]-5α-androstane (**4f**)

According to the general procedure, 4-fluorophenylhydrazine hydrochloride (**2f**, 195 mg) was used. Yield: 208 mg 82%, (white solid); Mp 244–246 °C; Anal. Calcd. for C_27_H_35_FN_2_O (422.59): C, 76.74; H, 8.35. Found: C, 76.85; H, 8.22. ^1^H NMR (CDCl_3_, 500 MHz): δ 0.77 (s, 3H, 18-H_3_), 0.80 (s, 3H, 19-H_3_), 0.84–1.01 (overlapping m, 3H, 9α-H, 7α-H and 14α-H), 1.11 (td, 1H, *J* = 12.8, *J* = 4.2 Hz, 12α-H), 1.28 (m, 1H, 15β-H), 1.34–1.50 (overlapping m, 4H, 6β-H, 8β-H, 16β-H and 11β-H), 1.53–1.65 (overlapping m, 3H, 5α-H, 15α-H and 6α-H), 1.66–1.75 (overlapping m, 2H, 11α-H and 7β-H), 1.86 (m, 1H, 12β-H), 2.06 (d + m, 2H, *J* = 15.1 Hz, 1α-H and 16α-H), 2.17 (s, 3H, 5′-CH_3_), 2.33 (dd, 1H, *J* = 16.8 Hz, *J* = 12.1 Hz, 4β-H), 2.51 (d, 1H, *J* = 15.1 Hz, 1β-H), 2.66 (dd, 1H, *J* = 16.5 Hz, *J* = 5.2 Hz, 4α-H), 3.64 (t, 1H, *J* = 8.5 Hz, 17α-H), 7.12 (t, 2H, *J* = 8.5 Hz, 3″-H and 5″-H), 7.39 (m, 2H, 2″-H and 6″-H); ^13^C NMR (CDCl_3_, 125 MHz): δ 10.7 (5′-CH_3_), 11.0 (C-18), 11.8 (C-19), 20.8 (C-11), 23.4 (C-15), 27.4 (C-4), 29.2 (C-6), 30.5 (C-16), 31.3 (C-7), 34.8 (C-1), 35.8 (C-8), 36.3 (C-10), 36.8 (C-12), 42.5 (C-5), 42.8 (C-13), 51.0 (C-14), 54.1 (C-9), 81.9 (C-17), 115.0 (C-2), 115.8 (2C, *J* = 23.0 Hz, C-3″ and C-5″), 126.4 (2C, *J* = 8.5 Hz, C-2″ and C-6″), 135.3 (C-5′), 136.1 (C-1″), 148.6 (C-3), 161.4 (J = 246.6 Hz, C-4″); ESI-MS 423 [M+H]^+^.

17β-Hydroxy-1′-(4″-chlorophenyl)-5′-methylpyrazolo[3′,4′:3,2]-5α-androstane (**4g**)

According to the general procedure, 4-chlorophenylhydrazine hydrochloride (**2g**, 215 mg) was used. Yield: 219 mg (83%, white solid); Mp 260–262 °C; Anal. Calcd. for C_27_H_35_ClN_2_O (439.04): C, 73.87; H, 8.04. Found: C, 73.73; H, 8.09. ^1^H NMR (CDCl_3_, 500 MHz): δ 0.77 (s, 3H, 18-H_3_), 0.79 (s, 3H, 19-H_3_), 0.83–1.01 (overlapping m, 3H, 9α-H, 7α-H and 14α-H), 1.11 (td, 1H, *J* = 12.8, *J* = 4.1 Hz, 12α-H), 1.27 (m, 1H, 15β-H), 1.33–1.50 (overlapping m, 4H, 6β-H, 8β-H, 16β-H and 11β-H), 1.53–1.65 (overlapping m, 3H, 5α-H, 15α-H and 6α-H), 1.66–1.75 (overlapping m, 2H, 11α-H and 7β-H), 1.86 (m, 1H, 12β-H), 2.05 (d + m, 2H, *J* = 15.0 Hz, 1α-H and 16α-H), 2.20 (s, 3H, 5′-CH_3_), 2.33 (dd, 1H, *J* = 16.8 Hz, *J* = 12.1 Hz, 4β-H), 2.51 (d, 1H, *J* = 15.0 Hz, 1β-H), 2.65 (dd, 1H, *J* = 16.5 Hz, *J* = 5.2 Hz, 4α-H), 3.64 (t, 1H, *J* = 8.6 Hz, 17α-H), 7.35–7.41 (overlapping m, 4H, 2″-H, 3″-H, 5″-H and 6″-H); ^13^C NMR (CDCl_3_, 125 MHz): δ 10.9 (5′-CH_3_), 11.0 (C-18), 11.8 (C-19), 20.8 (C-11), 23.4 (C-15), 27.4 (C-4), 29.2 (C-6), 30.5 (C-16), 31.3 (C-7), 34.8 (C-1), 35.8 (C-8), 36.2 (C-10), 36.7 (C-12), 42.5 (C-5), 42.8 (C-13), 50.9 (C-14), 54.1 (C-9), 81.9 (C-17), 115.4 (C-2), 125.5 (2C, C-2″ and C-6″), 129.1 (2C, C-3″ and C-5″), 132.5 (C-4″), 135.2 (C-5′), 138.5 (C-1″), 149.0 (C-3); ESI-MS 440 [M+H]^+^.

17β-Hydroxy-1′-(4″-bromophenyl)-5′-methylpyrazolo[3′,4′:3,2]-5α-androstane (**4h**)

According to the general procedure, 4-bromophenylhydrazine hydrochloride (**2h**, 268 mg) was used. Yield: 232 mg (80%, white solid); Mp 256–259 °C; Anal. Calcd. for C_27_H_35_BrN_2_O (483.49): C, 67.07; H, 7.30. Found: C, 67.16; H, 7.38. ^1^H NMR (CDCl_3_, 500 MHz): δ 0.77 (s, 3H, 18-H_3_), 0.79 (s, 3H, 19-H_3_), 0.83–1.01 (overlapping m, 3H, 9α-H, 7α-H and 14α-H), 1.11 (td, 1H, *J* = 12.7, *J* = 3.5 Hz, 12α-H), 1.27 (m, 1H, 15β-H), 1.33–1.50 (overlapping m, 4H, 6β-H, 8β-H, 16β-H and 11β-H), 1.52–1.64 (overlapping m, 3H, 5α-H, 15α-H and 6α-H), 1.66–1.75 (overlapping m, 2H, 11α-H and 7β-H), 1.86 (m, 1H, 12β-H), 2.05 (d + m, 2H, *J* = 15.0 Hz, 1α-H and 16α-H), 2.20 (s, 3H, 5′-CH_3_), 2.32 (dd, 1H, *J* = 16.6 Hz, *J* = 12.4 Hz, 4β-H), 2.51 (d, 1H, *J* = 15.0 Hz, 1β-H), 2.66 (dd, 1H, *J* = 16.6 Hz, *J* = 4.9 Hz, 4α-H), 3.64 (t, 1H, *J* = 8.6 Hz, 17α-H), 7.32 (d, 2H, *J* = 8.5 Hz, 3″-H and 5″-H), 7.55 (d, 2H, *J* = 8.5 Hz, 2″-H and 6″-H); ^13^C NMR (CDCl_3_, 125 MHz): δ 11.0 (5′-CH_3_), 11.0 (C-18), 11.8 (C-19), 20.8 (C-11), 23.4 (C-15), 27.4 (C-4), 29.2 (C-6), 30.6 (C-16), 31.3 (C-7), 34.8 (C-1), 35.8 (C-8), 36.2 (C-10), 36.8 (C-12), 42.4 (C-5), 42.8 (C-13), 50.9 (C-14), 54.1 (C-9), 81.9 (C-17), 115.5 (C-2), 120.4 (C-4″), 125.8 (2C, C-2″ and C-6″), 132.0 (2C, C-3″ and C-5″), 135.2 (C-5′), 138.9 (C-1″), 149.0 (C-3); ESI-MS 484 [M+H]^+^.

17β-Hydroxy-1′-(4″-cyanophenyl)-5′-methylpyrazolo[3′,4′:3,2]-5α-androstane (**4i**)

According to the general procedure, 4-cianophenylhydrazine hydrochloride (**2i**, 204 mg) was used. Yield: 209 mg (81%, white solid); Mp 247–250 °C; Anal. Calcd. for C_28_H_35_N_3_O (429.61): C, 78.28; H, 8.21. Found: C, 78.12; H, 8.15. ^1^H NMR (CDCl_3_, 500 MHz): δ 0.77 (s, 3H, 18-H_3_), 0.79 (s, 3H, 19-H_3_), 0.84–1.02 (overlapping m, 3H, 9α-H, 7α-H and 14α-H), 1.11 (td, 1H, *J* = 12.7, *J* = 3.6 Hz, 12α-H), 1.28 (m, 1H, 15β-H), 1.33–1.51 (overlapping m, 4H, 6β-H, 8β-H, 16β-H and 11β-H), 1.52–1.65 (overlapping m, 3H, 5α-H, 15α-H and 6α-H), 1.67–1.76 (overlapping m, 2H, 11α-H and 7β-H), 1.86 (m, 1H, 12β-H), 2.06 (d + m, 2H, *J* = 15.3 Hz, 1α-H and 16α-H), 2.29 (s, 3H, 5′-CH_3_), 2.34 (dd, 1H, *J* = 16.9 Hz, *J* = 12.4 Hz, 4β-H), 2.53 (d, 1H, *J* = 15.3 Hz, 1β-H), 2.67 (dd, 1H, *J* = 16.9 Hz, *J* = 5.0 Hz, 4α-H), 3.64 (t, 1H, *J* = 8.5 Hz, 17α-H), 7.61 (d, 2H, *J* = 8.9 Hz, 2″-H and 6″-H), 7.72 (d, 2H, *J* = 8.9 Hz, 3″-H and 5″-H); ^13^C NMR (CDCl_3_, 125 MHz): δ 11.0 (C-18), 11.5 (5′-CH_3_), 11.8 (C-19), 20.8 (C-11), 23.4 (C-15), 27.4 (C-4), 29.2 (C-6), 30.6 (C-16), 31.3 (C-7), 34.7 (C-1), 35.8 (C-8), 36.2 (C-10), 36.7 (C-12), 42.3 (C-5), 42.8 (C-13), 50.9 (C-14), 54.0 (C-9), 81.9 (C-17), 109.8 (C-4″), 117.0 (C-2), 118.4 (CN), 123.7 (2C, C-2″ and C-6″), 133.1 (2C, C-3″ and C-5″), 135.5 (C-5′), 143.4 (C-1″), 150.3 (C-3); ESI-MS 430 [M+H]^+^.

17β-Hydroxy-1′-(4″-nitrophenyl)-5′-methylpyrazolo[3′,4′:3,2]-5α-androstane (**4j**)

According to the general procedure, 4-nitrophenylhydrazine hydrochloride (**2j**, 228 mg) was used. Yield: 229 mg (85%, yellow solid); Mp 238–240 °C; Anal. Calcd. for C_27_H_35_N_3_O_3_ (449.60): C, 72.13; H, 7.85. Found: C, 72.04; H, 7.76. ^1^H NMR (CDCl_3_, 500 MHz): δ 0.77 (s, 3H, 18-H_3_), 0.80 (s, 3H, 19-H_3_), 0.84–1.02 (overlapping m, 3H, 9α-H, 7α-H and 14α-H), 1.12 (td, 1H, *J* = 12.7, *J* = 3.6 Hz, 12α-H), 1.28 (m, 1H, 15β-H), 1.33–1.51 (overlapping m, 4H, 6β-H, 8β-H, 16β-H and 11β-H), 1.53–1.66 (overlapping m, 3H, 5α-H, 15α-H and 6α-H), 1.67–1.76 (overlapping m, 2H, 11α-H and 7β-H), 1.87 (m, 1H, 12β-H), 2.06 (d + m, 2H, *J* = 15.0 Hz, 1α-H and 16α-H), 2.32 (s, 3H, 5′-CH_3_), 2.34 (m, 1H, 4β-H), 2.54 (d, 1H, *J* = 15.0 Hz, 1β-H), 2.67 (dd, 1H, *J* = 17.0 Hz, *J* = 5.1 Hz, 4α-H), 3.64 (t, 1H, *J* = 8.5 Hz, 17α-H), 7.67 (d, 2H, *J* = 9.0 Hz, 2″-H and 6″-H), 8.30 (d, 2H, *J* = 9.0 Hz, 3″-H and 5″-H); ^13^C NMR (CDCl_3_, 125 MHz): δ 11.0 (C-18), 11.6 (5′-CH_3_), 11.8 (C-19), 20.8 (C-11), 23.4 (C-15), 27.4 (C-4), 29.2 (C-6), 30.5 (C-16), 31.3 (C-7), 34.7 (C-1), 35.8 (C-8), 36.2 (C-10), 36.7 (C-12), 42.3 (C-5), 42.8 (C-13), 50.9 (C-14), 54.0 (C-9), 81.9 (C-17), 117.0 (C-2), 123.2 (2C, C-2″ and C-6″), 124.7 (2C, C-3″ and C-5″), 135.7 (C-5′), 145.0 and 145.4 (C-1″ and C-4″), 150.7 (C-3); ESI-MS 450 [M+H]^+^.

#### 3.1.4. General Procedure for the Synthesis of Compounds **5a–j** by Jones Oxidation

Compound **4a–j** (0.25 mmol) was dissolved in acetone (10 mL) and Jones reagent (0.2 mL) was added dropwise into the solution, which was then stirred at room temperature for 20 min, and after the given reaction time diluted with water (15 mL). The precipitate that formed was extracted with CH_2_Cl_2_ (3× 10 mL), and the combined organic phases were washed with water (10 mL), then dried over anhydrous Na_2_SO_4_ and concentrated in vacuo. The crude product was purified by column chromatography with EtOAc/CH_2_Cl_2_ = 2:98.

1′-Phenyl-5′-methylpyrazolo[3′,4′:3,2]-5α-androst-17-one (**5a**)

According to the general procedure, **4a** (101 mg) was used. Yield: 96 mg (96%, white solid); Mp 247–250 °C; Anal. Calcd. for C_27_H_34_N_2_O (402.58): C, 80.55; H, 8.51. Found: C, 80.60; H, 8.59. ^1^H NMR (CDCl_3_, 500 MHz): δ 0.83 (s, 3H, 19-H_3_), 0.90 (s, 3H, 18-H_3_), 0.94 (m, 1H), 1.04 (m, 1H), 1.27–1.35 (overlapping m, 2H), 1.37–1.63 (overlapping m, 5H), 1.69 (m, 1H), 1.79 (m, 1H), 1.83–1.89 (overlapping m, 2H), 1.98 (m, 1H), 2.08 (d + m, 2H, *J* = 15.2 Hz, 1α-H and 16α-H), 2.21 (s, 3H, 5′-CH_3_), 2.36 (dd, 1H, *J* = 16.7 Hz, *J* = 12.1 Hz, 4β-H), 2.46 (dd, 1H, *J* = 19.2 Hz, *J* = 8.8 Hz, 16β-H), 2.52 (d, 1H, *J* = 15.2 Hz, 1β-H_2_), 2.69 (dd, 1H, *J* = 16.7 Hz, *J* = 5.1 Hz, 4α-H), 7.31 (m, 1H, 4″-H), 7.43 (d, 4H, *J* = 4.4 Hz, 2″-H, 3″-H, 5″-H and 6″-H); ^13^C NMR (CDCl_3_, 125 MHz): δ 10.9 (5′-CH_3_), 11.7 (C-19), 13.7 (C-18), 20.5 (C-11), 21.8 (C-15), 27.5 (C-4), 29.1 (C-6), 30.6 (C-12), 31.6 (C-7), 34.8 (C-1), 35.3 (C-8), 35.8 (C-16), 36.4 (C-10), 42.5 (C-5), 47.6 (C-13), 51.4 (C-14), 54.1 (C-9), 114.8 (C-2), 124.5 (2C, C-2″ and C-6″), 127.0 (C-4″), 128.9 (2C, C-3″ and C-5″), 135.2 (C-5′), 139.9 (C-1″), 148.4 (C-3), 221.2 (C-17); ESI-MS 403 [M+H]^+^.

1′-(2″-Tolyl)-5′-methylpyrazolo[3′,4′:3,2]-5α-androst-17-one (**5b**)

According to the general procedure, **4b** (105 mg) was used. Yield: 97 mg (93%, white solid); Mp 245–250 °C; Anal. Calcd. for C_28_H_36_N_2_O (416.61): C, 80.73; H, 8.71. Found: C, 80.61; H, 8.77. ^1^H NMR (CDCl_3_, 500 MHz): δ 0.83 (s, 3H, 19-H_3_), 0.90 (s, 3H, 18-H_3_), 0.94 (m, 1H), 1.04 (m, 1H), 1.27–1.35 (overlapping m, 2H), 1.37–1.64 (overlapping m, 5H), 1.68 (m, 1H), 1.79 (m, 1H), 1.83–1.89 (overlapping m, 2H), 1.95 (s, 3H), 1.98 (m, 1H), 2.03 (s, 3H), 2.09 (d + m, 2H, *J* = 15.1 Hz, 1α-H and 16α-H), 2.36 (dd, 1H, *J* = 16.5 Hz, *J* = 12.2 Hz, 4β-H), 2.46 (dd, 1H, *J* = 19.2 Hz, *J* = 8.8 Hz, 16β-H), 2.52 (d, 1H, *J* = 15.1 Hz, 1β-H_2_), 2.68 (dd, 1H, *J* = 16.5 Hz, *J* = 4.9 Hz, 4α-H), 7.19–7.32 (overlapping m, 4H, 3″-H, 4″-H, 5″-H and 6″-H); ^13^C NMR (CDCl_3_, 125 MHz): δ 9.7 (5′-CH_3_), 11.7 (C-19), 13.7 (C-18), 17.3 (2″-CH_3_), 20.5 (C-11), 21.8 (C-15), 27.5 (C-4), 29.1 (C-6), 30.7 (C-12), 31.6 (C-7), 34.8 (C-1), 35.3 (C-8), 35.8 (C-16), 36.4 (C-10), 42.5 (C-5), 47.6 (C-13), 51.4 (C-14), 54.1 (C-9), 112.9 (C-2), 126.3 (CH), 127.9 (CH), 128.7 (CH), 130.7 (CH), 136.1, 136.2 and 138.8 (C-5′, C-1″ and C-2″), 147.7 (C-3), 221.2 (C-17); ESI-MS 417 [M+H]^+^.

1′-(4″-Tolyl)-5′-methylpyrazolo[3′,4′:3,2]-5α-androst-17-one (**5c**)

According to the general procedure, **4c** (105 mg) was used. Yield: 96 mg (92%, white solid); Mp 241–243 °C; Anal. Calcd. for C_28_H_36_N_2_O (416.61): C, 80.73; H, 8.71. Found: C, 80.79; H, 8.78. ^1^H NMR (CDCl_3_, 500 MHz): δ 0.83 (s, 3H, 19-H_3_), 0.90 (s, 3H, 18-H_3_), 0.93 (m, 1H), 1.04 (m, 1H), 1.28–1.35 (overlapping m, 2H), 1.38–1.63 (overlapping m, 5H), 1.69 (m, 1H), 1.79 (m, 1H), 1.84–1.90 (overlapping m, 2H), 1.98 (m, 1H), 2.09 (d + m, 2H, *J* = 15.0 Hz, 1α-H and 16α-H), 2.18 (s, 3H, 5′-CH_3_), 2.36 (m, 1H, 4β-H), 2.38 (4″-CH_3_), 2.46 (dd, 1H, *J* = 19.3 Hz, *J* = 9.0 Hz, 16β-H), 2.52 (d, 1H, *J* = 15.0 Hz, 1β-H_2_), 2.70 (dd, 1H, *J* = 16.7 Hz, *J* = 4.6 Hz, 4α-H), 7.23 (d, 2H, *J* = 8.0 Hz, 3″-H and 5″-H), 7.30 (d, 2H, *J* = 8.0 Hz, 2″-H and 6″-H); ^13^C NMR (CDCl_3_, 125 MHz): δ 10.8 (5′-CH_3_), 11.8 (C-19), 13.7 (C-18), 20.5 (C-11), 21.0 (4″-CH_3_), 21.8 (C-15), 27.4 (C-4), 29.0 (C-6), 30.6 (C-12), 31.6 (C-7), 34.8 (C-1), 35.3 (C-8), 35.8 (C-16), 36.4 (C-10), 42.5 (C-5), 47.6 (C-13), 51.4 (C-14), 54.1 (C-9), 114.5 (C-2), 124.5 (2C, C-2″ and C-6″), 129.5 (2C, C-3″ and C-5″), 136.9, 137.0 and 137.3 (C-5′, C-1″ and C-4″), 148.0 (C-3), 221.2 (C-17); ESI-MS 417 [M+H]^+^.

1′-(2″,4″-Dimethylphenyl)-5′-methylpyrazolo[3′,4′:3,2]-5α-androst-17-one (**5d**)

According to the general procedure, **4d** (108 mg) was used. Yield: 101 mg (94%, white solid); Mp 173–176 °C; Anal. Calcd. for C_29_H_38_N_2_O (430.64): C, 80.88; H, 8.89. Found: C, 80.98; H, 8.93. ^1^H NMR (CDCl_3_, 500 MHz): δ 0.82 (s, 3H, 19-H_3_), 0.90 (s, 3H, 18-H_3_), 0.94 (m, 1H), 1.04 (m, 1H), 1.28–1.35 (overlapping m, 2H), 1.37–1.63 (overlapping m, 5H), 1.68 (m, 1H), 1.79 (m, 1H), 1.84–1.90 (overlapping m, 2H), 1.94 (s, 3H), 1.99 (s + m, 4H), 2.09 (d + m, 2H, *J* = 15.0 Hz, 1α-H and 16α-H), 2.35 (s + m, 4H, 4″-CH_3_ and 4β-H), 2.46 (dd, 1H, *J* = 19.5 Hz, *J* = 9.0 Hz, 16β-H), 2.51 (d, 1H, *J* = 15.0 Hz, 1β-H_2_), 2.68 (dd, 1H, *J* = 16.7 Hz, *J* = 4.5 Hz, 4α-H), 7.01–7.10 (overlapping m, 3H, 3″-H, 5″-H and 6″-H); ^13^C NMR (CDCl_3_, 125 MHz): δ 9.7 (5′-CH_3_), 11.7 (C-19), 13.7 (C-18), 17.2 (2″-CH_3_), 20.5 (C-11), 21.1 (4″-CH_3_), 21.8 (C-15), 27.5 (C-4), 29.1 (C-6), 30.7 (C-12), 31.6 (C-7), 34.8 (C-1), 35.3 (C-8), 35.8 (C-16), 36.4 (C-10), 42.4 (C-5), 47.6 (C-13), 51.4 (C-14), 54.1 (C-9), 112.8 (C-2), 127.0, 127.6 and 131.4 (C-3″, C-5″ and C-6″), 135.8, 135.9, 136.3 and 138.7 (C-5′, C-1″, C-2″ and C-4″), 147.4 (C-3), 221.2 (C-17); ESI-MS 431 [M+H]^+^.

1′-(4″-Methoxyphenyl)-5′-methylpyrazolo[3′,4′:3,2]-5α-androst-17-one (**5e**)

According to the general procedure, **4e** (109 mg) was used. Yield: 99 mg (92%, white solid); Mp 225–226 °C; Anal. Calcd. for C_28_H_36_N_2_O_2_ (432.61): C, 77.74; H, 8.39. Found: C, 77.68; H, 8.48. ^1^H NMR (CDCl_3_, 500 MHz): δ 0.82 (s, 3H, 19-H_3_), 0.90 (s, 3H, 18-H_3_), 0.93 (m, 1H), 1.04 (m, 1H), 1.27–1.34 (overlapping m, 2H), 1.37–1.63 (overlapping m, 5H), 1.68 (m, 1H), 1.78 (m, 1H), 1.83–1.89 (overlapping m, 2H), 1.98 (m, 1H), 2.08 (d + m, 2H, *J* = 15.0 Hz, 1α-H and 16α-H), 2.15 (s, 3H, 5′-CH_3_), 2.36 (dd, 1H, *J* = 16.6 Hz, *J* = 12.3 Hz, 4β-H), 2.46 (dd, 1H, *J* = 19.4 Hz, *J* = 9.0 Hz, 16β-H), 2.51 (d, 1H, *J* = 15.2 Hz, 1β-H_2_), 2.70 (dd, 1H, *J* = 16.6 Hz, *J* = 4.8 Hz, 4α-H), 3.83 (4″-OMe), 6.94 (d, 2H, *J* = 8.8 Hz, 3″-H and 5″-H), 7.32 (d, 2H, *J* = 8.8 Hz, 2″-H and 6″-H); ^13^C NMR (CDCl_3_, 125 MHz): δ 10.6 (5′-CH_3_), 11.8 (C-19), 13.7 (C-18), 20.5 (C-11), 21.8 (C-15), 27.4 (C-4), 29.0 (C-6), 30.6 (C-12), 31.6 (C-7), 34.8 (C-1), 35.3 (C-8), 35.8 (C-16), 36.4 (C-10), 42.4 (C-5), 47.6 (C-13), 51.4 (C-14), 54.1 (C-9), 55.5 (4″-OMe), 114.1 (2C, C-2″ and C-6″), 114.2 (C-2), 126.2 (2C, C-3″ and C-5″), 132.3 (C-1″), 135.5 (C-5′), 147.7 (C-3), 158.7 (C-4″), 221.2 (C-17); ESI-MS 433 [M+H]^+^.

1′-(4″-Fluorophenyl)-5′-methylpyrazolo[3′,4′:3,2]-5α-androst-17-one (**5f**)

According to the general procedure, **4f** (106 mg) was used. Yield: 100 mg (95%, white solid); Mp 208–210 °C; Anal. Calcd. for C_27_H_33_FN_2_O (420.57): C, 77.11; H, 7.91. Found: C, 77.23; H, 7.99. ^1^H NMR (CDCl_3_, 500 MHz): δ 0.81 (s, 3H, 19-H_3_), 0.90 (s, 3H, 18-H_3_), 0.93 (m, 1H), 1.03 (m, 1H), 1.27–1.34 (overlapping m, 2H), 1.37–1.61 (overlapping m, 5H), 1.68 (m, 1H), 1.78 (m, 1H), 1.83–1.89 (overlapping m, 2H), 1.97 (m, 1H), 2.08 (d + m, 2H, *J* = 15.0 Hz, 1α-H and 16α-H), 2.17 (s, 3H, 5′-CH_3_), 2.36 (dd, 1H, *J* = 16.6 Hz, *J* = 12.3 Hz, 4β-H), 2.45 (dd, 1H, *J* = 19.2 Hz, *J* = 8.9 Hz, 16β-H), 2.51 (d, 1H, *J* = 15.2 Hz, 1β-H_2_), 2.67 (dd, 1H, *J* = 16.6 Hz, *J* = 4.9 Hz, 4α-H), 7.11 (t, 2H, *J* = 8.4 Hz, 3″-H and 5″-H), 7.38 (m, 2H, 2″-H and 6″-H); ^13^C NMR (CDCl_3_, 125 MHz): δ 10.6 (5′-CH_3_), 11.7 (C-19), 13.7 (C-18), 20.5 (C-11), 21.8 (C-15), 27.4 (C-4), 29.0 (C-6), 30.6 (C-12), 31.6 (C-7), 34.8 (C-1), 35.3 (C-8), 35.8 (C-16), 36.3 (C-10), 42.4 (C-5), 47.6 (C-13), 51.4 (C-14), 54.0 (C-9), 114.8 (C-2), 115.5 (2C, *J* = 22.8 Hz, C-3″ and C-5″), 126.3 (2C, *J* = 8.5 Hz, C-2″ and C-6″), 135.4 (C-5′), 136.1 (C-1″), 148.4 (C-3), 161.5 (J = 247.1 Hz, C-4″), 221.2 (C-17); ESI-MS 421 [M+H]^+^.

1′-(4″-Chlorophenyl)-5′-methylpyrazolo[3′,4′:3,2]-5α-androst-17-one (**5g**)

According to the general procedure, **4g** (110 mg) was used. Yield: 102 mg (93%, white solid); Mp 200–203 °C; Anal. Calcd. for C_27_H_33_ClN_2_O (437.02): C, 74.21; H, 7.61. Found: C, 74.09; H, 7.67. ^1^H NMR (CDCl_3_, 500 MHz): δ 0.81 (s, 3H, 19-H_3_), 0.90 (s, 3H, 18-H_3_), 0.93 (m, 1H), 1.04 (m, 1H), 1.27–1.35 (overlapping m, 2H), 1.37–1.62 (overlapping m, 5H), 1.69 (m, 1H), 1.78 (m, 1H), 1.83–1.89 (overlapping m, 2H), 1.98 (m, 1H), 2.08 (d + m, 2H, *J* = 15.0 Hz, 1α-H and 16α-H), 2.20 (s, 3H, 5′-CH_3_), 2.35 (dd, 1H, *J* = 16.8 Hz, *J* = 12.1 Hz, 4β-H), 2.46 (dd, 1H, *J* = 19.2 Hz, *J* = 8.9 Hz, 16β-H), 2.52 (d, 1H, *J* = 15.0 Hz, 1β-H_2_), 2.69 (dd, 1H, *J* = 16.8 Hz, *J* = 5.1 Hz, 4α-H), 7.35–7.42 (overlapping m, 4H, 2″-H, 3″-H, 5″-H and 6″-H); ^13^C NMR (CDCl_3_, 125 MHz): δ 10.9 (5′-CH_3_), 11.7 (C-19), 13.7 (C-18), 20.5 (C-11), 21.8 (C-15), 27.4 (C-4), 29.0 (C-6), 30.6 (C-12), 31.6 (C-7), 34.7 (C-1), 35.3 (C-8), 35.8 (C-16), 36.3 (C-10), 42.4 (C-5), 47.6 (C-13), 51.4 (C-14), 54.0 (C-9), 115.2 (C-2), 125.5 (2C, C-2″ and C-6″), 129.1 (2C, C-3″ and C-5″), 132.7 (C-4″), 135.4 (C-5′), 138.4 (C-1″), 148.7 (C-3), 221.2 (C-17); ESI-MS 438 [M+H]^+^.

1′-(4″-Bromophenyl)-5′-methylpyrazolo[3′,4′:3,2]-5α-androst-17-one (**5h**)

According to the general procedure, **4h** (121 mg) was used. Yield: 110 mg (92%, white solid); Mp 239–242 °C; Anal. Calcd. for C_27_H_33_BrN_2_O (481.48): C, 67.35; H, 6.91. Found: C, 67.50; H, 6.99. ^1^H NMR (CDCl_3_, 500 MHz): δ 0.81 (s, 3H, 19-H_3_), 0.90 (s, 3H, 18-H_3_), 0.93 (m, 1H), 1.04 (m, 1H), 1.27–1.34 (overlapping m, 2H), 1.37–1.62 (overlapping m, 5H), 1.69 (m, 1H), 1.78 (m, 1H), 1.84–1.89 (overlapping m, 2H), 1.98 (m, 1H), 2.07 (d + m, 2H, *J* = 15.0 Hz, 1α-H and 16α-H), 2.20 (s, 3H, 5′-CH_3_), 2.34 (dd, 1H, *J* = 16.7 Hz, *J* = 12.4 Hz, 4β-H), 2.46 (dd, 1H, *J* = 19.3 Hz, *J* = 8.9 Hz, 16β-H), 2.52 (d, 1H, *J* = 15.0 Hz, 1β-H_2_), 2.68 (dd, 1H, *J* = 16.7 Hz, *J* = 5.1 Hz, 4α-H), 7.32 (d, 2H, *J* = 8.6 Hz, 3″-H and 5″-H), 7.55 (d, 2H, *J* = 8.6 Hz, 2″-H and 6″-H); ^13^C NMR (CDCl_3_, 125 MHz): δ 11.0 (5′-CH_3_), 11.7 (C-19), 13.7 (C-18), 20.5 (C-11), 21.8 (C-15), 27.4 (C-4), 29.0 (C-6), 30.6 (C-12), 31.6 (C-7), 34.8 (C-1), 35.3 (C-8), 35.8 (C-16), 36.3 (C-10), 42.4 (C-5), 47.6 (C-13), 51.4 (C-14), 54.0 (C-9), 115.3 (C-2), 120.4 (C-4″), 125.8 (2C, C-2″ and C-6″), 132.0 (2C, C-3″ and C-5″), 135.2 (C-5′), 139.0 (C-1″), 148.9 (C-3), 221.2 (C-17); ESI-MS 482 [M+H]^+^.

1′-(4″-Cyanophenyl)-5′-methylpyrazolo[3′,4′:3,2]-5α-androst-17-one (**5i**)

According to the general procedure, **4i** (107 mg) was used. Yield: 98 mg (92%, white solid); Mp 234–237 °C; Anal. Calcd. for C_28_H_33_N_3_O (427.59): C, 78.65; H, 7.78. Found: C, 78.76; H, 7.87. ^1^H NMR (CDCl_3_, 500 MHz): δ 0.81 (s, 3H, 19-H_3_), 0.90 (s, 3H, 18-H_3_), 0.94 (m, 1H), 1.05 (m, 1H), 1.27–1.35 (overlapping m, 2H), 1.37–1.62 (overlapping m, 5H), 1.70 (m, 1H), 1.79 (m, 1H), 1.84–1.90 (overlapping m, 2H), 1.98 (m, 1H), 2.08 (d + m, 2H, *J* = 15.1 Hz, 1α-H and 16α-H), 2.29 (s, 3H, 5′-CH_3_), 2.35 (dd, 1H, *J* = 16.8 Hz, *J* = 12.4 Hz, 4β-H), 2.47 (dd, 1H, *J* = 19.2 Hz, *J* = 8.7 Hz, 16β-H), 2.54 (d, 1H, *J* = 15.1 Hz, 1β-H_2_), 2.69 (dd, 1H, *J* = 16.8 Hz, *J* = 5.1 Hz, 4α-H), 7.61 (d, 2H, *J* = 8.6 Hz, 3″-H and 5″-H), 7.72 (d, 2H, *J* = 8.6 Hz, 2″-H and 6″-H); ^13^C NMR (CDCl_3_, 125 MHz): δ 11.5 (5′-CH_3_), 11.8 (C-19), 13.7 (C-18), 20.5 (C-11), 21.8 (C-15), 27.4 (C-4), 29.0 (C-6), 30.6 (C-12), 31.6 (C-7), 34.7 (C-1), 35.3 (C-8), 35.8 (C-16), 36.3 (C-10), 42.3 (C-5), 47.6 (C-13), 51.4 (C-14), 54.0 (C-9), 109.8 (C-4″), 116.7 (C-2), 118.4 (CN), 123.7 (2C, C-2″ and C-6″), 133.1 (2C, C-3″ and C-5″), 135.4 (C-5′), 143.5 (C-1″), 150.3 (C-3); 221.1 (C-17); ESI-MS 428 [M+H]^+^.

1′-(4″-Nitrophenyl)-5′-methylpyrazolo[3′,4′:3,2]-5α-androst-17-one (**5j**)

According to the general procedure, **4j** (112 mg) was used. Yield: 107 mg (96%, yellow solid); Mp 140–143 °C; Anal. Calcd. for C_27_H_33_N_3_O_3_ (447.58): C, 72.46; H, 7.43. Found: C, 72.55; H, 7.34. ^1^H NMR (CDCl_3_, 500 MHz): δ 0.81 (s, 3H, 19-H_3_), 0.90 (s, 3H, 18-H_3_), 0.94 (m, 1H), 1.05 (m, 1H), 1.27–1.35 (overlapping m, 2H), 1.37–1.62 (overlapping m, 5H), 1.70 (m, 1H), 1.79 (m, 1H), 1.84–1.90 (overlapping m, 2H), 1.98 (m, 1H), 2.08 (d + m, 2H, *J* = 15.1 Hz, 1α-H and 16α-H), 2.33 (s, 3H, 5′-CH_3_), 2.35 (m, 1H, 4β-H), 2.46 (dd, 1H, *J* = 19.1 Hz, *J* = 8.7 Hz, 16β-H), 2.54 (d, 1H, *J* = 15.1 Hz, 1β-H_2_), 2.69 (dd, 1H, *J* = 16.8 Hz, *J* = 4.7 Hz, 4α-H), 7.66 (d, 2H, *J* = 8.9 Hz, 2″-H and 6″-H), 8.30 (d, 2H, *J* = 8.9 Hz, 3″-H and 5″-H); ^13^C NMR (CDCl_3_, 125 MHz): δ 11.7 (5′-CH_3_), 11.8 (C-19), 13.7 (C-18), 20.5 (C-11), 21.8 (C-15), 27.4 (C-4), 29.0 (C-6), 30.5 (C-12), 31.6 (C-7), 34.7 (C-1), 35.3 (C-8), 35.8 (C-16), 36.3 (C-10), 42.3 (C-5), 47.6 (C-13), 51.3 (C-14), 54.0 (C-9), 117.1 (C-2), 123.1 (2C, C-2″ and C-6″), 124.7 (2C, C-3″ and C-5″), 135.5 (C-5′), 145.2 and 145.3 (C-1″ and C-4″), 150.6 (C-3); 221.1 (C-17); ESI-MS 448 [M+H]^+^.

### 3.2. Pharmacology

#### 3.2.1. Cell Culture

HeLa, MCF-7 and MDA-MB-231 cells were obtained from Sigma-Aldrich and maintained as reported previously [22]. MRC-5, PC-3, DU 145 cells were purchased from American Type Culture Collection (ATCC, Manassas, VA, USA). PC-3 and DU 145 were maintained in RPMI medium (Sigma-Aldrich) whereas MRC-5 cells were maintained in DMEM medium (Sigma-Aldrich). All media were complemented with 10% Fetal bovine serum (FBS, Sigma-Aldrich) 2 mM L-glutamine, 0.01% streptomycin and 0.005% ampicillin (Sigma-Aldrich). Cells were cultured under standard conditions in a 37 °C incubator containing 5% CO_2_ in 95% humidity.

#### 3.2.2. Cell Viability Assay

Each test compound was dissolved in cell culture grade DMSO (Molar Chemicals, Halásztelek, Hungary) in a final concentration of 10 mM. Antiproliferative action of the synthesized steroids was investigated by MTT assay. An amount of 5000 cells/well were seeded into 96 well plates. Twenty-four hours post seeding, all cell lines were treated with each compound in 10 and 30 μM concentrations for 72 hours. To identify the IC_50_ values, seeded cells were exposed to selected compounds in 0, 0.5, 1, 2, 3, 5, 10 and 15 μM concentrations. After 72 hours, cells were washed with PBS and incubated with culture medium containing 0.5 mg/mL MTT (3-(4, 5-dimethylthiazolyl-2)-2,5-diphenyltetrazolium bromide) reagent (SERVA, GmbH, Heidelberg, Germany) for an hour at 37 °C. Formazan crystals were solubilized in DMSO and extinction was measured at 570 nm using a Synergy HTX plate reader (BioTech-Hungary, Budapest, Hungary). MTT assays were performed at least three times using four independent biological replicates. Data were normalized to solvent controls and the graphical representation of the obtained data as well as the calculation of IC_50_ values were carried out by GraphPad Prism 7 software (GraphPad Software; San Diego, CA, USA).

#### 3.2.3. Apoptosis Detection

PC-3 cells were seeded at 2 × 10^6^ cells/well density in 6-well plates. On the following day cells were treated with 5 µM of either compound **4e** or **4j** or with 200 µM of cisplatin (Accord Healthcare Ltd., Middlesex, UK). After 48-hour treatment, cells were collected and a Dead Cell Apoptosis Kit containing AnnexinV-FITC and propidium iodide (Life Technologies-Hungary, Budapest, Hungary) was used according to the manufacturer’s recommendation. Fluorescence intensities of at least 10,000 cells/sample were measured by FACSCalibur™ (BD Biosciences, Franklin Lakes, CA, USA) and data were analyzed by FlowJo V10 software (BD Biosciences, Franklin Lakes, NJ, USA). Experiments were repeated three times using triplicates.

#### 3.2.4. Reverse Transcription and Real-Time qPCR

PC-3 cells were seeded at 2 × 10^6^ cells/well density in 6 well plates. On the following day cells were treated with 5 µM of either compound **4e** or **4j** or with 200 µM of cisplatin. Cells were collected 48-hours post-treatment and total cellular RNA was isolated using RNeasy^®^ Mini Kit (QIAGEN, Hilden, Germany) according to the manufacturer’s recommendation. RNA (2 µg) was reverse transcribed (TaqMan^®^ Reverse Transcription kit, Applied Biosystems, Foster City, CA, USA) in 50 µL total volume. PCR reactions were performed on PicoReal™ Real-time PCR (Thermo Scientific, Walthan, MA, USA) using SYBRGreen qPCR Master Mix (Thermo Scientific) with an input of 1 µL cDNA. Each primer (Table 3) was used at 200 nM concentration. Relative transcript levels were determined by the ΔΔC_t_ analysis using GAPDH as reference gene. Experiments were repeated three times with three biological replicates.

#### 3.2.5. Statistical Analysis

Data are presented as the mean ± standard deviation (SD). GraphPad prism software was used to evaluate statistical differences by one-way analysis of variance (ANOVA) followed by Fisher’s LSD tests. Criterion for statistical significance was set at *p* < 0.05. The representative significance values as per Fisher’s LSD test are * *p* < 0.03, ** *p* < 0.002, *** *p* < 0.0002, **** *p* < 0.0001.

## 4. Conclusions

Despite numerous promising treatment modalities such as radiation therapy, surgical resection and hormone therapy, prostate cancer remains one of the top most common causes of cancer-related deaths among men worldwide. Prostate cancers that are insensitive to androgen blockade or those which are hormone-refractory after hormone and radiotherapy are the most challenging. In addition, the frequently observed loss of p53 function significantly affects the drug-induced apoptotic elimination of cancer cells. Current attempts focus on developing drugs capable of stimulating apoptosis in a p53-independent manner. For this purpose, a number of novel ring A-fused arylpyrazoles in the androstane series were synthesized and structurally characterized by 1D and 2D NMR methods. Heterocyclization reactions of a DHT-derived α,β-enone with arylhydrazines occurred regio-selectively both under conventional and MW heating conditions, but the resulting pyrazolines were obtained as mixtures of diastereomers due to the newly-formed chiral centers and were found to undergo at least partial autoxidation to pyrazoles. Therefore, the cyclizations were repeated under MW-assisted oxidative conditions in the presence of I_2_, which afforded the desired pyrazoles in good yields independently of the substituents of the reagents applied. The 17-oxo analogs of the heteroaromatic steroids were also prepared by Jones oxidation. According to the pharmacological studies, the novel ring A-fused arylpyrazole derivatives of DHT developed by our laboratory manifested a prominent structure-function relationship, where derivatives carrying a hydroxyl group on C-17 exhibited stronger anti-cancer activity compared to the 17-one counterparts. Moreover, some of the prepared analogs exhibited substantial cancer cell specific antiproliferative action and proapoptotic features on androgen therapy refractive p53-deficient PC-3 cells initiated by intrinsic pathways of apoptosis. Based on these conclusions further targeted design, synthesis and investigations of DHT analogs can be achieved.

## Figures and Tables

**Table 1:** Yields of ring A-fused arylpyrazole derivatives of DHT obtained by Method C (**4a–j**) and subsequent oxidation (**5a–j**).

Entry	R^1^	R^2^	Arylhydrazine	Compound	Yield (%) ^1^	Compound	Yield (%) ^1^
1	H	H	**2a**	**4a**	85	**5a**	96
2	CH_3_	H	**2b**	**4b**	88	**5b**	93
3	H	CH_3_	**2c**	**4c**	82	**5c**	92
4	CH_3_	CH_3_	**2d**	**4d**	80	**5d**	94
5	H	OMe	**2e**	**4e**	89	**5e**	92
6	H	F	**2f**	**4f**	82	**5f**	95
7	H	Cl	**2g**	**4g**	83	**5g**	93
8	H	Br	**2h**	**4h**	80	**5h**	92
9	H	CN	**2i**	**4i**	81	**5i**	92
10	H	NO_2_	**2j**	**4j**	85	**5j**	96

^1^ After purification by flash chromatography.

**Table 2:** IC_50_ values (µM ± SD) of the selected compounds determined on various cancer cell lines and on non-cancerous MRC-5 cells.

	4a	4e	4g	4j	5j
**MRC-5**	23.7 ± 2.3	8.6 ± 1.3	15.6 ± 1.4	>30	25.5 ± 2.3
**DU 145**	9.2 ± 1.2	3.6 ± 1.2	14.2 ± 1.3	19.7 ± 1	13.5 ± 1.1
**PC-3**	5.6 ±1.2	4.2 ± 1.1	7.7 ± 1.2	5.6 ± 1.1	6.7 ± 1.1
**HeLa**	16.9 ± 3.7	8.5 ± 0.6	7.2 ± 0.4	>30	10.4 ± 0.1
**MCF-7**	12.9 ± 0.1	5.5 ± 0.6	4.4 ± 0.3	18.2 ± 3.9	11.3 ± 2.8
**MDA-MB-231**	15.3 ± 3.4	6.6 ± 0.9	12.6 ± 1.5	>30	>30

**Table 3:** Primers and their sequences used for RT-qPCR analysis.

Target	FWD primer	REV primer
**Caspase-3**	5′-ACATGGCGTGTCATAAAATACC-3′	5′-CACAAAGCGACTGGATGAAC-3′
**Bax**	5′-TGCTTCAGGGTTTCATCCAG-3′	5′-GGCGGCAATCATCCTCTG-3′
**Gapdh**	5′-GCACCACCAACTGCTTAGC-3′	5′-GGCATGGACTGTGGTCATGAG-3′

## Supplementary Materials

Supplementary materials can be found at https://www.mdpi.com/1422-0067/20/9/2170/s1.
