# Supplementary material for: Anti-Cancer Activity of Novel Dihydrotestosterone-Derived Ring A-Condensed Pyrazoles on Androgen Non-Responsive Prostate Cancer Cell Lines
Source: Int J Mol Sci. 2019 May 2;20(9):2170. doi: 10.3390/ijms20092170 (PMC6539495; doi:10.3390/ijms20092170)
Supplement: Supplementary file 1 [file ijms-20-02170-s001.pdf]

Supplementary Material

for

Anti-Cancer Activity of Novel Dihydrotestosterone-Derived Ring A-condensed Pyrazoles on Androgen Non-Responsive Prostate Cancer Cell Lines

Gergő Mótyán,[1,‡] Mohana Krishna Gopisetty,[2,‡] Réka Eleonóra Kiss-Faludy,[1,3] Ágnes Kulmány,[3] István Zupkó,[3] Éva Frank*[,1], Mónika Kiricsi*[,2]

[1]*Department of Organic Chemistry, University of Szeged, Dóm tér 8, H-6720 Szeged, Hungary. E-mail: frank@chem.u-szeged.hu; Tel: +36-62-544275*

[2]*Department of Biochemistry and Molecular Biology, University of Szeged, Közép fasor 52., H-6726 Szeged, Hungary. E-mail: kiricsim@bio.u-szeged.hu; Tel: +36-62-546377*

[3]*Department of Pharmacodynamics and Biopharmacy, University of Szeged, Eötvös u. 6, H-6720 Szeged, Hungary*

**Corresponding authors.*

[‡]*Authors (G. Mótyán and MK. Gopisetty) contributed equally to this work.*

Table of Contents

1. Spectral data (^{1}H NMR, ^{13}C NMR, and 2D NMR) of the synthesized compounds S2–S27
2. Mean values of primary growth inhibitory screen used for heat map construction S28
3. Dose-response curves S29-S30

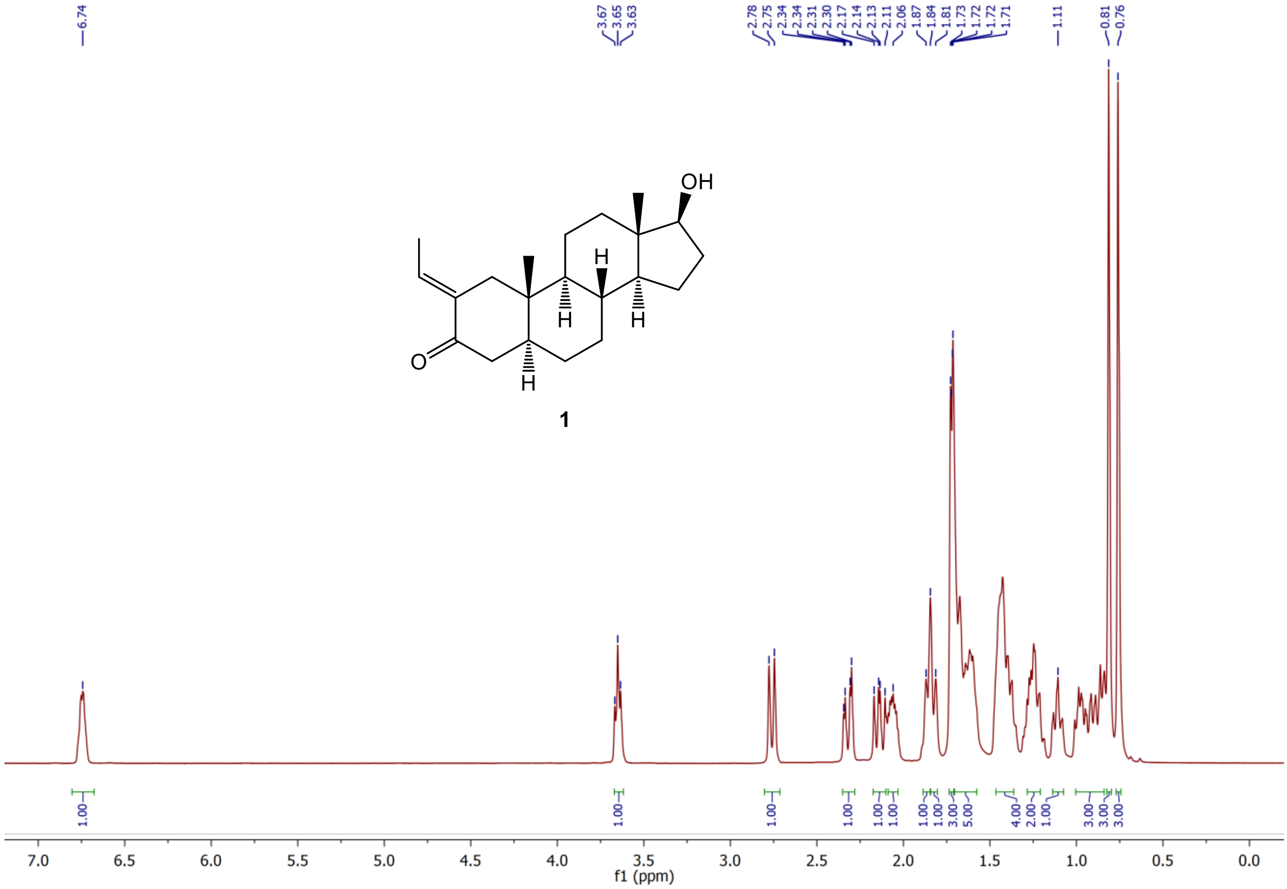
6.74
3.67 3.65 3.63
2.78 2.75 2.34 2.31 2.30 2.17 2.14 2.13 2.11 2.06 1.87 1.84 1.81 1.73 1.72 1.72 1.71
1.11
0.81 0.76
OH
H
H
H
O
H
1
1.00 1.00 1.00 1.00 1.00 1.00 1.00 1.00 3.00 5.00 4.00 2.00 1.00 3.00 3.00 3.00
7.0 6.5 6.0 5.5 5.0 4.5 4.0 3.5 3.0 2.5 2.0 1.5 1.0 0.5 0.0
f1 (ppm)

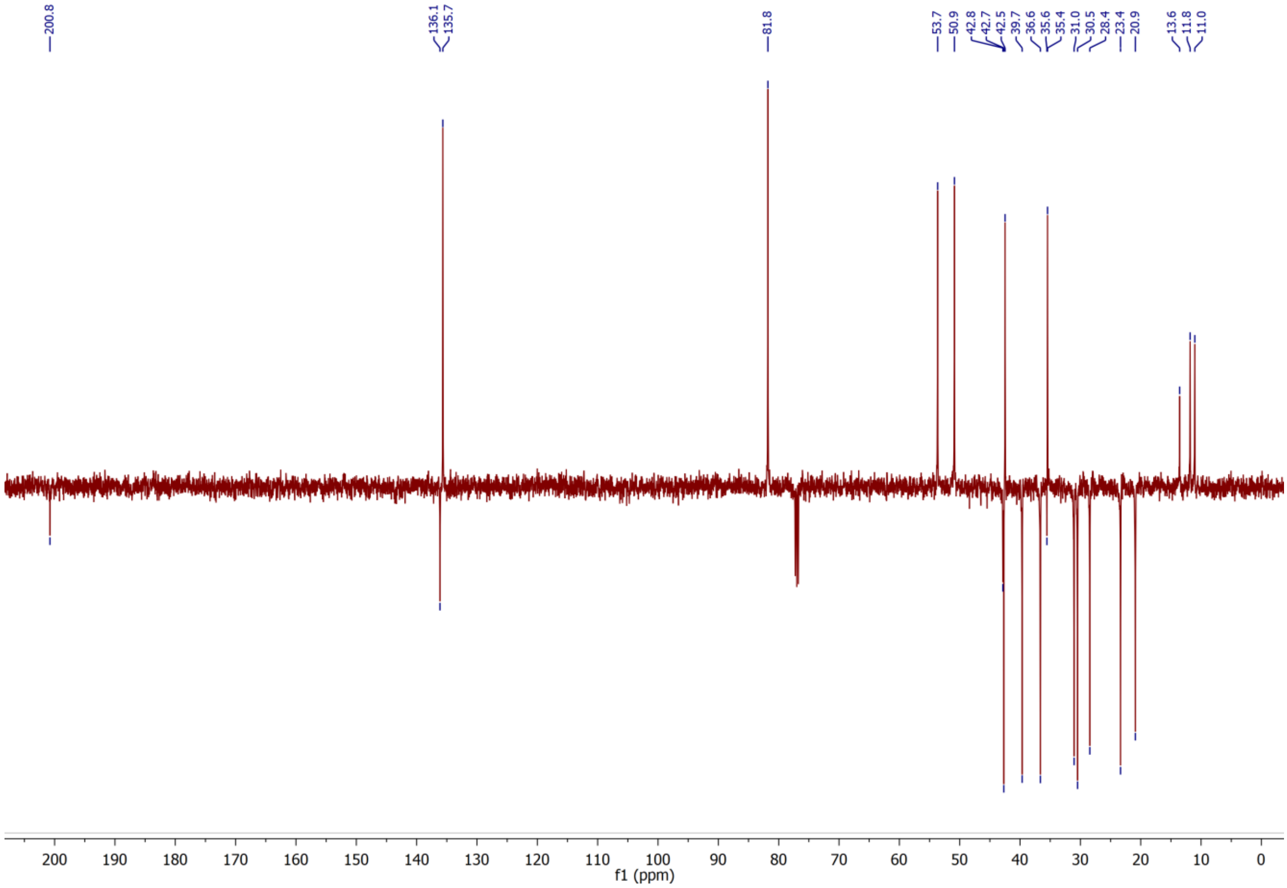
200.8
136.1 135.7
81.8
53.7 50.9 42.8 42.7 42.5 39.7 36.6 35.6 35.4 31.0 30.5 28.4 23.4 20.9
13.6 11.8 11.0
200 190 180 170 160 150 140 130 120 110 100 90 80 70 60 50 40 30 20 10 0
f1 (ppm)

Partial NOESY spectrum of compound **1**

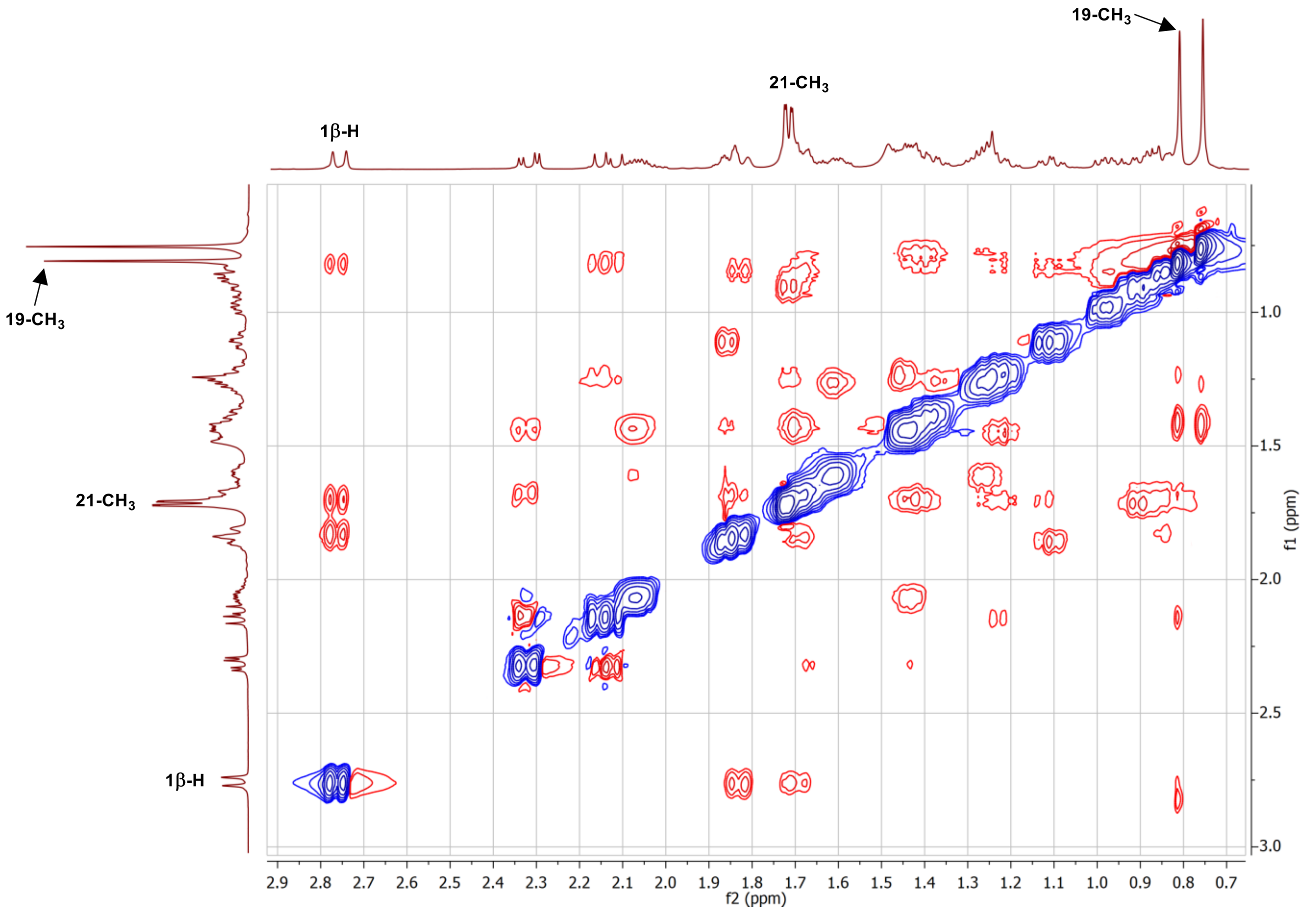

NOESY correlations between the 1β-H and 21-CH_3, and 1β-H and 18-CH_3 protons

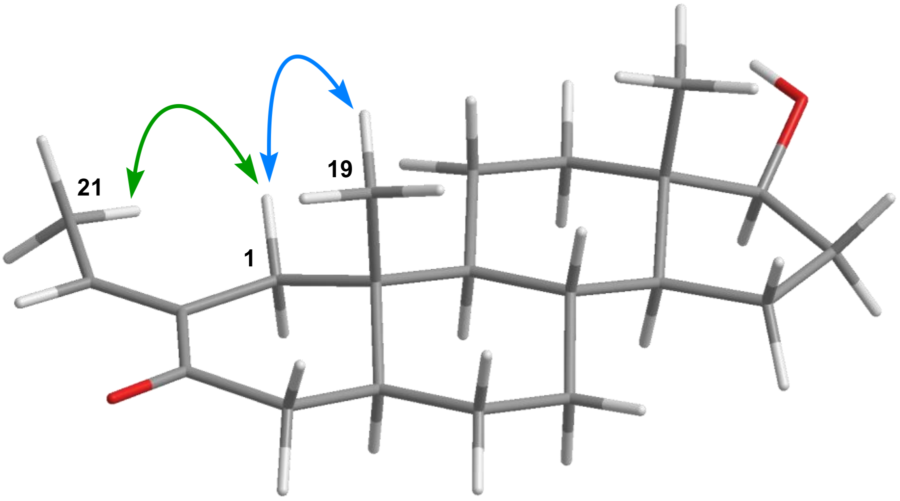

NOESY spectrum of compound **1**

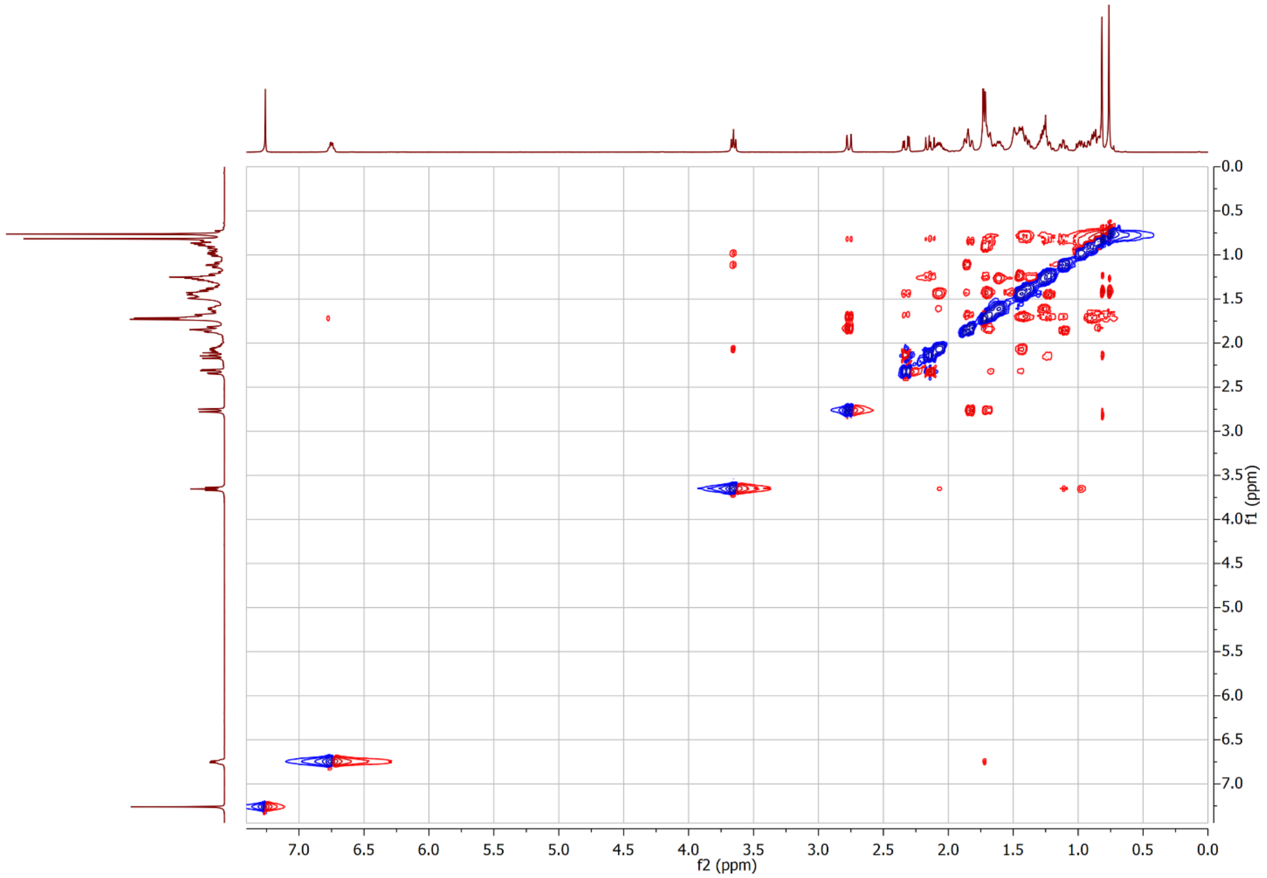

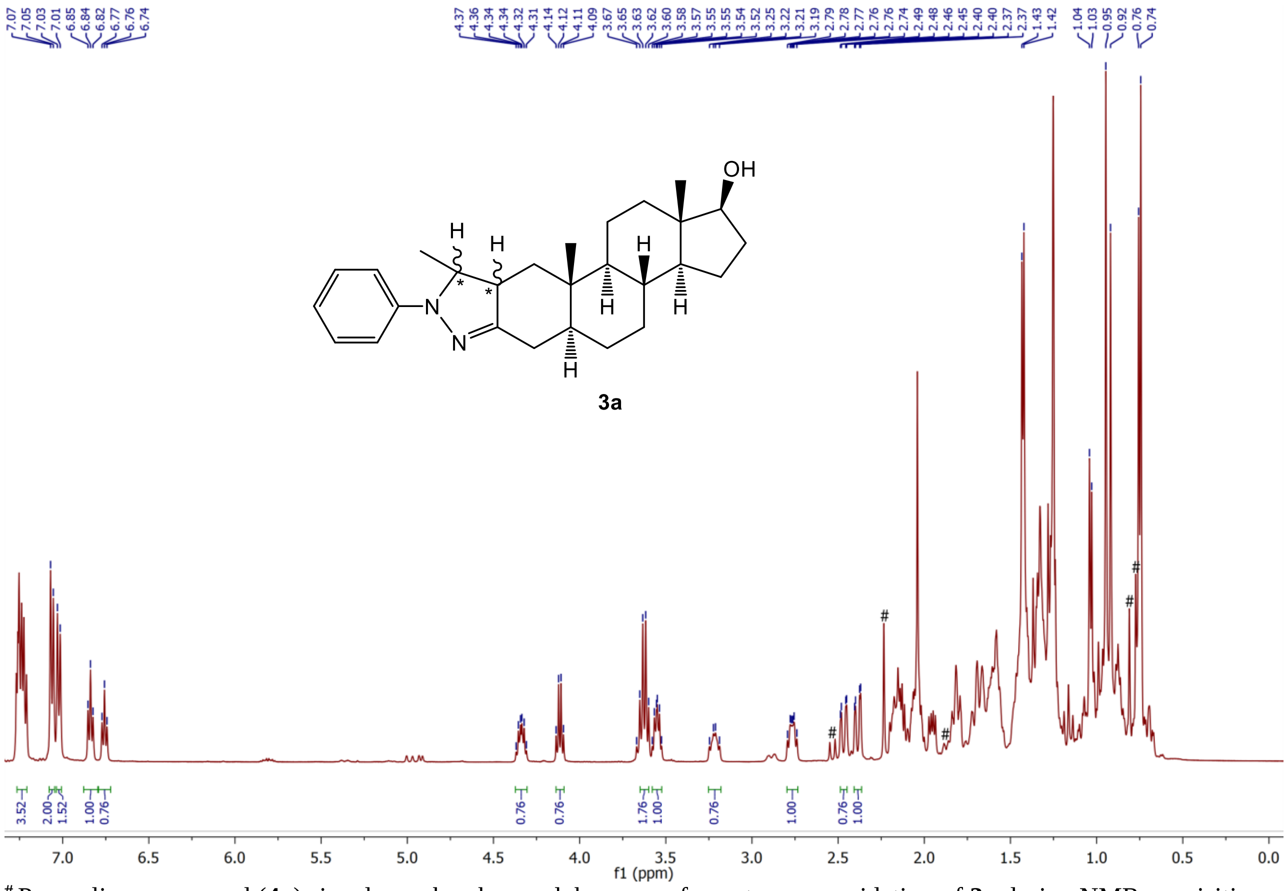

Pyrazoline compound (**4a**) signals can be observed, because of spontaneous oxidation of **3a** during NMR acquisition.

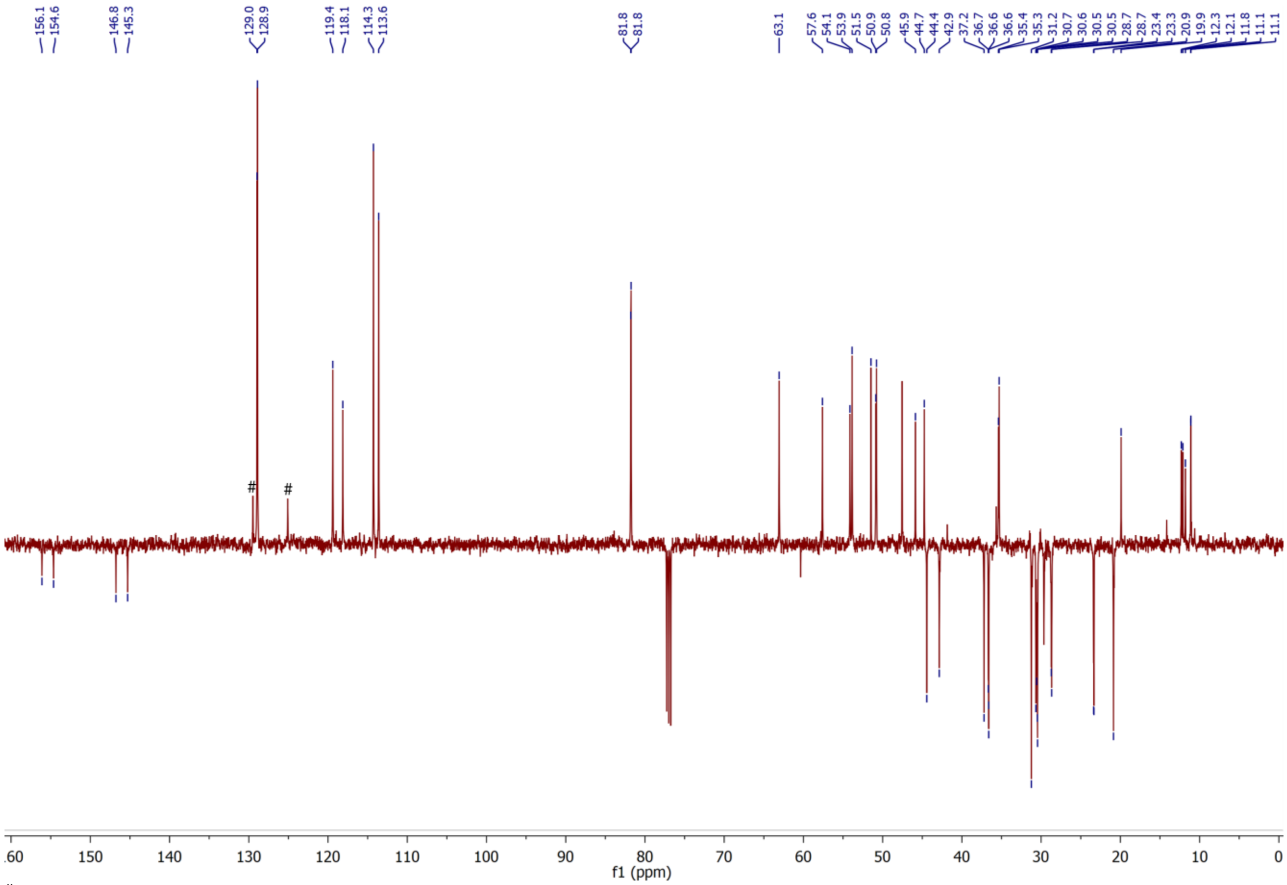

Pyrazoline compound (**4a**) signals can be observed, because of spontaneous oxidation of **3a** during NMR acquisition.

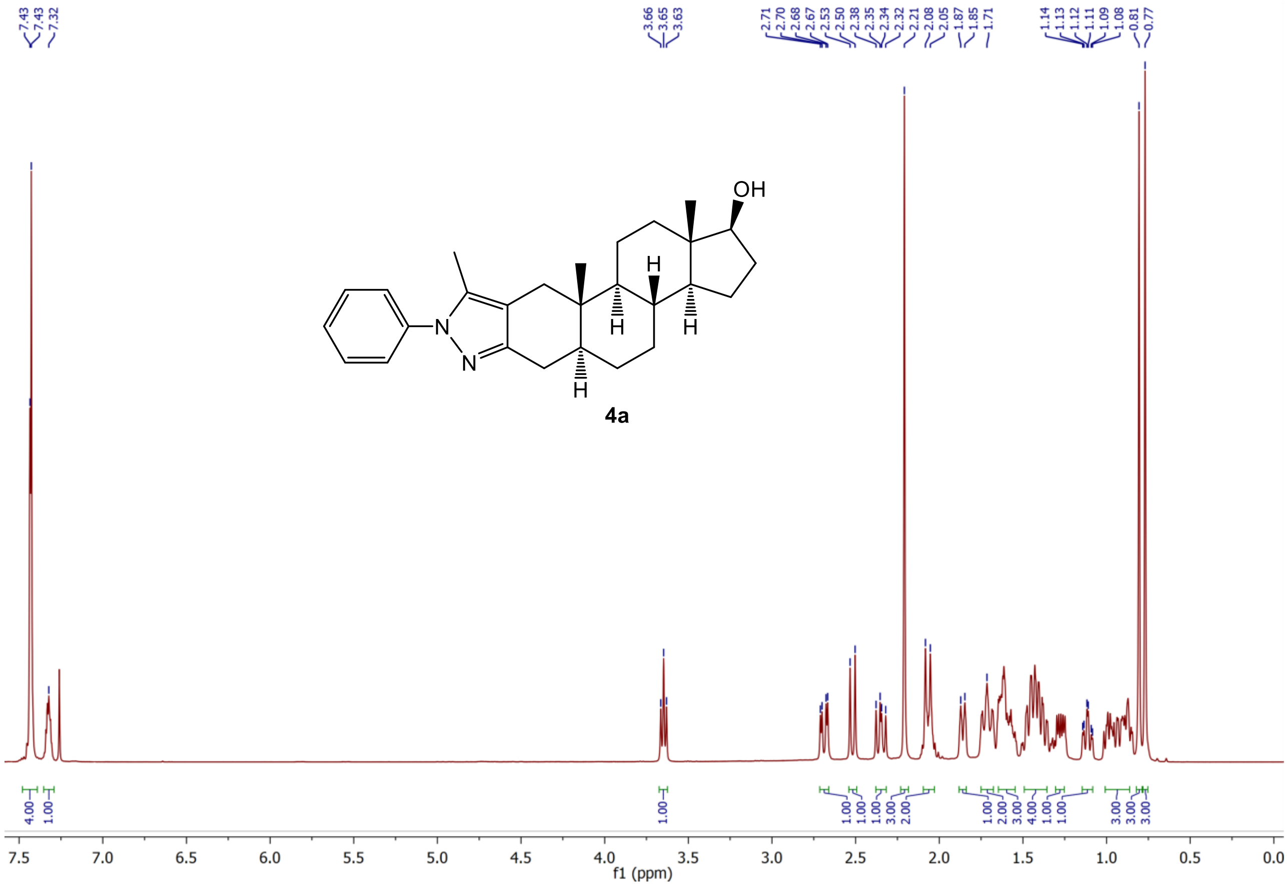
7.43
7.43
7.32
3.66
3.65
3.63
2.71
2.70
2.68
2.67
2.53
2.50
2.38
2.35
2.34
2.32
2.21
2.08
2.05
1.87
1.85
1.71
1.14
1.13
1.12
1.11
1.09
1.08
0.81
0.77
OH
H
H
H
H
N
N
4a
4.00
1.00
1.00
1.00
1.00
1.00
3.00
2.00
1.00
2.00
3.00
4.00
1.00
1.00
3.00
3.00
3.00
f1 (ppm)

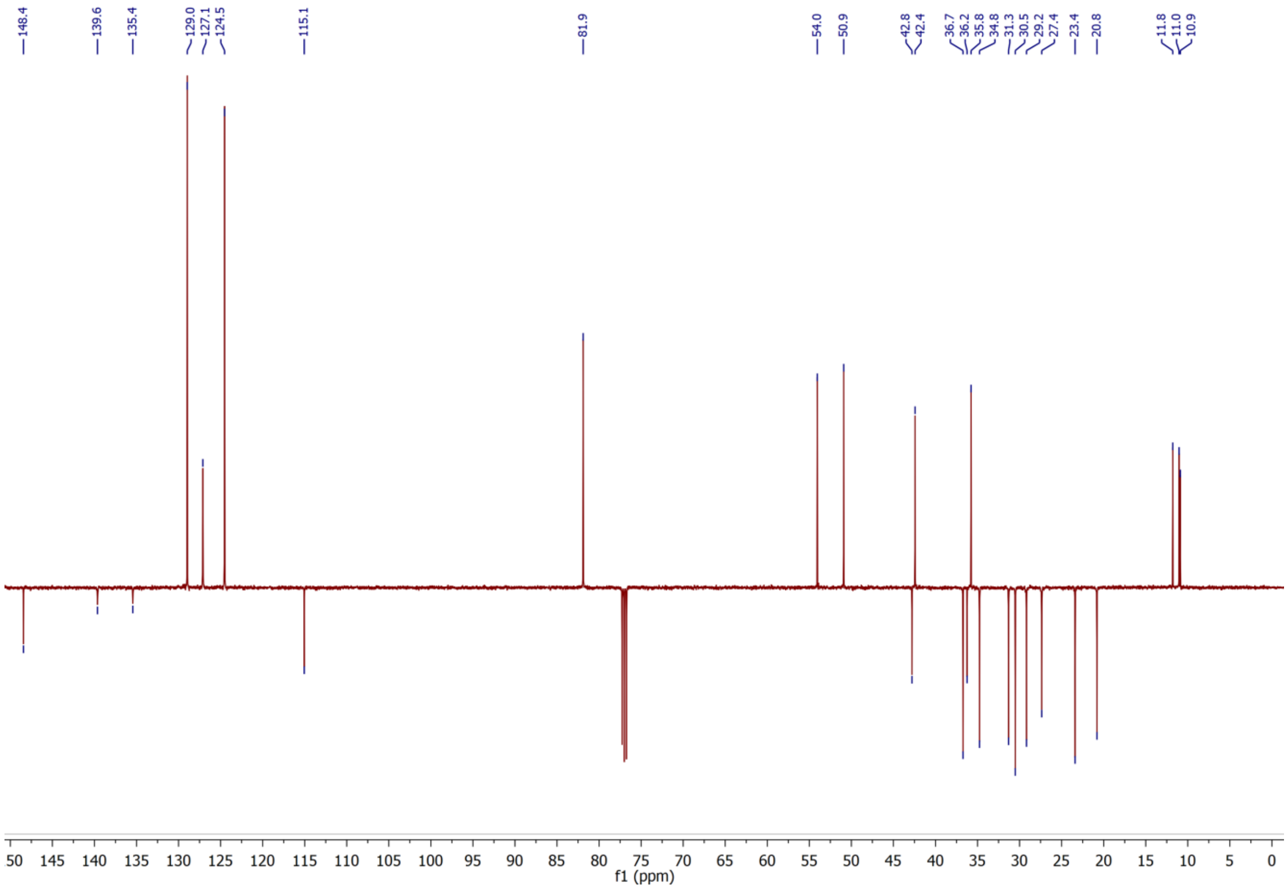
148.4
139.6
135.4
129.0
127.1
124.5
115.1
81.9
54.0
50.9
42.8
42.4
36.7
36.2
35.8
34.8
31.3
30.5
29.2
27.4
23.4
20.8
11.8
11.0
10.9
f1 (ppm)

HSQC spectrum of compound **4a**

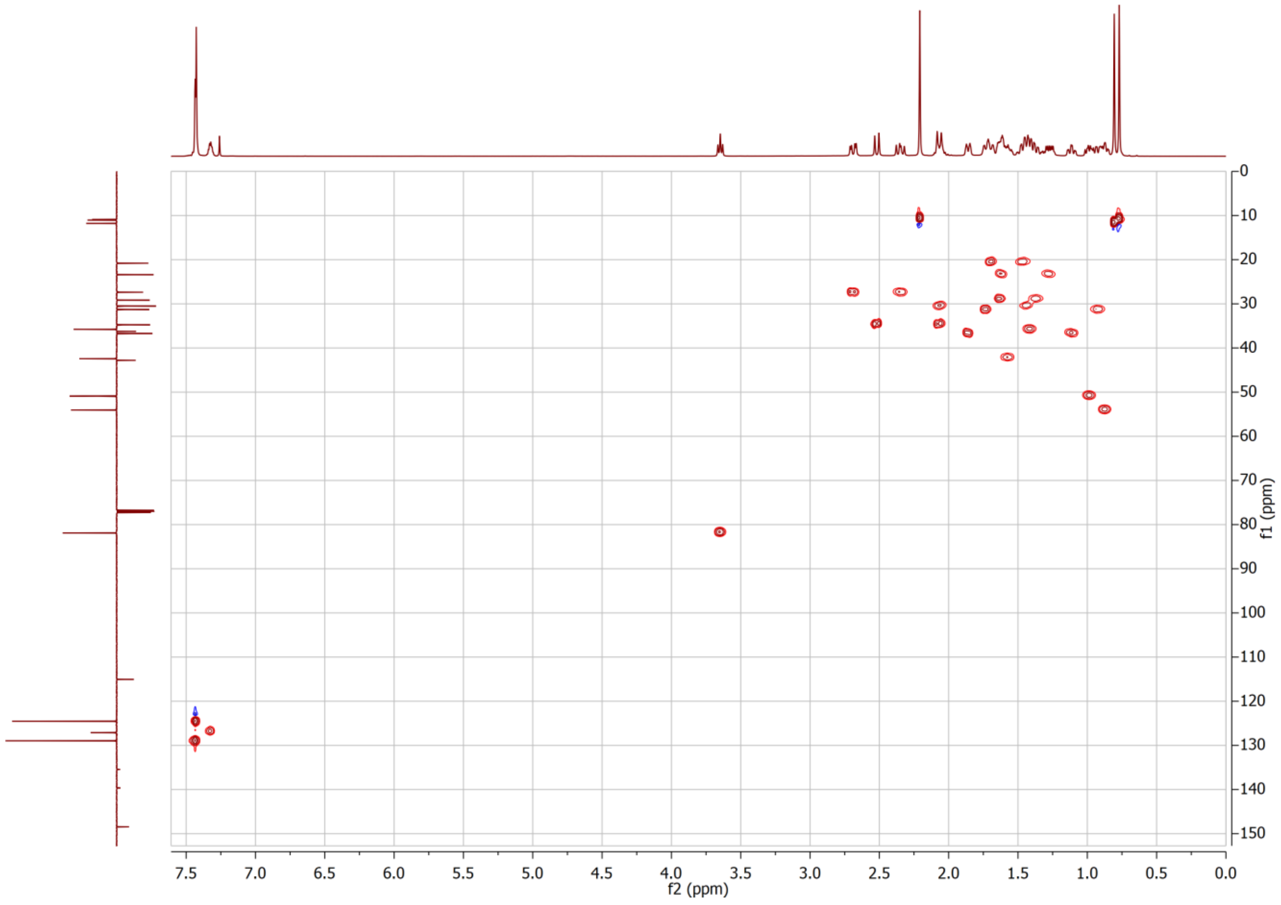

HMBC spectrum of compound **4a**

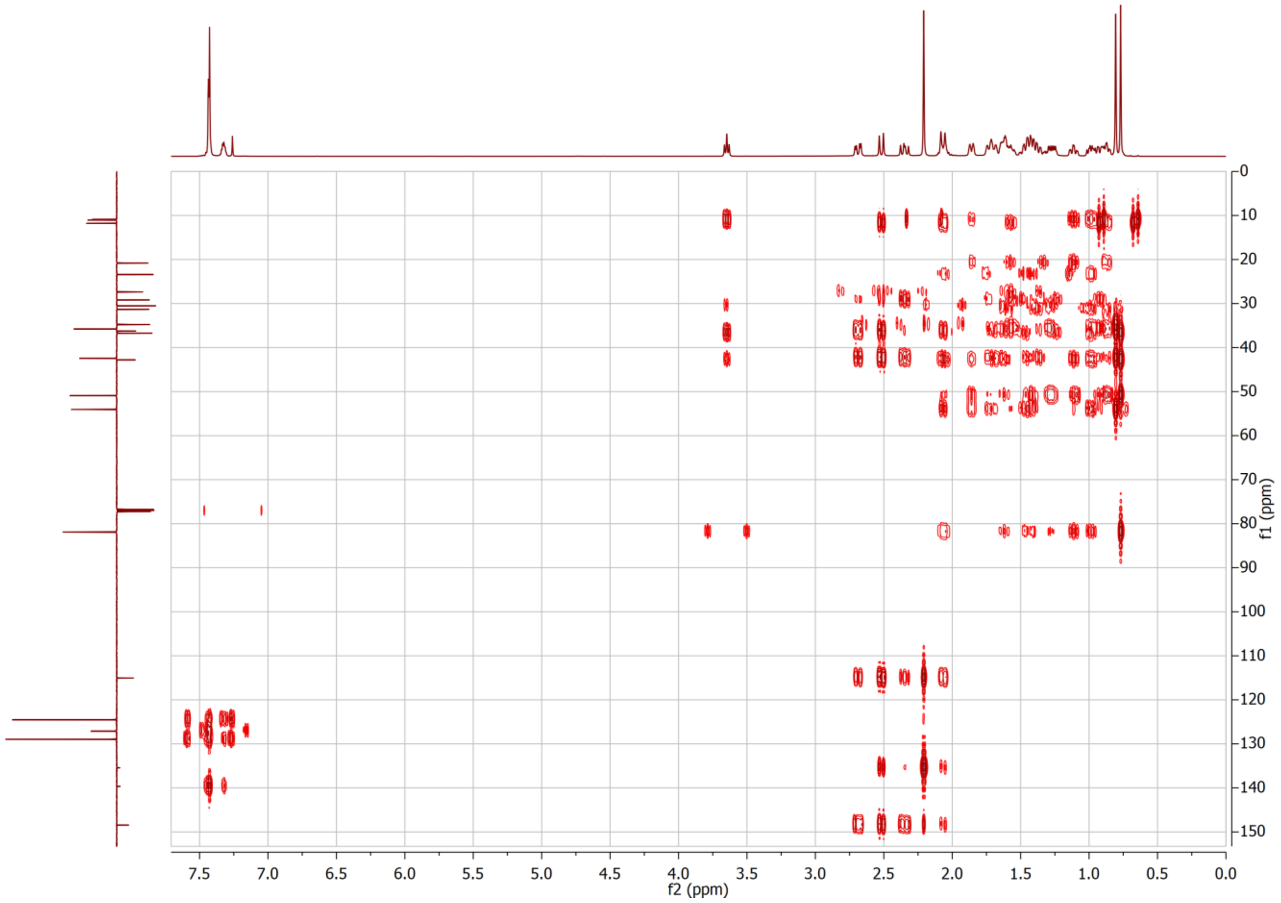

NOESY spectrum of compound **4a**

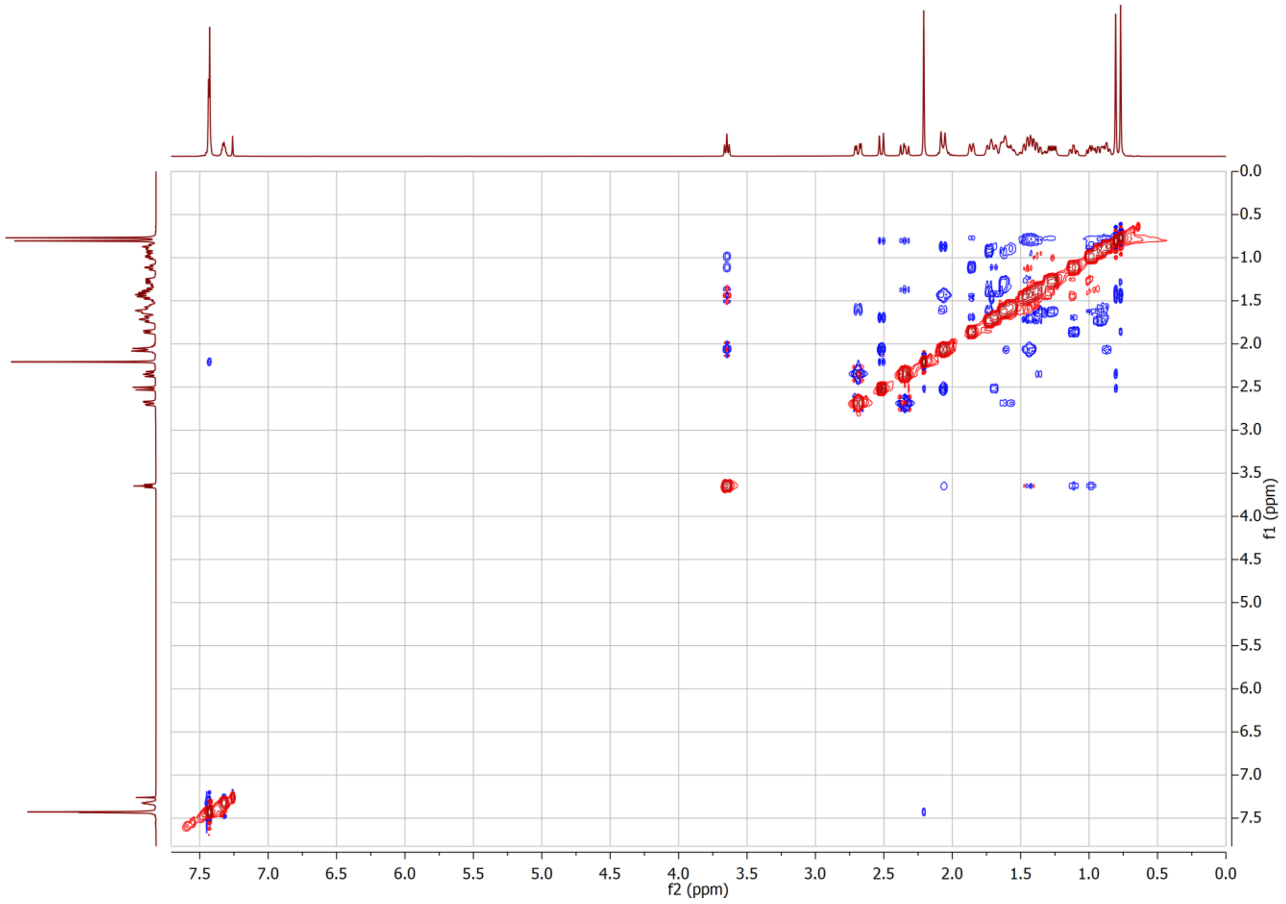

COSY spectrum of compound **4a**

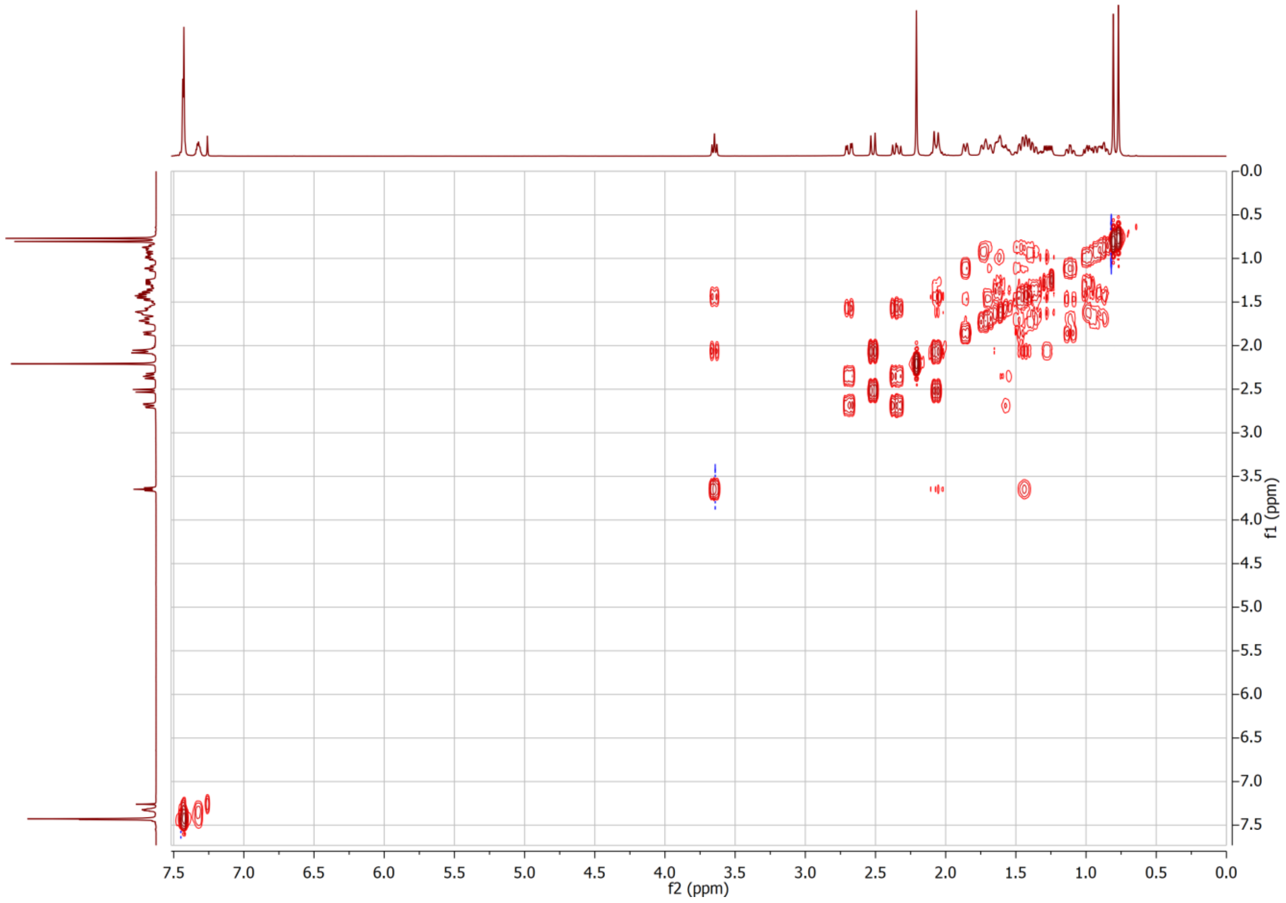

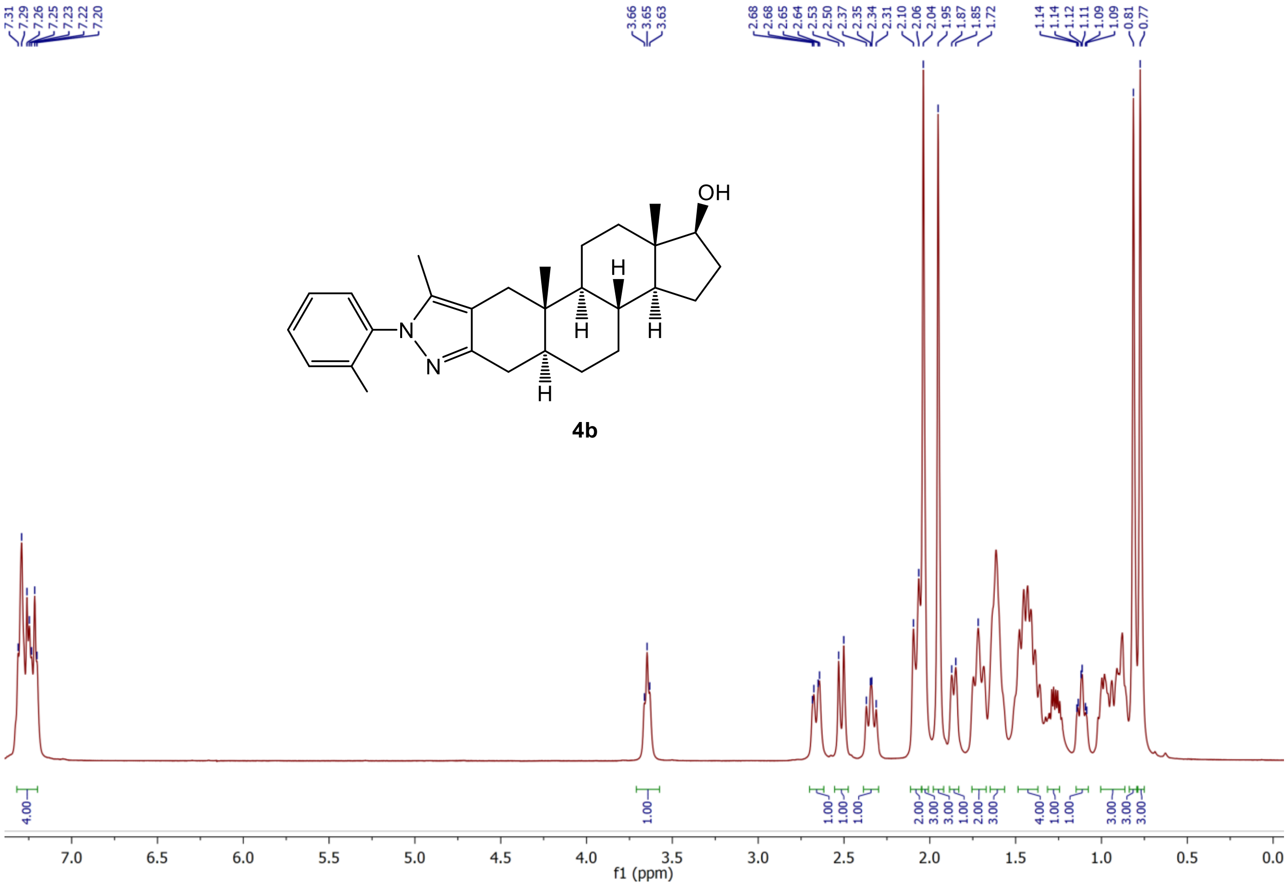
7.31
7.29
7.26
7.25
7.23
7.22
7.20
3.66
3.65
3.63
2.68
2.68
2.65
2.64
2.53
2.50
2.37
2.35
2.34
2.31
2.10
2.06
2.04
1.95
1.87
1.85
1.72
1.14
1.14
1.12
1.11
1.09
1.09
0.81
0.77
OH
H
H
H
H
N
N
4b
4.00
1.00
1.00
1.00
1.00
2.00
3.00
3.00
1.00
2.00
3.00
4.00
1.00
1.00
3.00
3.00
3.00
7.0 6.5 6.0 5.5 5.0 4.5 4.0 3.5 3.0 2.5 2.0 1.5 1.0 0.5 0.0
f1 (ppm)

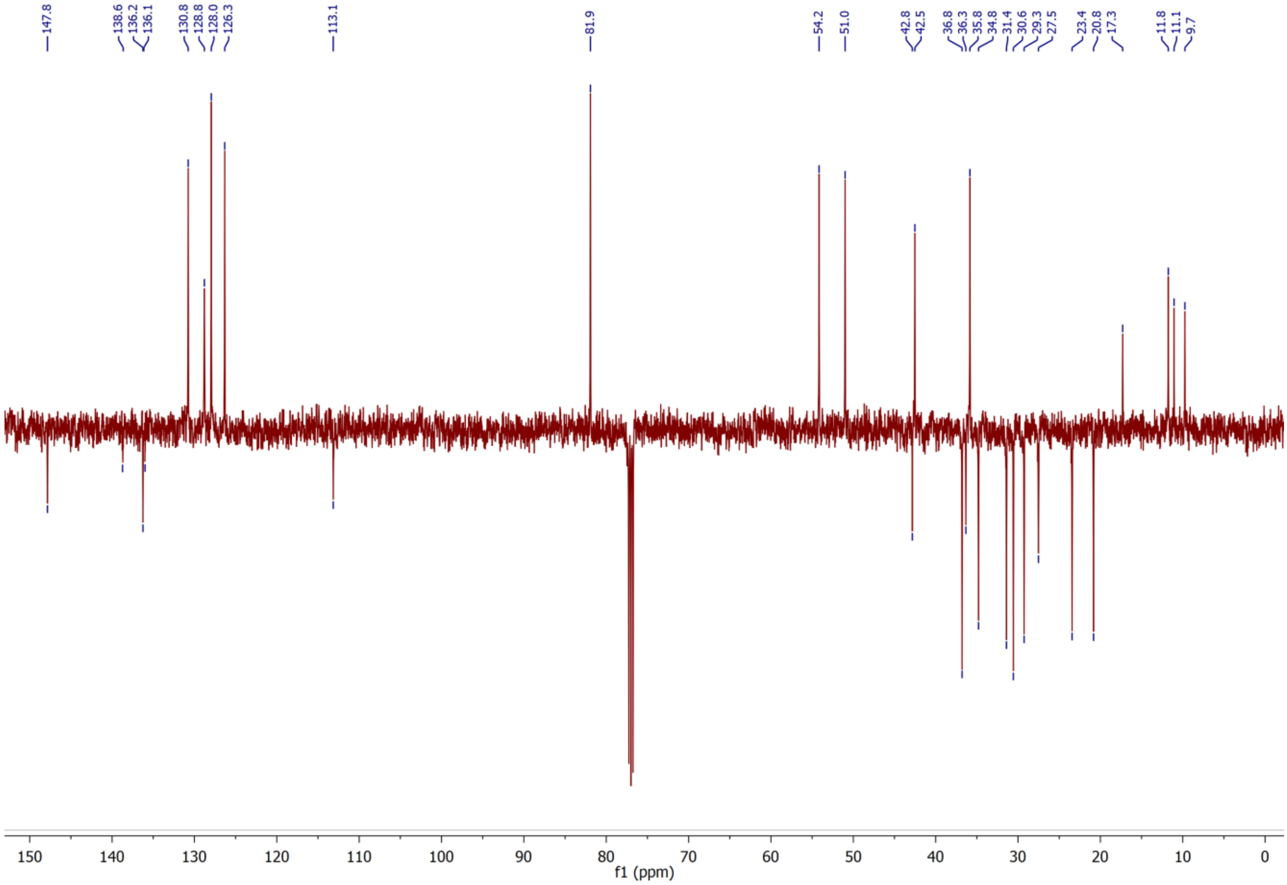
147.8
138.6
136.2
136.1
130.8
128.8
128.0
126.3
113.1
81.9
54.2
51.0
42.8
42.5
36.8
36.3
35.8
34.8
31.4
30.6
29.3
27.5
23.4
20.8
17.3
11.8
11.1
9.7
150 140 130 120 110 100 90 80 70 60 50 40 30 20 10 0
f1 (ppm)

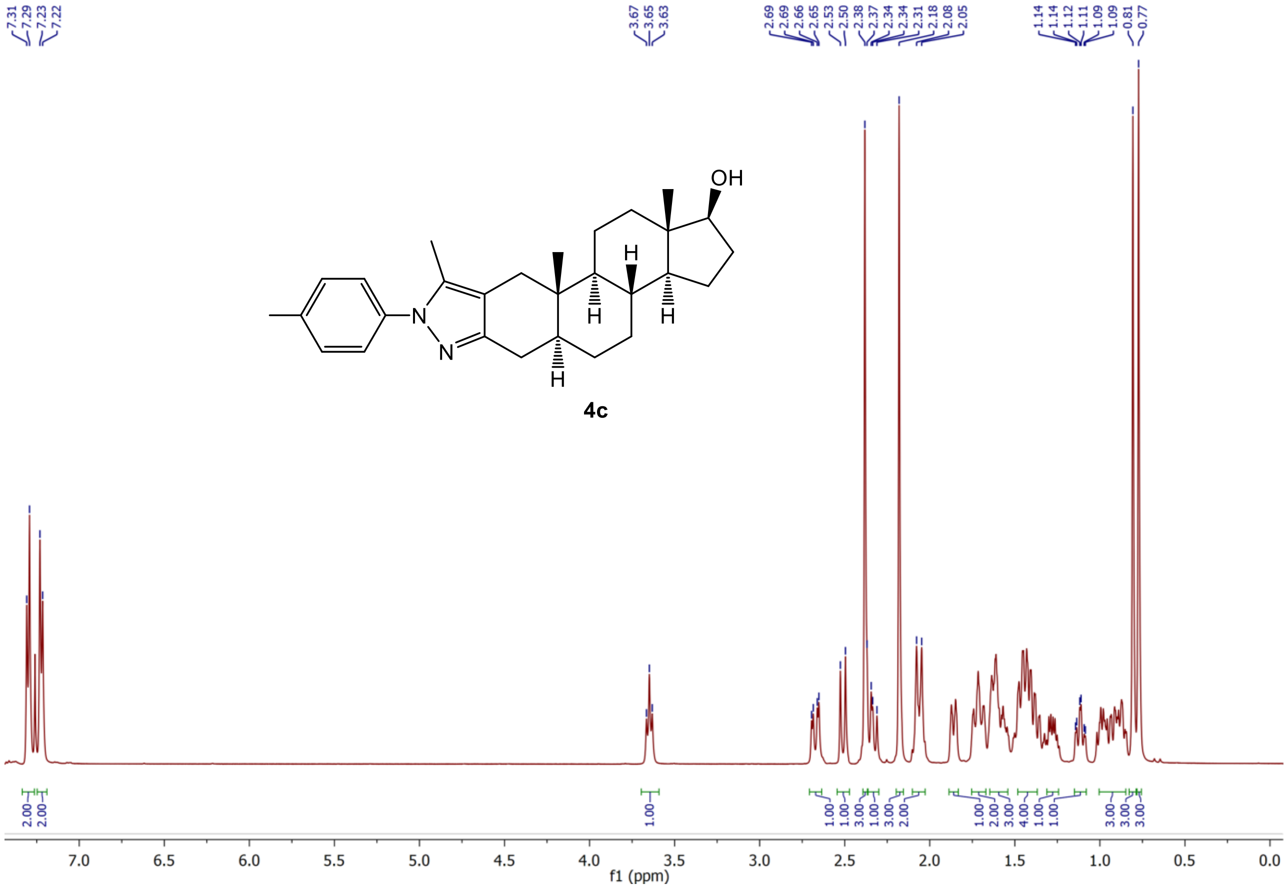
7.31
7.29
7.23
7.22
3.67
3.65
3.63
2.69
2.69
2.66
2.65
2.53
2.50
2.38
2.37
2.34
2.34
2.31
2.18
2.08
2.05
1.14
1.14
1.12
1.11
1.09
1.09
0.81
0.77
OH
H
H
H
H
N
N
4c
2.00
2.00
1.00
1.00
1.00
3.00
1.00
3.00
2.00
1.00
2.00
3.00
4.00
1.00
1.00
3.00
3.00
3.00
7.0
6.5
6.0
5.5
5.0
4.5
4.0
3.5
3.0
2.5
2.0
1.5
1.0
0.5
0.0
f1 (ppm)

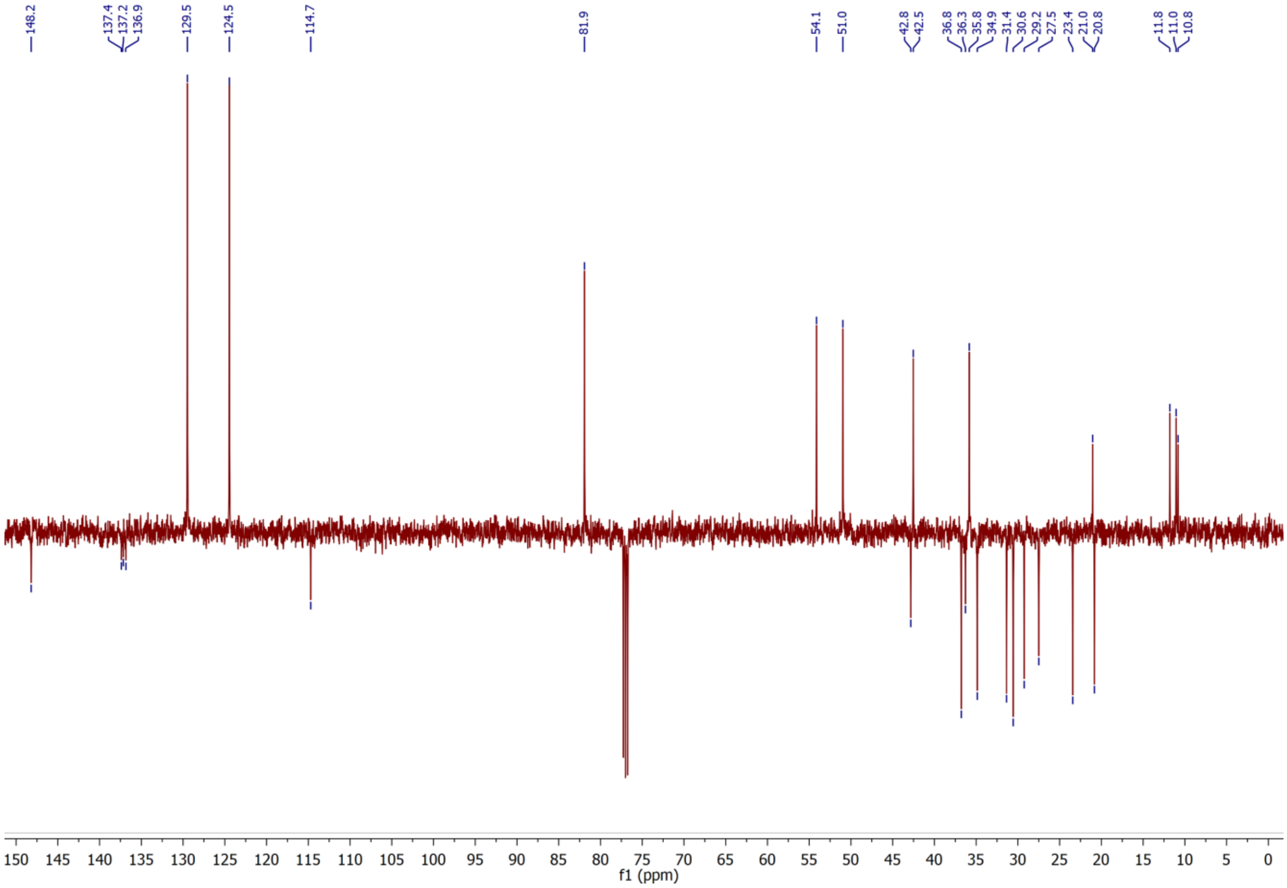
148.2
137.4
137.2
136.9
129.5
124.5
114.7
81.9
54.1
51.0
42.8
42.5
36.8
36.3
35.8
34.9
31.4
30.6
29.2
27.5
23.4
21.0
20.8
11.8
11.0
10.8
150
145
140
135
130
125
120
115
110
105
100
95
90
85
80
75
70
65
60
55
50
45
40
35
30
25
20
15
10
5
0
f1 (ppm)

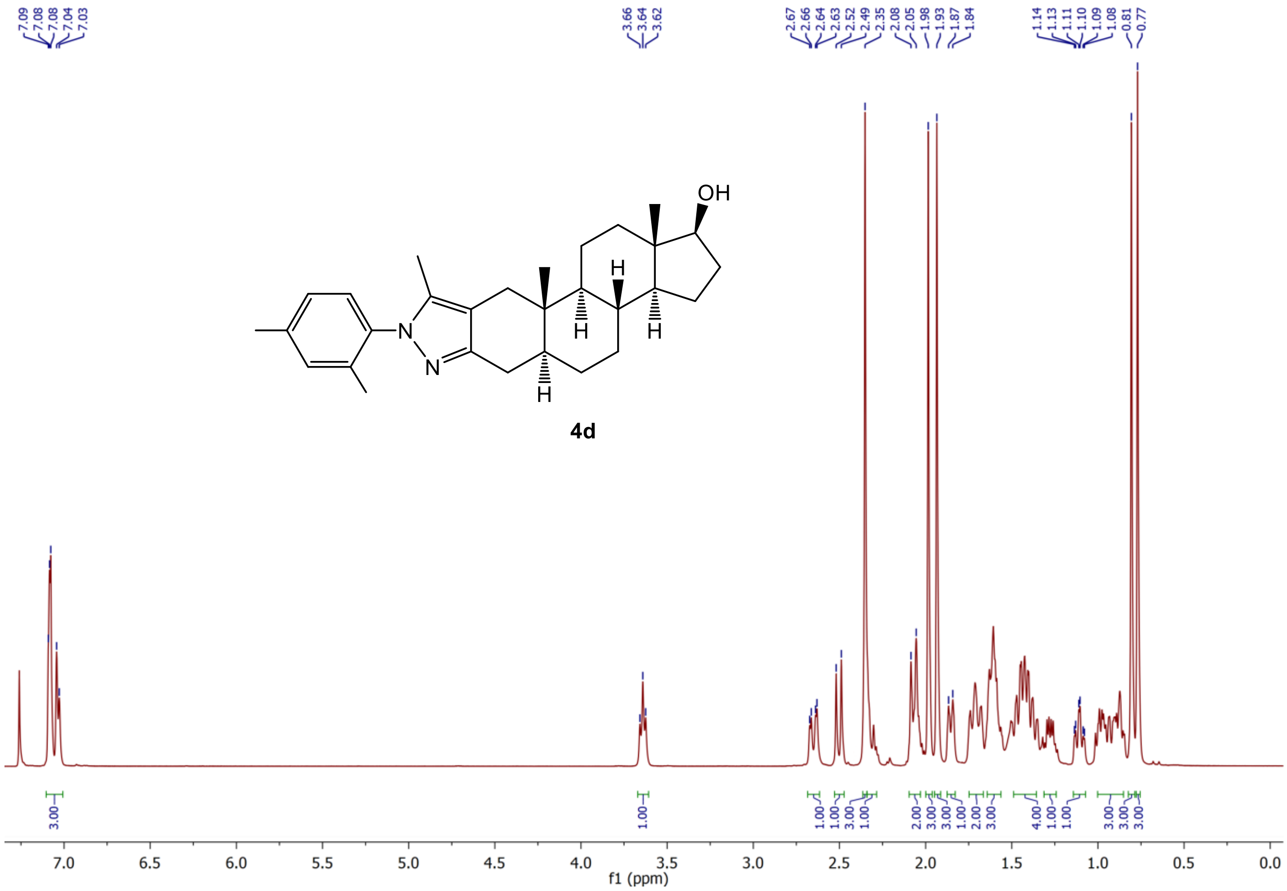
7.09
7.08
7.08
7.04
7.03
3.66
3.64
3.62
2.67
2.66
2.64
2.63
2.52
2.49
2.35
2.08
2.05
1.98
1.93
1.87
1.84
1.14
1.13
1.11
1.10
1.09
1.08
0.81
0.77
OH
H
H
H
H
N
N
4d
3.00
1.00
1.00
1.00
3.00
1.00
2.00
3.00
3.00
1.00
2.00
3.00
4.00
1.00
1.00
3.00
3.00
3.00
f1 (ppm)

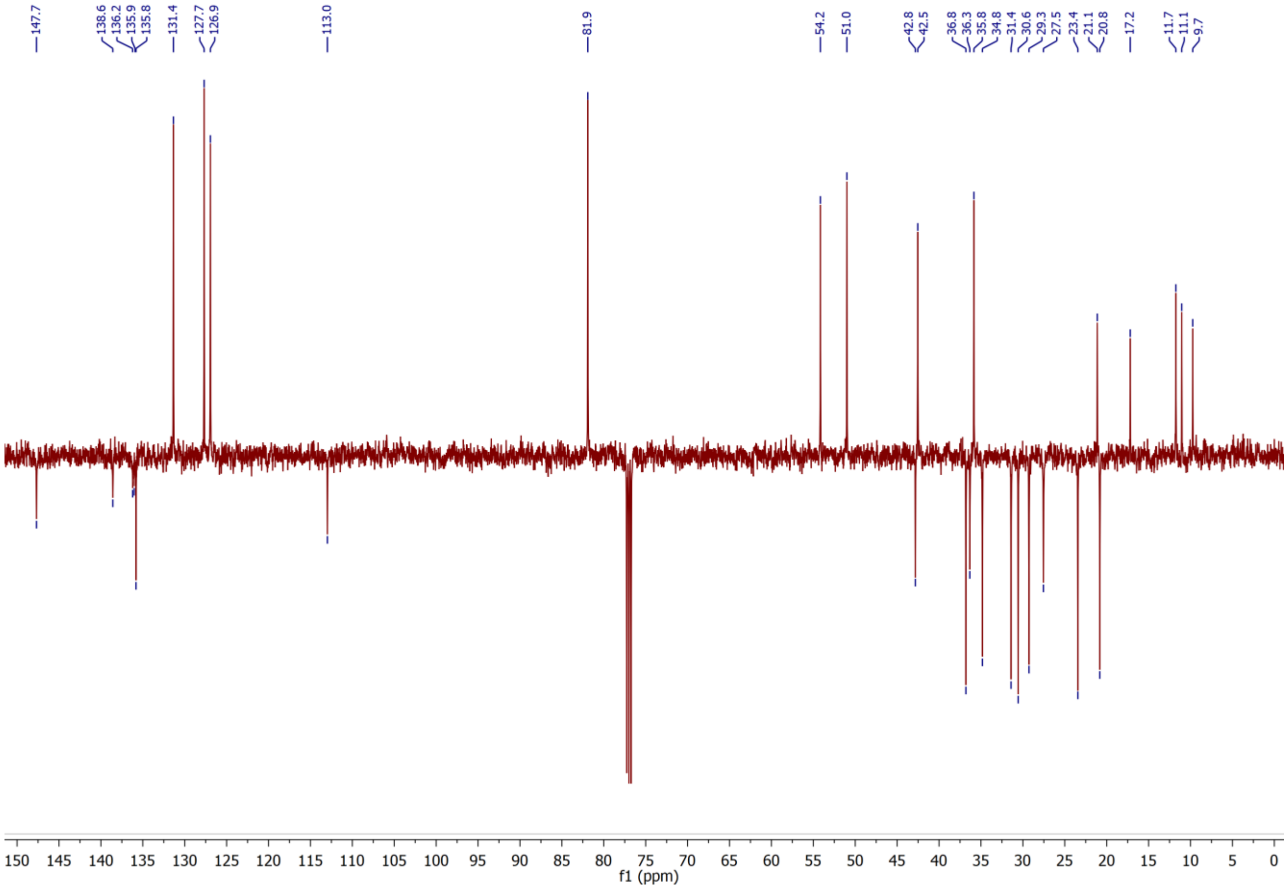
147.7
138.6
136.2
135.9
135.8
131.4
127.7
126.9
113.0
81.9
54.2
51.0
42.8
42.5
36.8
36.3
35.8
34.8
31.4
30.6
29.3
27.5
23.4
21.1
20.8
17.2
11.7
11.1
9.7
f1 (ppm)

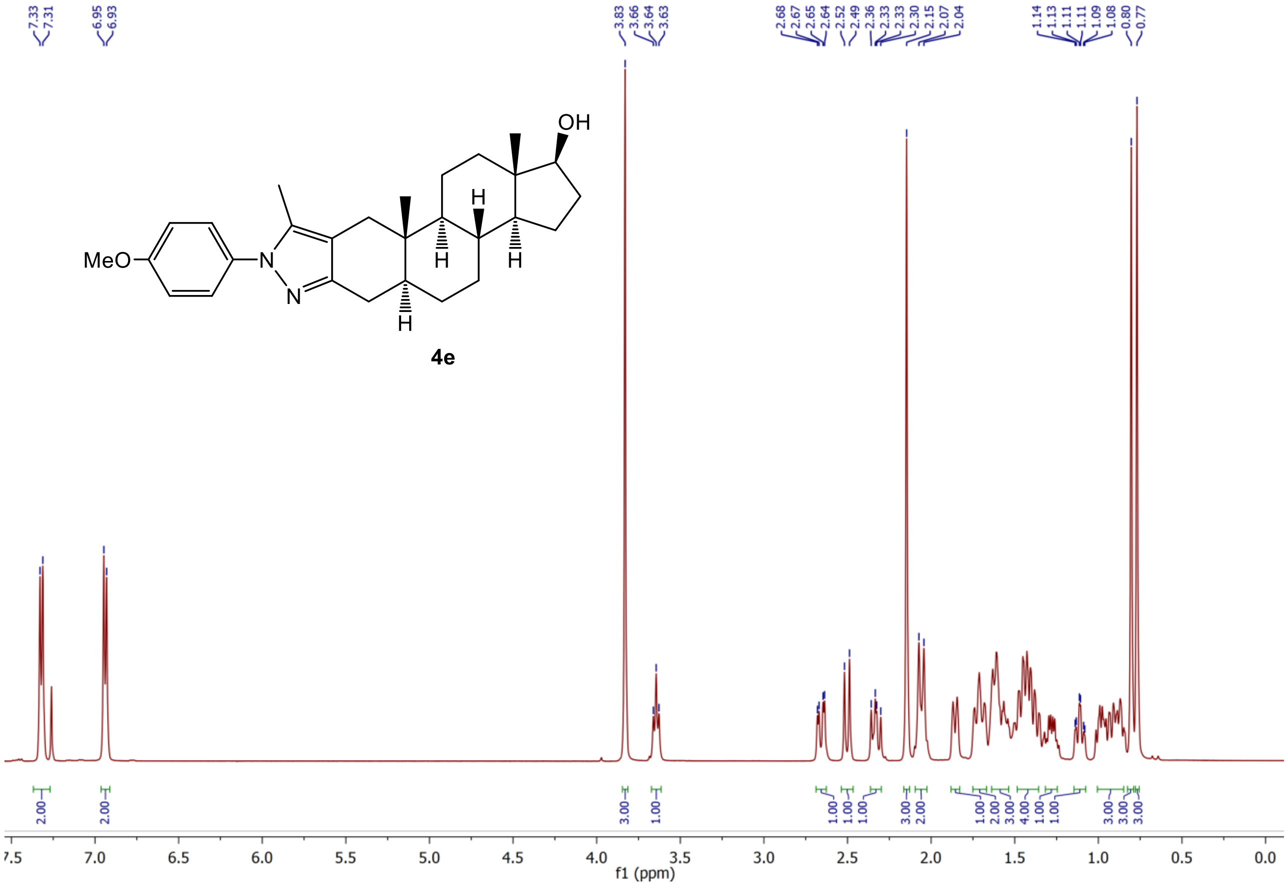

7.33
7.31
6.95
6.93
3.83
3.66
3.64
3.63
2.68
2.67
2.65
2.64
2.52
2.49
2.36
2.33
2.33
2.30
2.15
2.07
2.04
1.14
1.13
1.11
1.11
1.09
1.08
0.80
0.77
OH
H
MeO
N
4e
2.00
2.00
3.00
1.00
1.00
1.00
1.00
3.00
2.00
1.00
2.00
3.00
4.00
1.00
1.00
3.00
3.00
3.00
f1 (ppm)

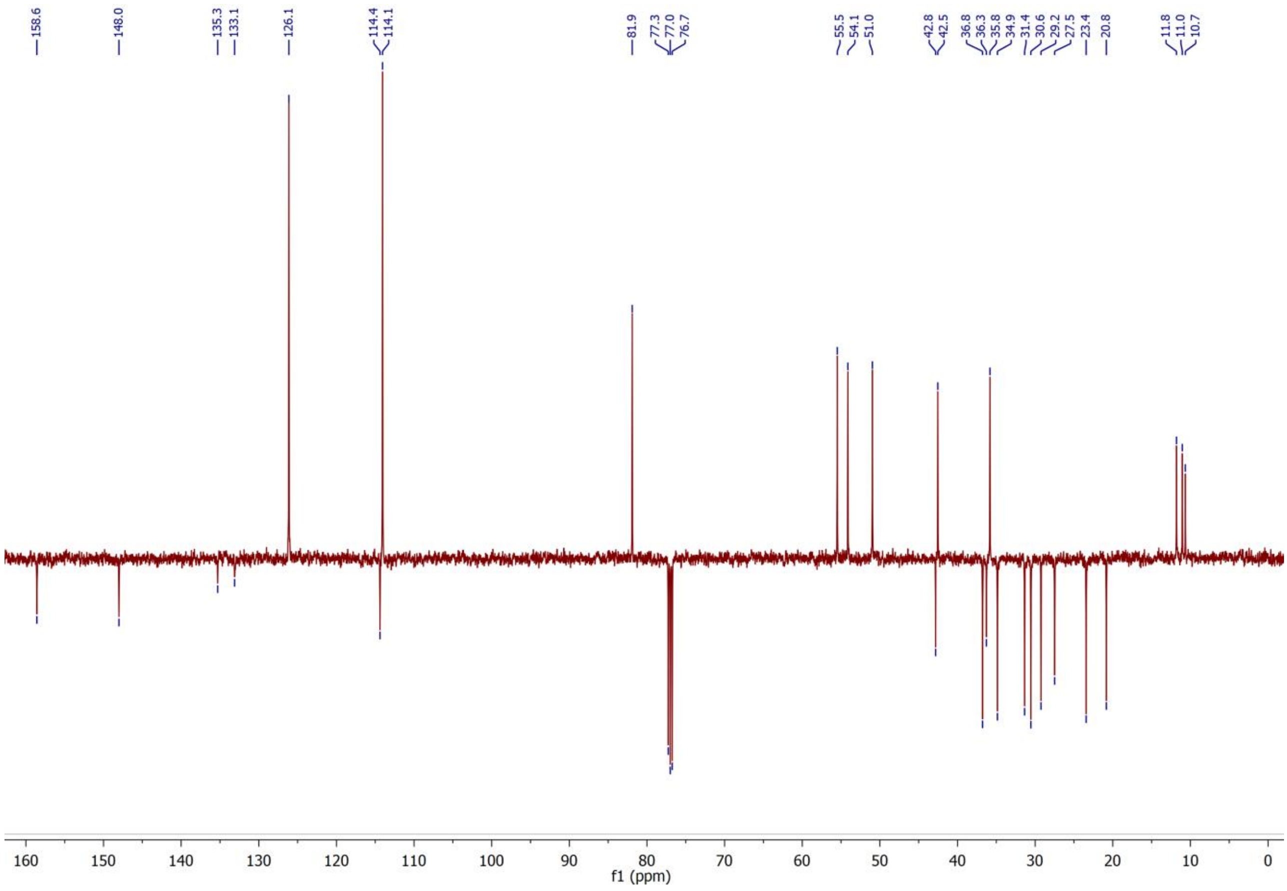

158.6
148.0
135.3
133.1
126.1
114.4
114.1
81.9
77.3
77.0
76.7
55.5
54.1
51.0
42.8
42.5
36.8
36.3
35.8
34.9
31.4
30.6
29.2
27.5
23.4
20.8
11.8
11.0
10.7
f1 (ppm)

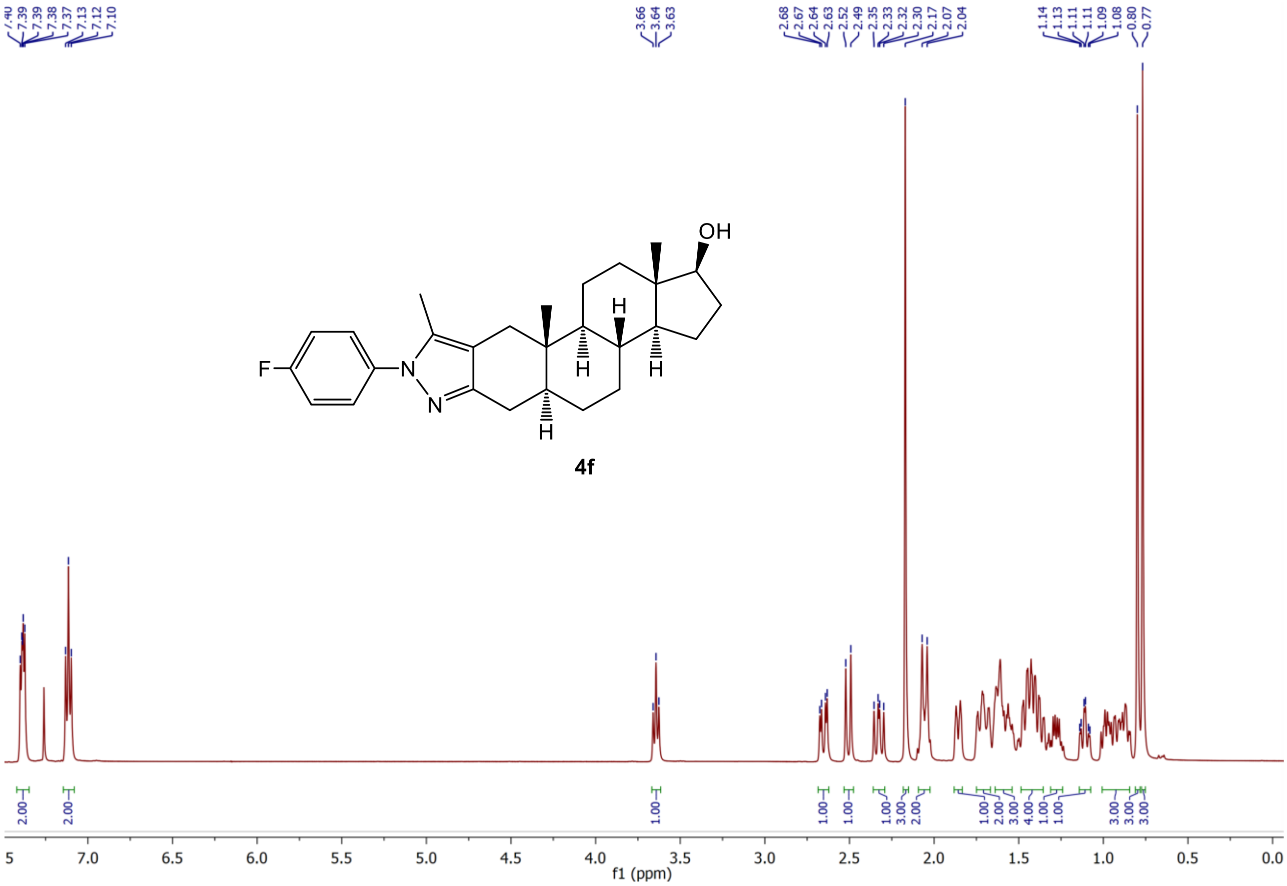
7.40 7.39 7.39 7.38 7.37 7.13 7.12 7.10
3.66 3.64 3.63
2.68 2.67 2.64 2.63 2.52 2.49 2.35 2.33 2.32 2.30 2.17 2.07 2.04
1.14 1.13 1.11 1.11 1.09 1.08 0.80 0.77
OH
H
H
H
F
N
N
H
4f
2.00 2.00 1.00 1.00 1.00 1.00 3.00 2.00 1.00 2.00 3.00 4.00 1.00 1.00 3.00 3.00 3.00
f1 (ppm)

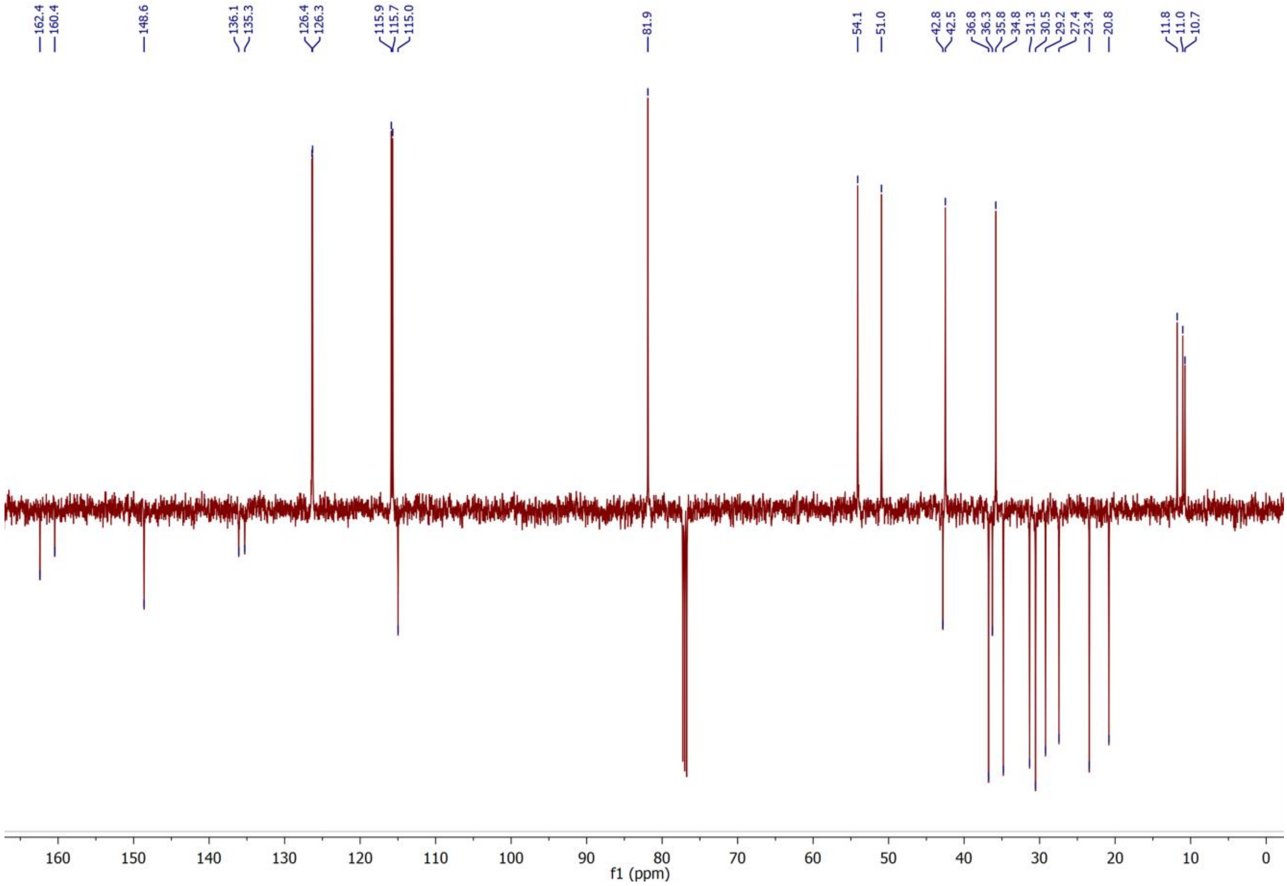
162.4 160.4 148.6 136.1 135.3 126.4 126.3 115.9 115.7 115.0 81.9 54.1 51.0 42.8 42.5 36.8 36.3 35.8 34.8 31.3 30.5 29.2 27.4 23.4 20.8 11.8 11.0 10.7
f1 (ppm)

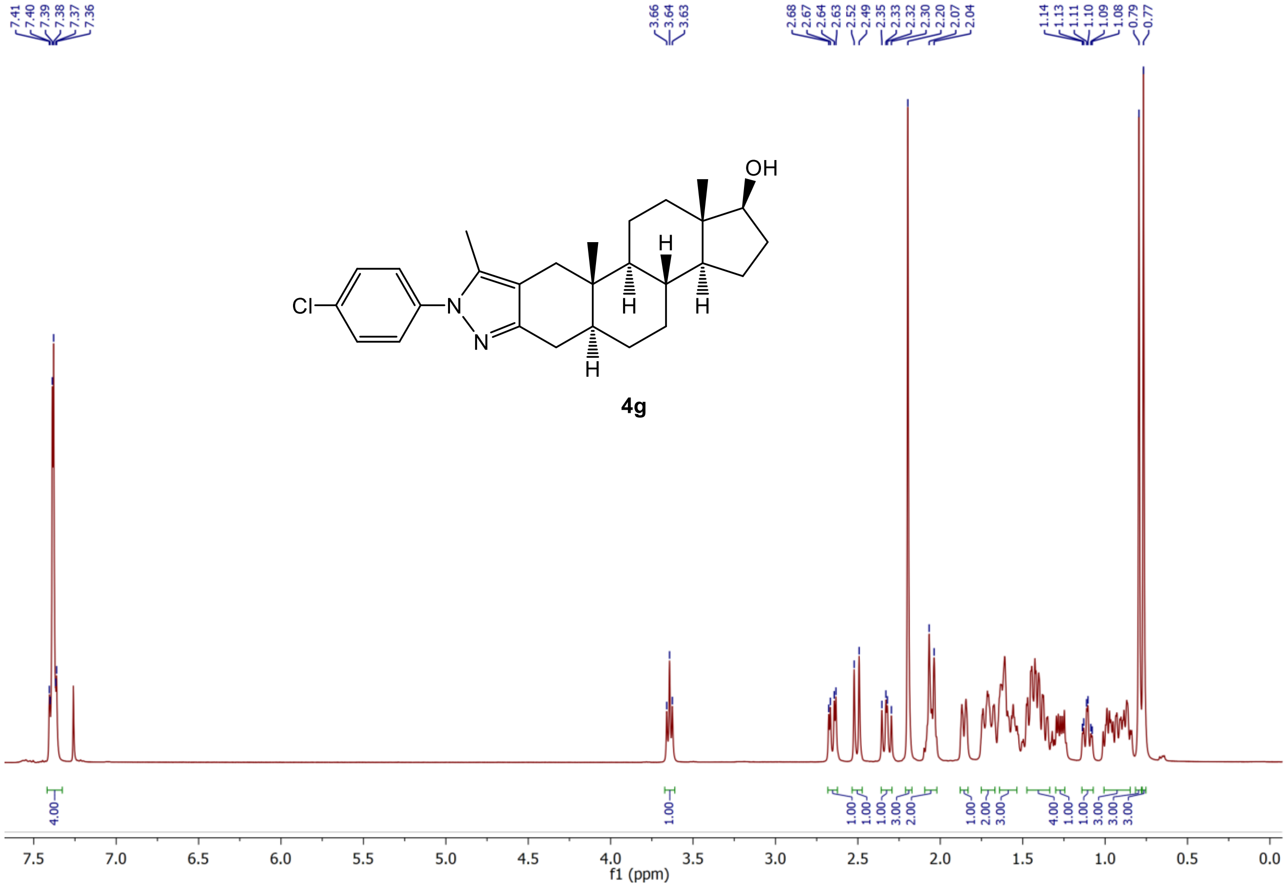
7.41
7.40
7.39
7.38
7.37
7.36
3.66
3.64
3.63
2.68
2.67
2.64
2.63
2.52
2.49
2.35
2.33
2.32
2.30
2.20
2.07
2.04
1.14
1.13
1.11
1.10
1.09
1.08
0.79
0.77
OH
H
H
H
H
Cl
N
N
4g
4.00
1.00
1.00
1.00
1.00
3.00
2.00
1.00
2.00
3.00
4.00
1.00
1.00
3.00
3.00
3.00
f1 (ppm)

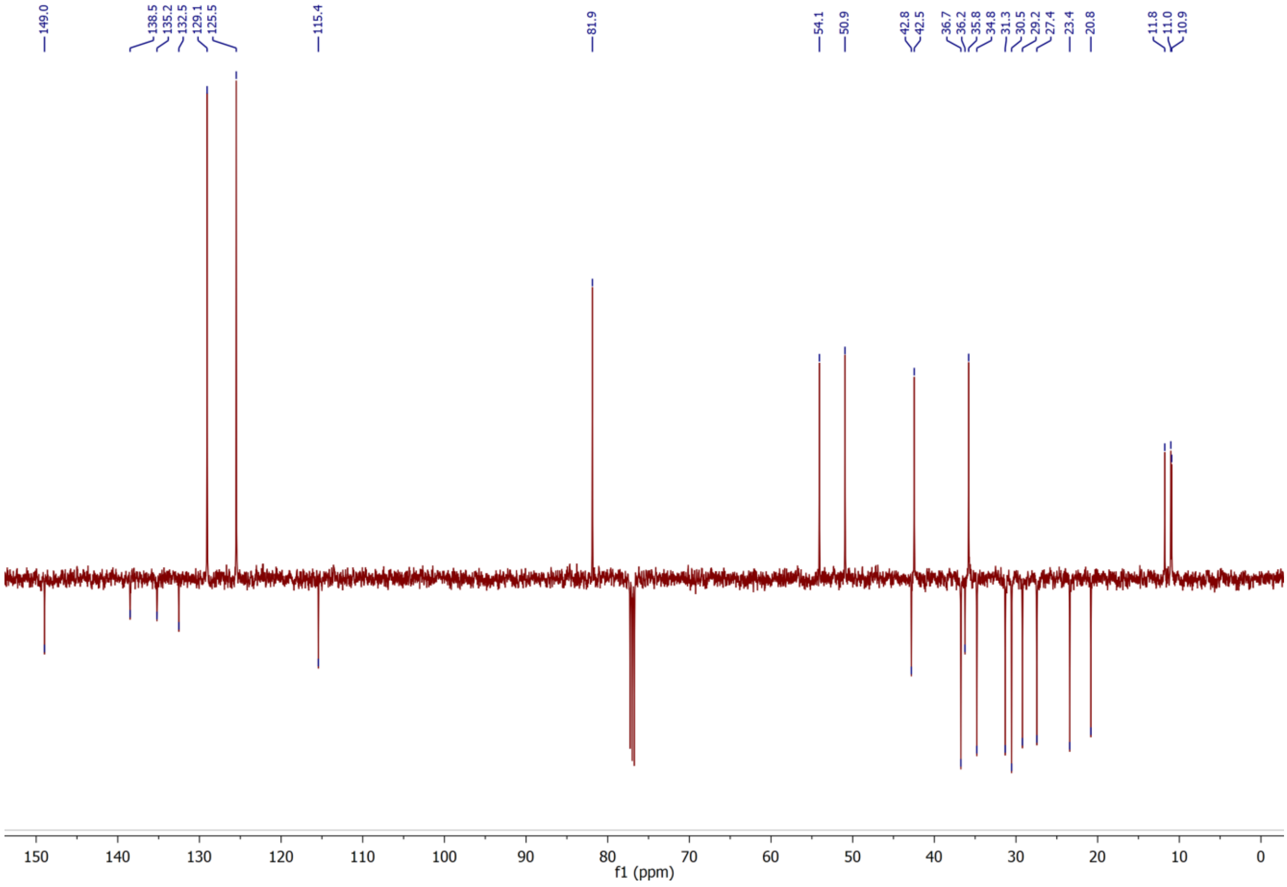
149.0
138.5
135.2
132.5
129.1
125.5
115.4
81.9
54.1
50.9
42.8
42.5
36.7
36.2
35.8
34.8
31.3
30.5
29.2
27.4
23.4
20.8
11.8
11.0
10.9
f1 (ppm)

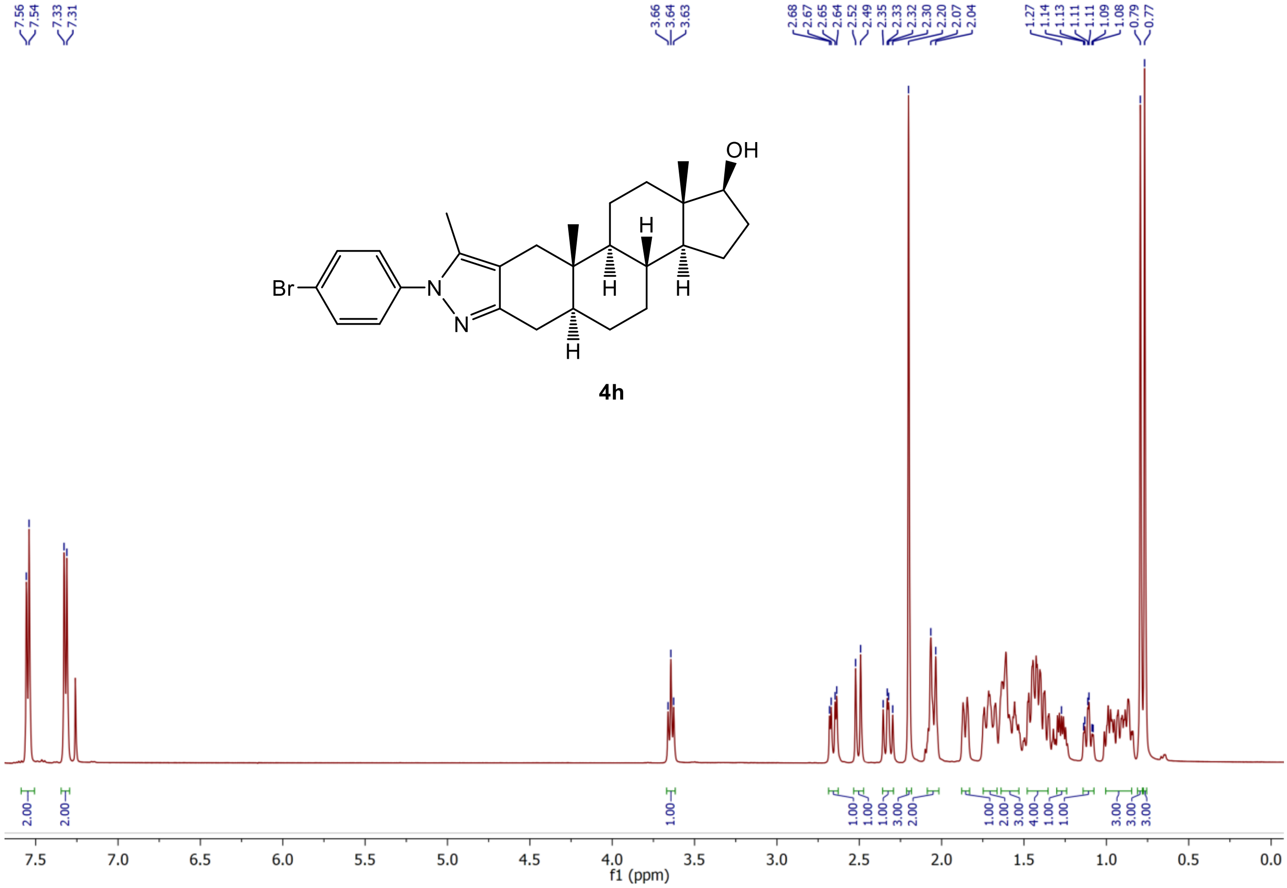
7.56
7.54
7.33
7.31
3.66
3.64
3.63
2.68
2.67
2.65
2.64
2.52
2.49
2.35
2.33
2.32
2.30
2.20
2.07
2.04
1.27
1.14
1.13
1.11
1.11
1.09
1.08
0.79
0.77
OH
H
H
H
Br
N
N
H
4h
2.00
2.00
1.00
1.00
1.00
1.00
3.00
2.00
1.00
2.00
3.00
4.00
1.00
1.00
3.00
3.00
3.00
7.5 7.0 6.5 6.0 5.5 5.0 4.5 4.0 3.5 3.0 2.5 2.0 1.5 1.0 0.5 0.0
f1 (ppm)

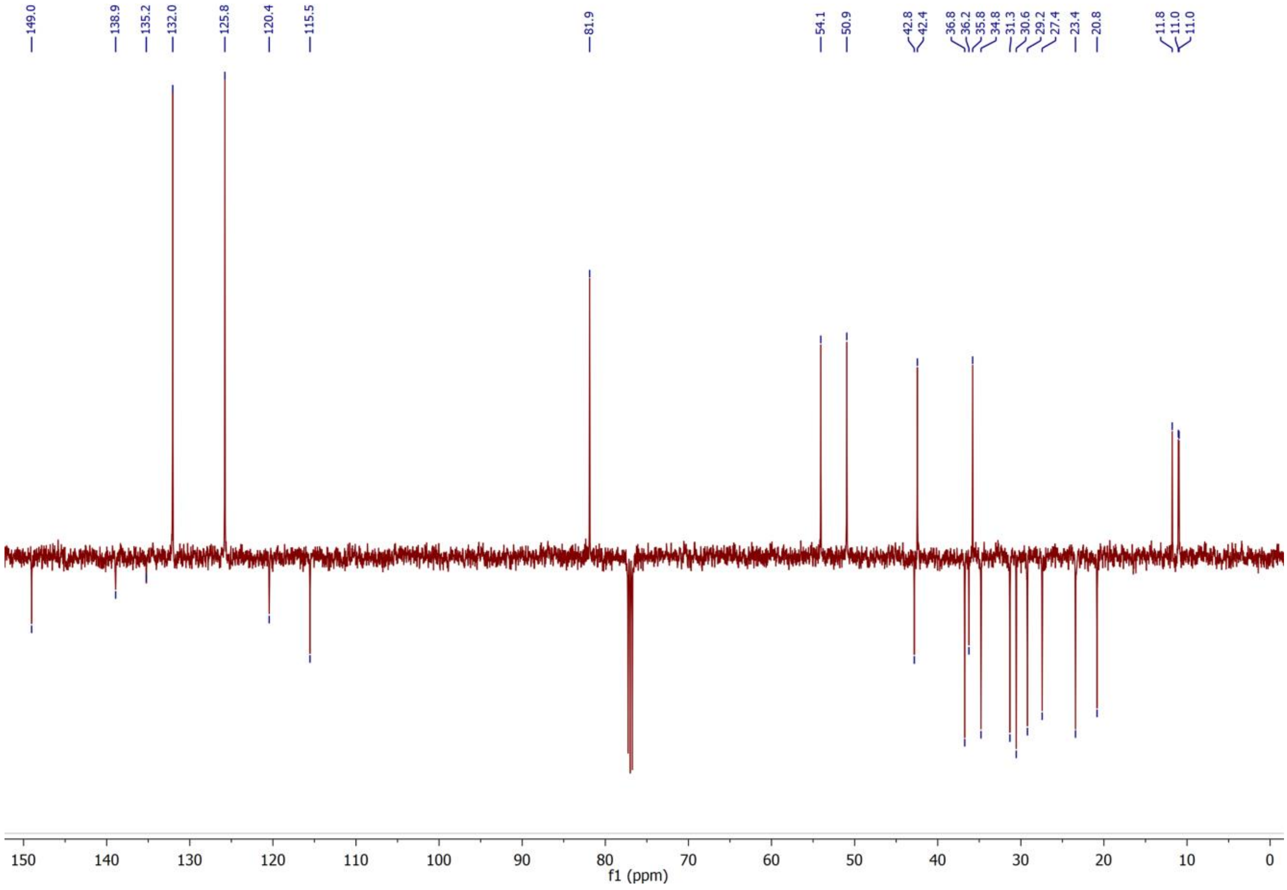
149.0
138.9
135.2
132.0
125.8
120.4
115.5
81.9
54.1
50.9
42.8
42.4
36.8
36.2
35.8
34.8
31.3
30.6
29.2
27.4
23.4
20.8
11.8
11.0
11.0
150 140 130 120 110 100 90 80 70 60 50 40 30 20 10 0
f1 (ppm)

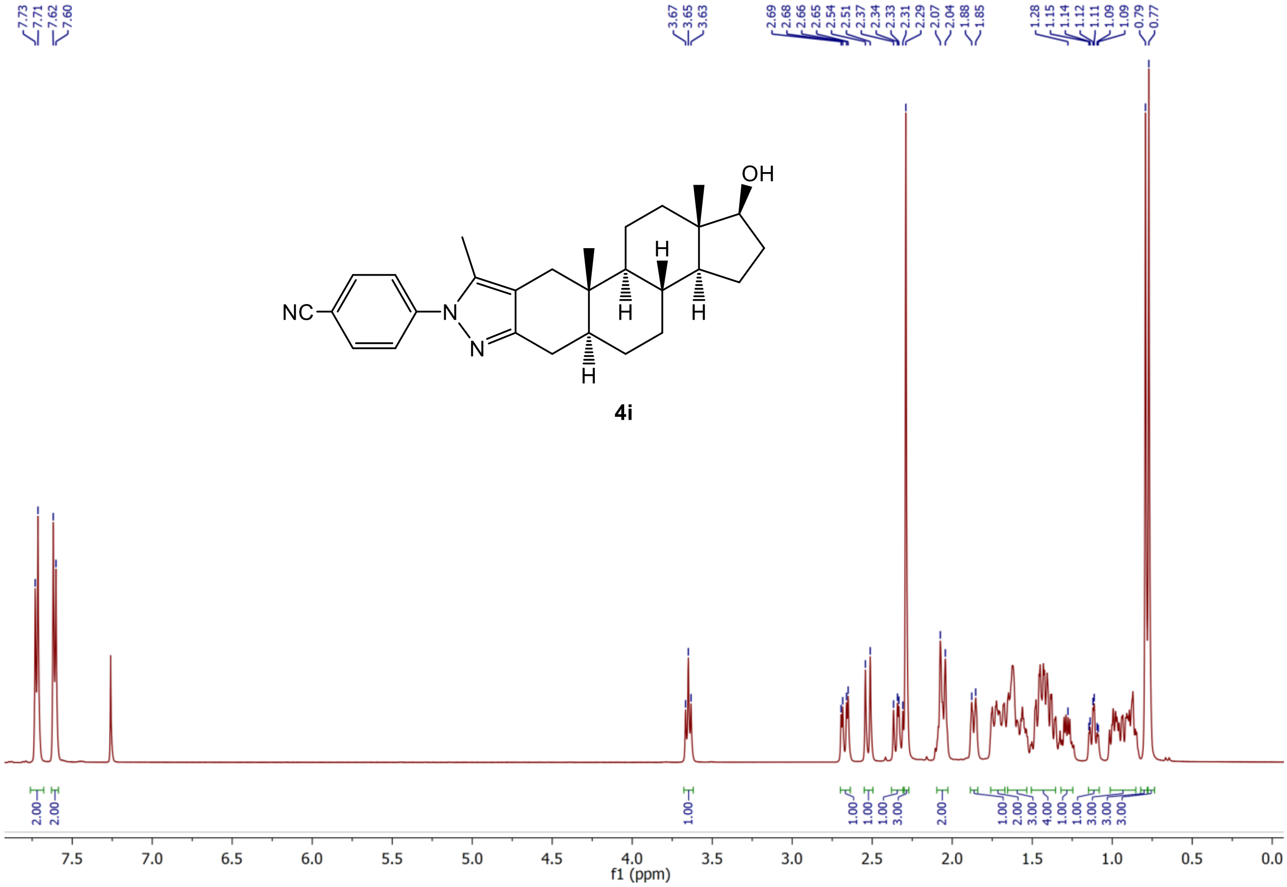
7.73
7.71
7.62
7.60
3.67
3.65
3.63
2.69
2.68
2.66
2.65
2.54
2.51
2.37
2.34
2.33
2.31
2.29
2.07
2.04
1.88
1.85
1.28
1.15
1.14
1.12
1.11
1.09
0.79
0.77
OH
H
NC
N
N
H
H
H
4i
2.00
2.00
1.00
1.00
1.00
1.00
3.00
2.00
1.00
2.00
3.00
4.00
1.00
1.00
3.00
3.00
3.00
f1 (ppm)

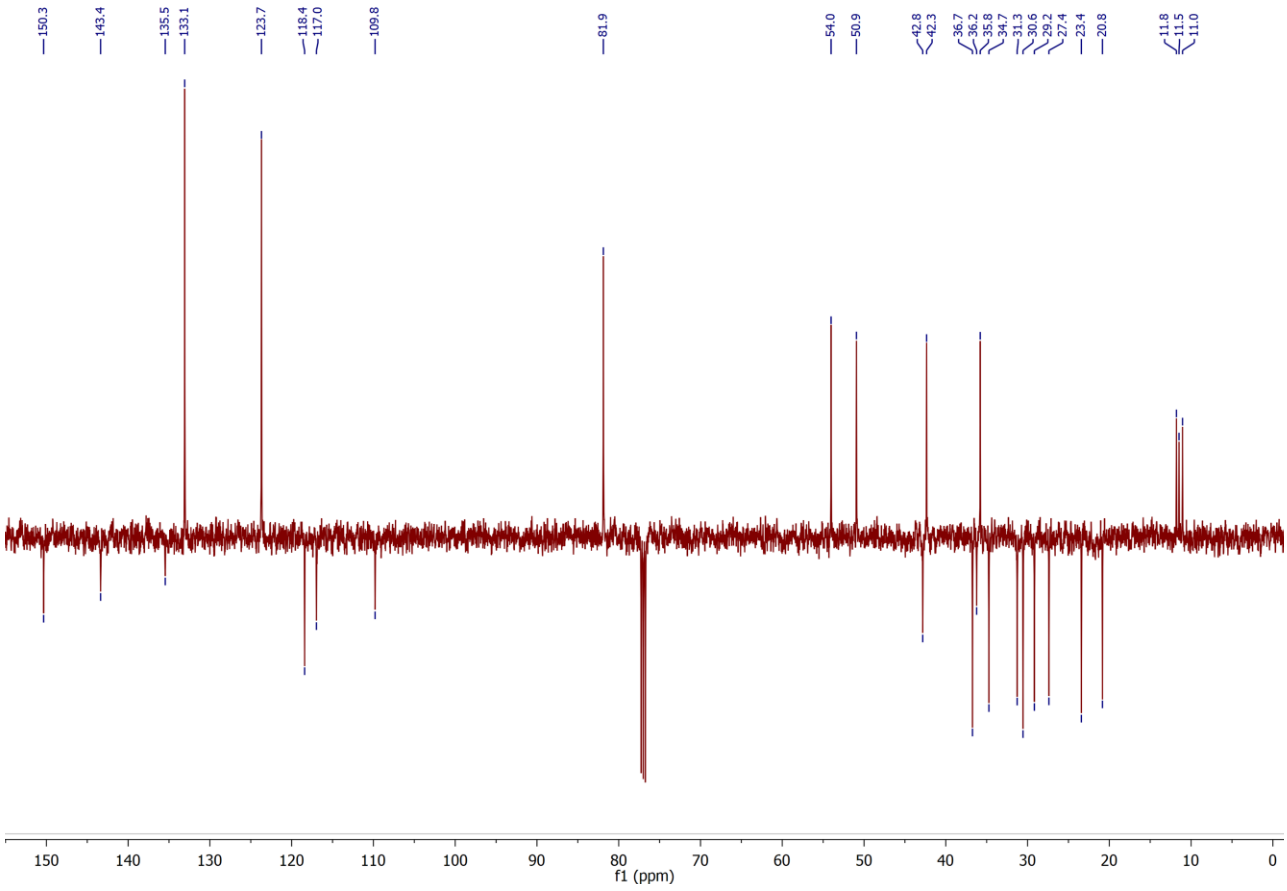
150.3
143.4
135.5
133.1
123.7
118.4
117.0
109.8
81.9
54.0
50.9
42.8
42.3
36.7
36.2
35.8
34.7
31.3
30.6
29.2
27.4
23.4
20.8
11.8
11.5
11.0
f1 (ppm)

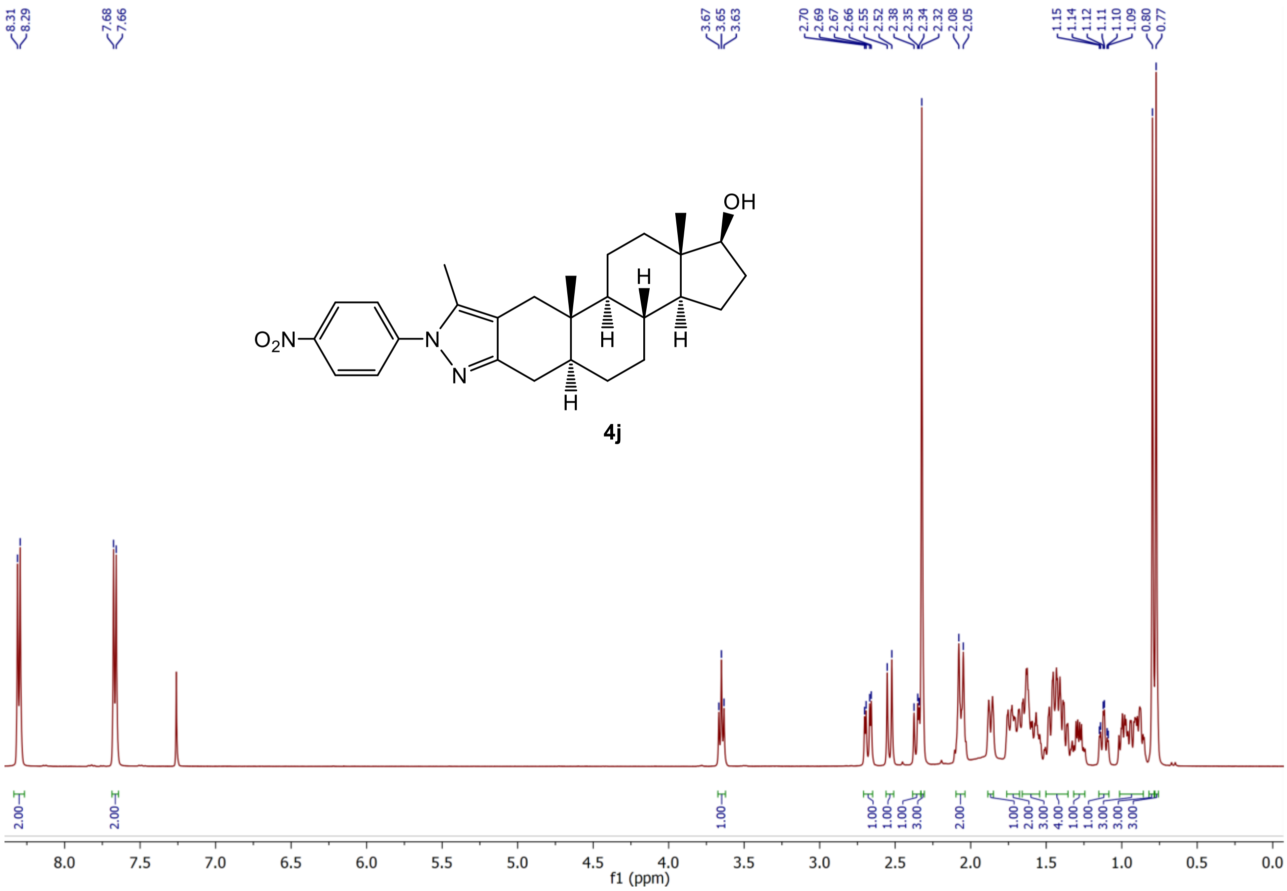
8.31
8.29
7.68
7.66
3.67
3.65
3.63
2.70
2.69
2.67
2.66
2.55
2.52
2.38
2.35
2.34
2.32
2.08
2.05
1.15
1.14
1.12
1.11
1.10
1.09
0.80
0.77
OH
H
H
H
H
O2N
N
N
4j
f1 (ppm)

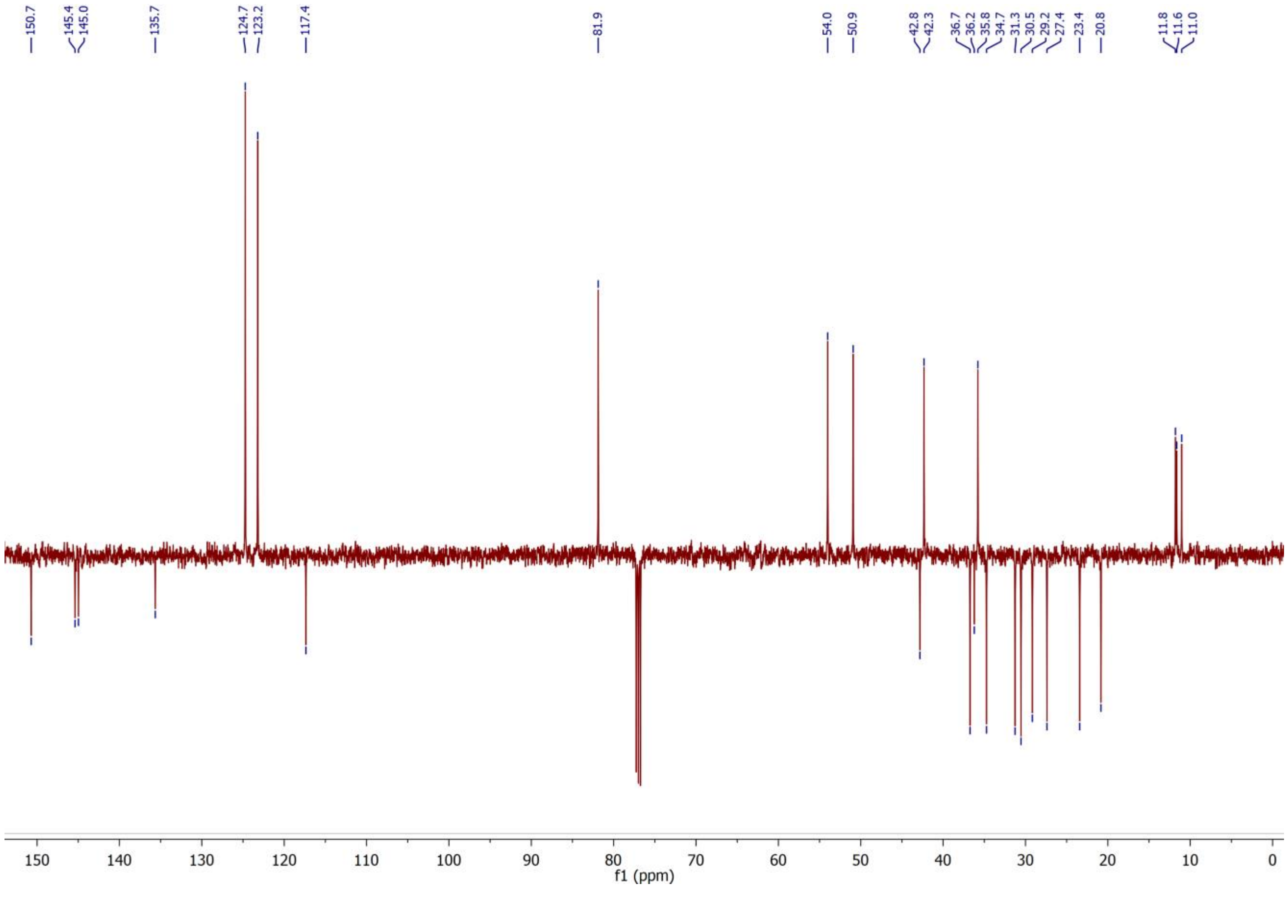
150.7
145.4
145.0
135.7
124.7
123.2
117.4
81.9
54.0
50.9
42.8
42.3
36.7
36.2
35.8
34.7
31.3
30.5
29.2
27.4
23.4
20.8
11.8
11.6
11.0
f1 (ppm)

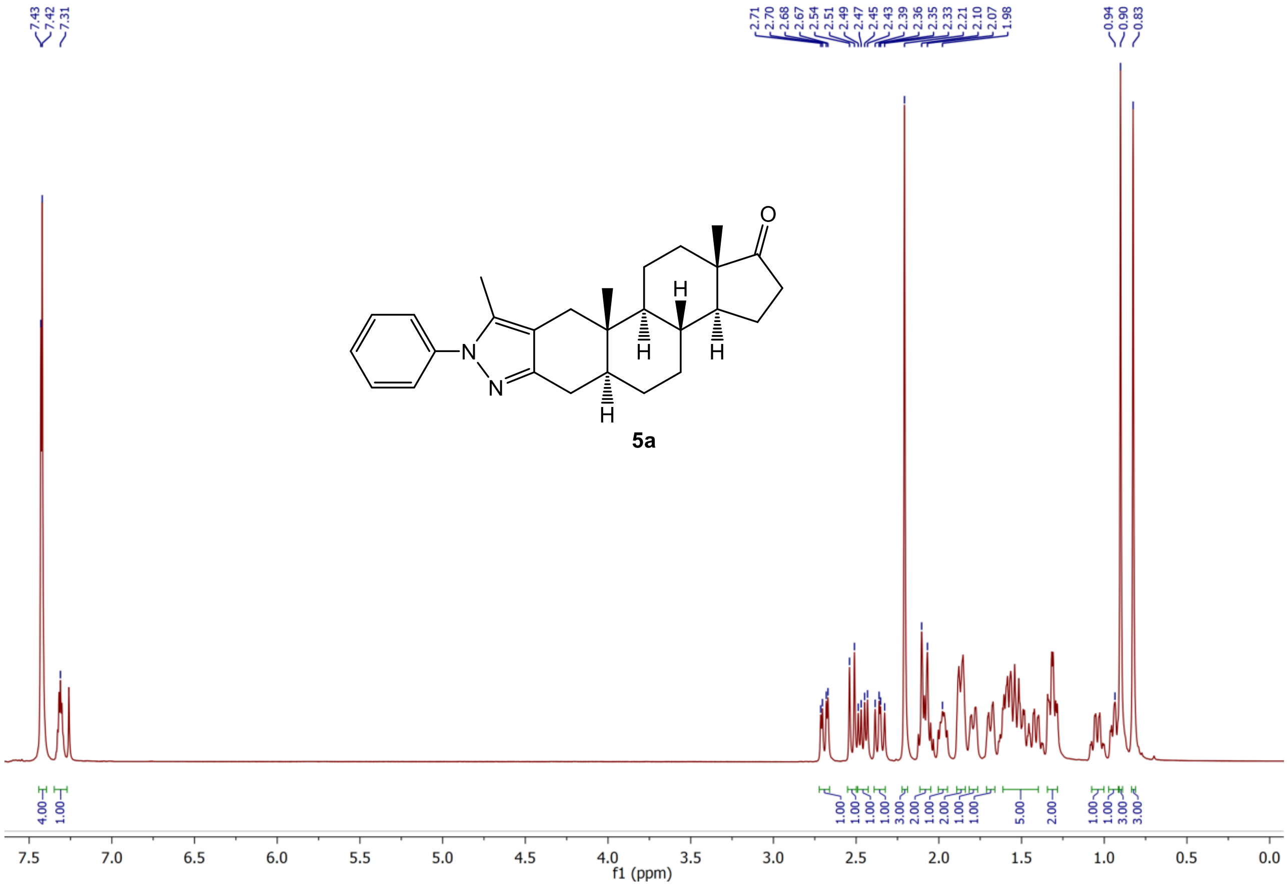
7.43
7.42
7.31
2.71
2.70
2.68
2.67
2.54
2.51
2.49
2.47
2.45
2.43
2.39
2.36
2.35
2.33
2.21
2.10
2.07
1.98
0.94
0.90
0.83
O
H
N
N
5a
4.00
1.00
1.00
1.00
1.00
1.00
3.00
2.00
1.00
2.00
1.00
1.00
5.00
2.00
1.00
1.00
3.00
3.00
f1 (ppm)

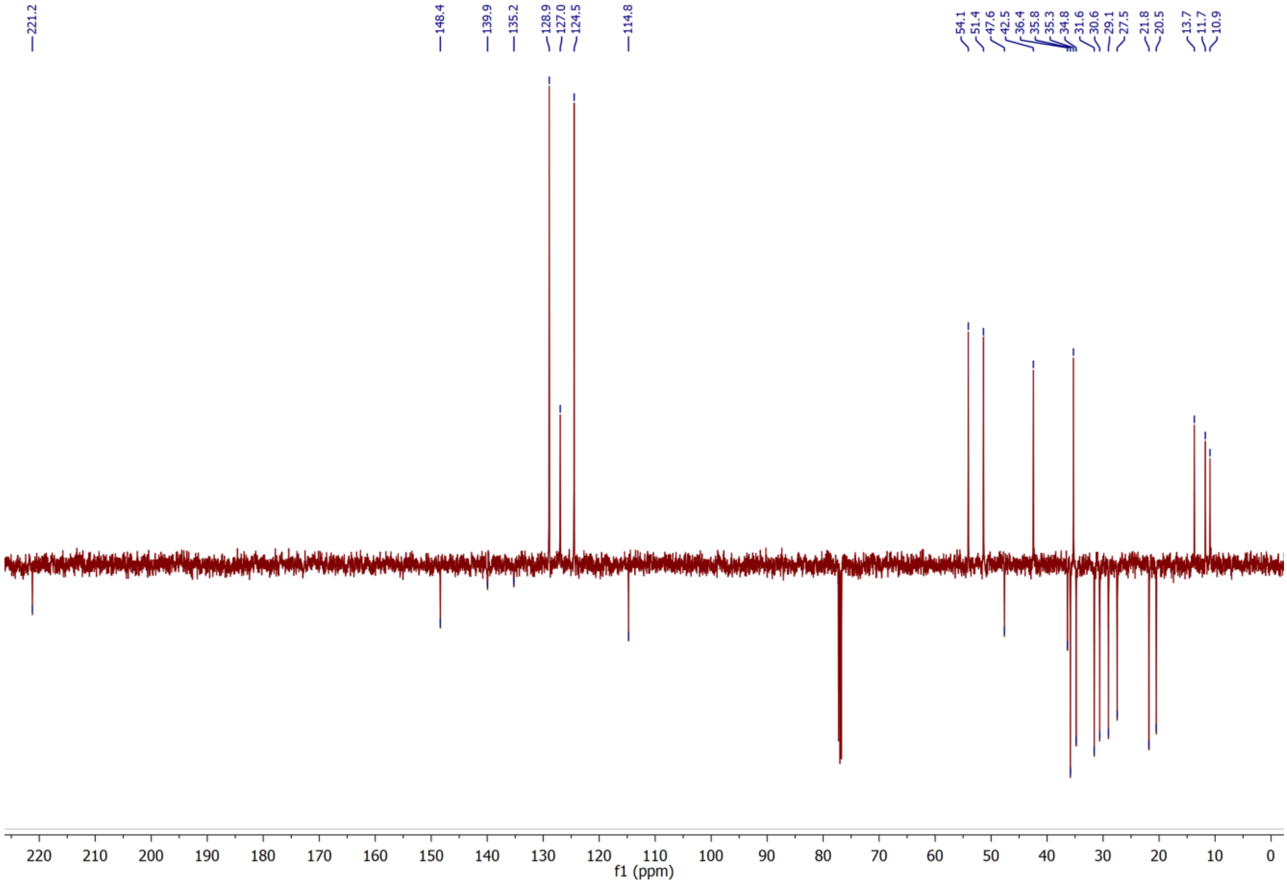
221.2
148.4
139.9
135.2
128.9
127.0
124.5
114.8
54.1
51.4
47.6
42.5
36.4
35.8
35.3
34.8
31.6
30.6
29.1
27.5
21.8
20.5
13.7
11.7
10.9
f1 (ppm)

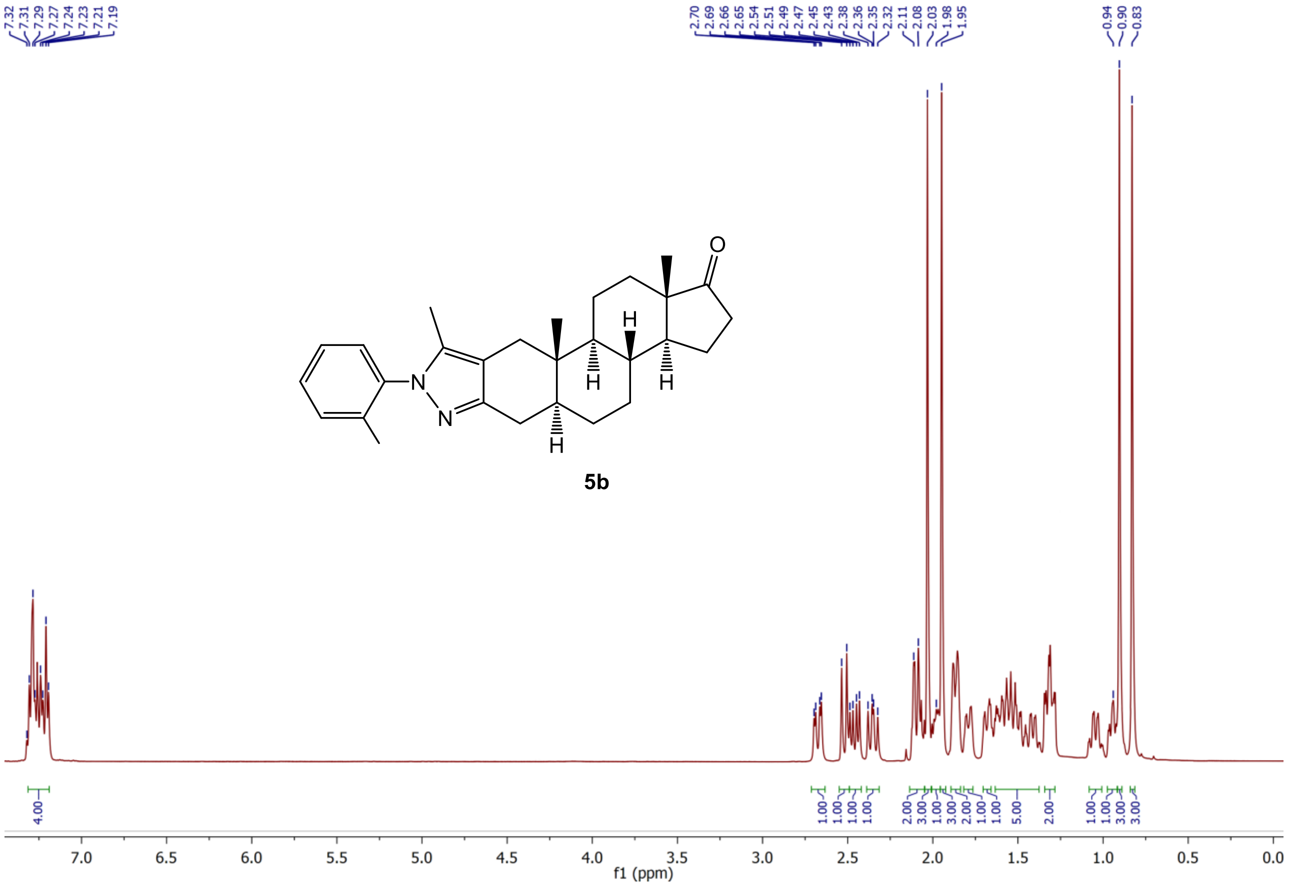
7.32
7.31
7.29
7.27
7.24
7.23
7.21
7.19
2.70
2.69
2.66
2.65
2.54
2.51
2.49
2.47
2.45
2.43
2.38
2.36
2.35
2.32
2.11
2.08
2.03
1.98
1.95
0.94
0.90
0.83
5b
4.00
1.00
1.00
1.00
1.00
2.00
3.00
1.00
3.00
2.00
1.00
1.00
5.00
2.00
1.00
1.00
3.00
3.00
f1 (ppm)

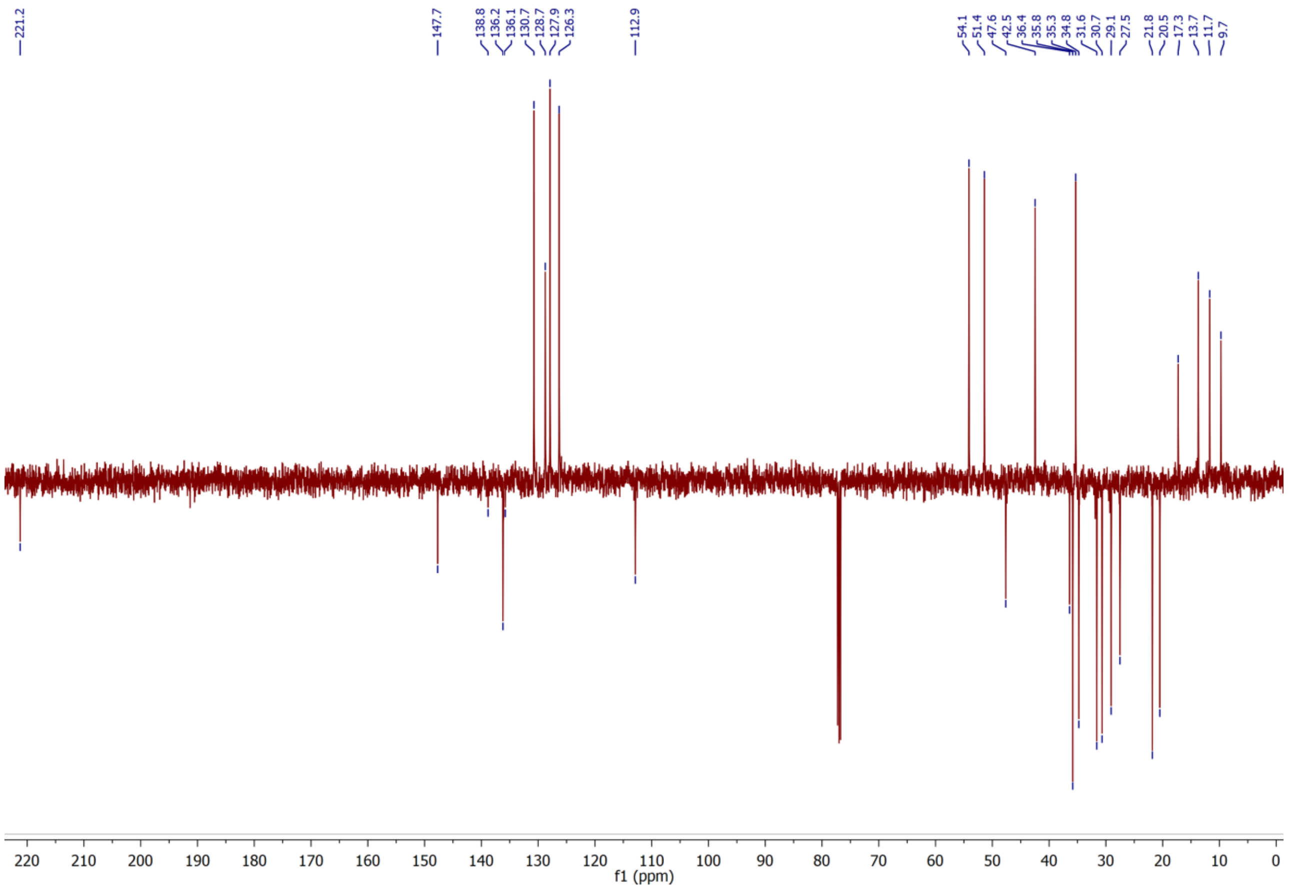
221.2
147.7
138.8
136.2
136.1
130.7
128.7
127.9
126.3
112.9
54.1
51.4
47.6
42.5
36.4
35.8
35.3
34.8
31.6
30.7
29.1
27.5
21.8
20.5
17.3
13.7
11.7
9.7
f1 (ppm)

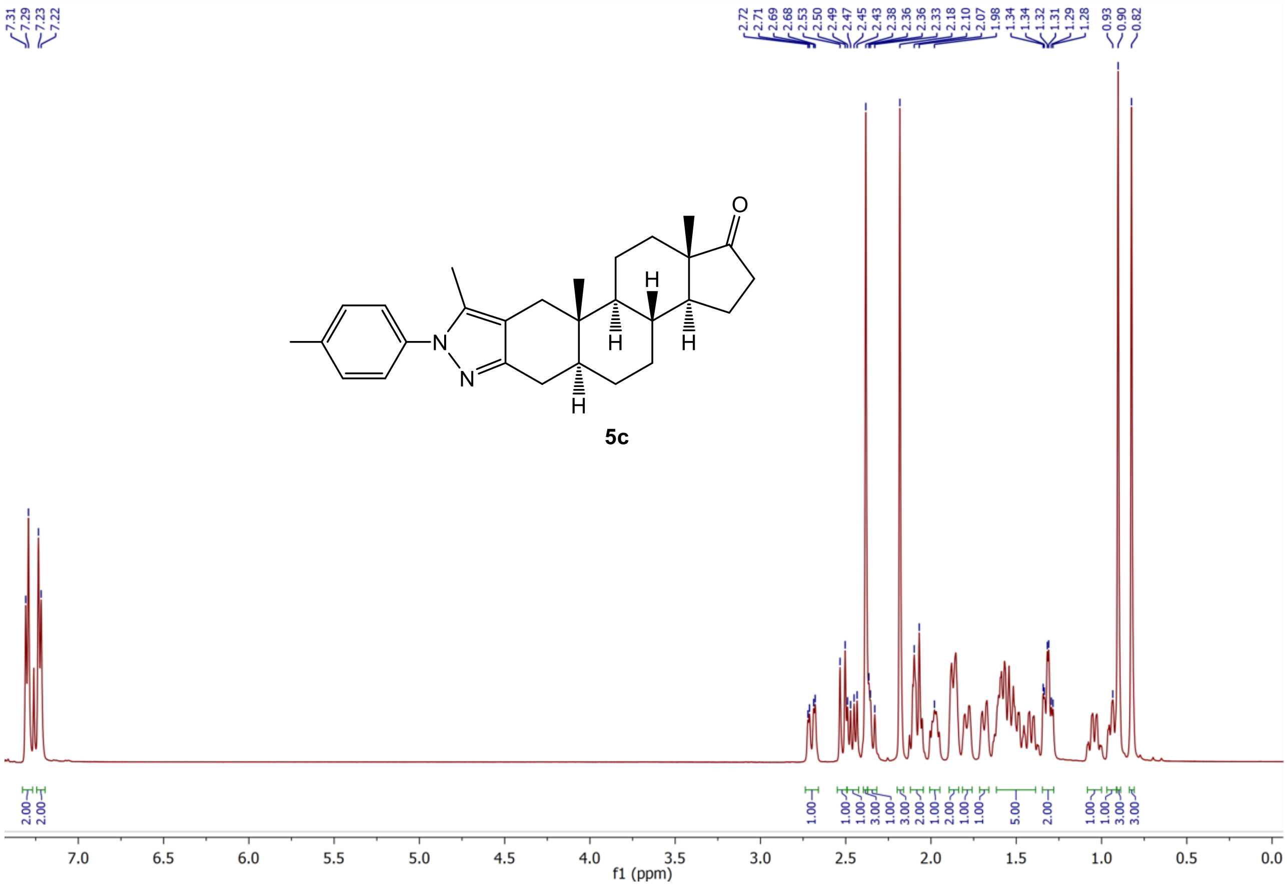
7.31
7.29
7.23
7.22
2.72
2.71
2.69
2.68
2.53
2.50
2.49
2.47
2.45
2.43
2.38
2.36
2.36
2.33
2.18
2.10
2.07
1.98
1.34
1.34
1.32
1.31
1.29
1.28
0.93
0.90
0.82
O
H
H
H
H
N
N
5c
2.00
2.00
1.00
1.00
1.00
3.00
1.00
3.00
2.00
1.00
2.00
1.00
1.00
5.00
2.00
1.00
1.00
3.00
3.00
7.0
6.5
6.0
5.5
5.0
4.5
4.0
3.5
3.0
2.5
2.0
1.5
1.0
0.5
0.0
f1 (ppm)

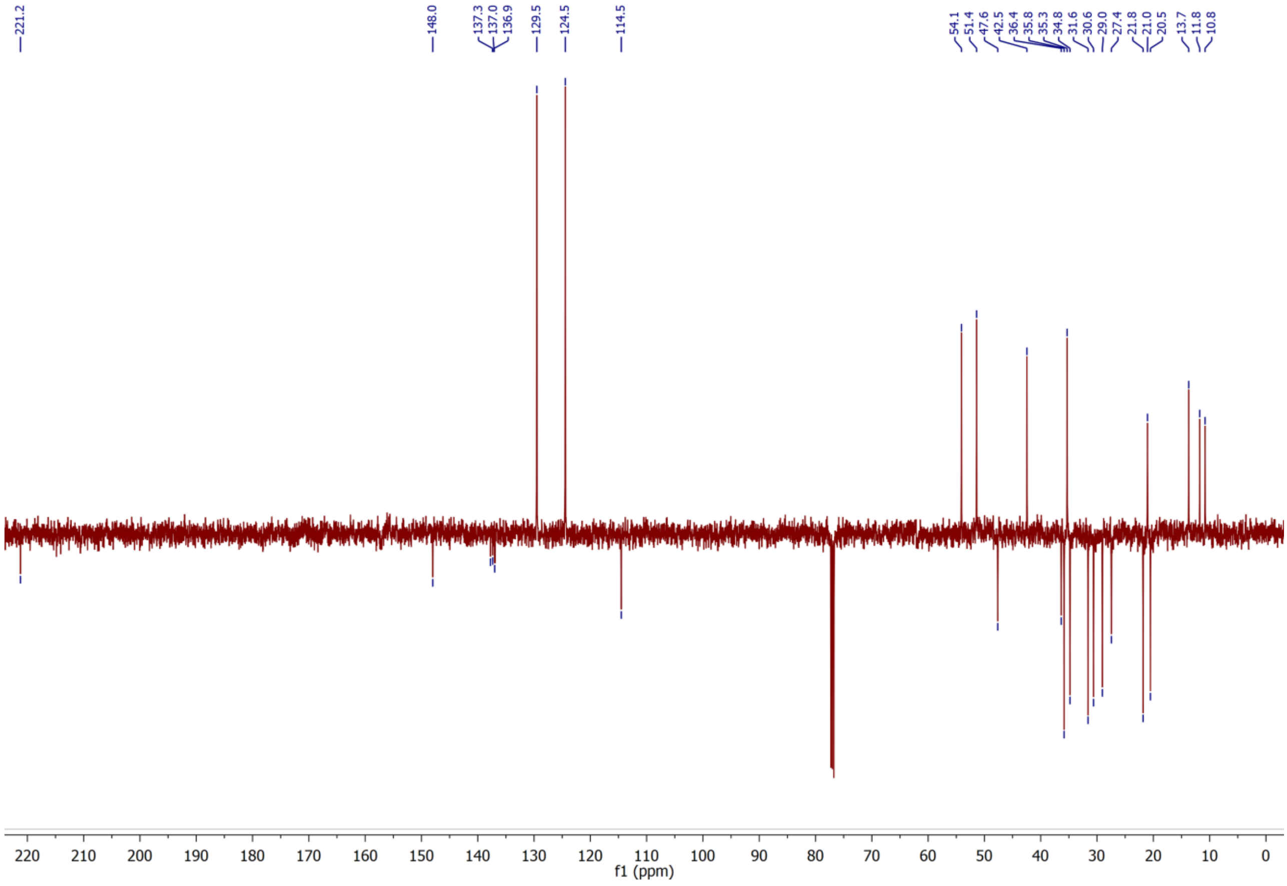
221.2
148.0
137.3
137.0
136.9
129.5
124.5
114.5
54.1
51.4
47.6
42.5
36.4
35.8
35.3
34.8
31.6
30.6
29.0
27.4
21.8
21.0
20.5
13.7
11.8
10.8
220
210
200
190
180
170
160
150
140
130
120
110
100
90
80
70
60
50
40
30
20
10
0
f1 (ppm)

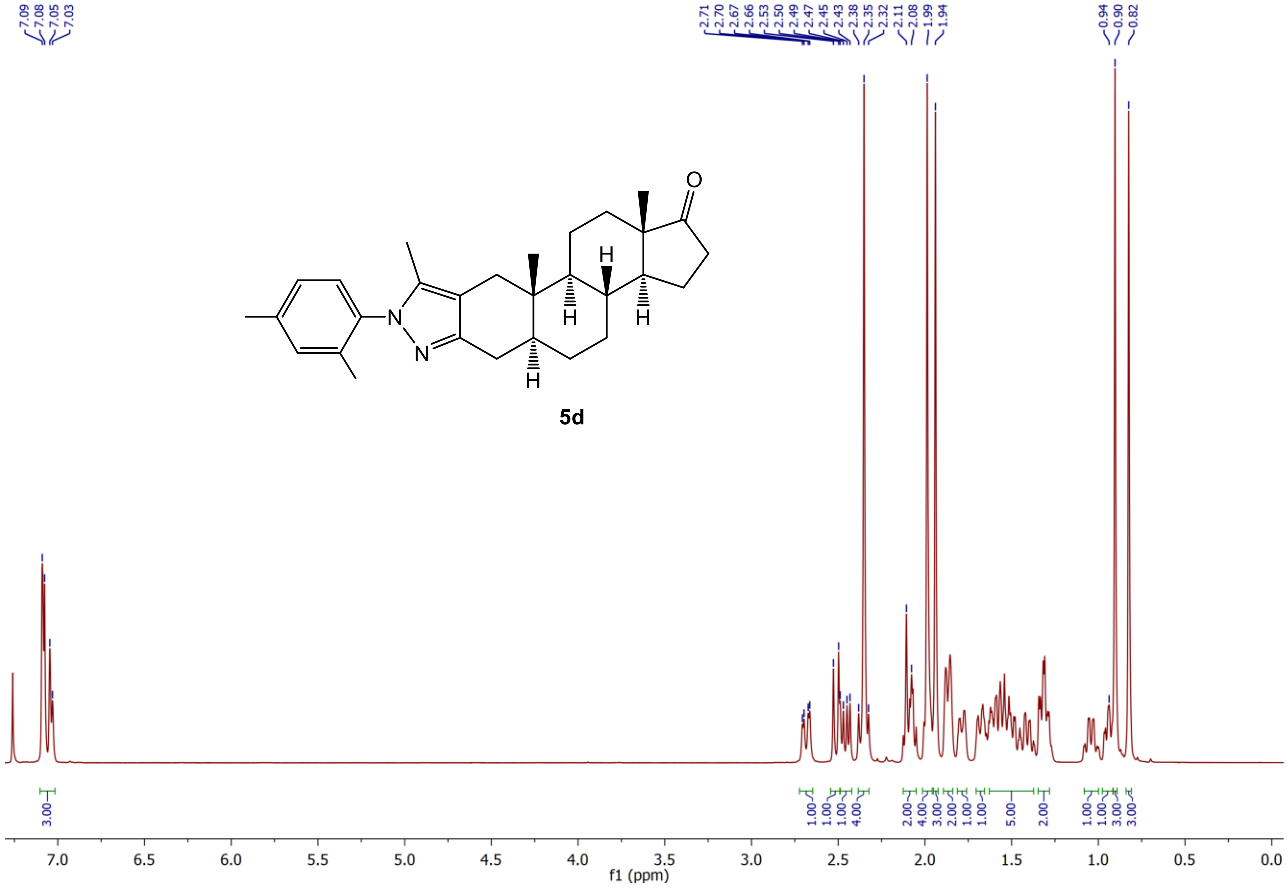
7.09
7.08
7.05
7.03
2.71
2.70
2.67
2.66
2.53
2.50
2.49
2.47
2.45
2.43
2.38
2.35
2.32
2.11
2.08
1.99
1.94
0.94
0.90
0.82
O
H
H
H
H
N
N
5d
3.00
1.00
1.00
1.00
4.00
2.00
4.00
3.00
2.00
1.00
1.00
5.00
2.00
1.00
1.00
3.00
3.00
7.0
6.5
6.0
5.5
5.0
4.5
4.0
3.5
3.0
2.5
2.0
1.5
1.0
0.5
0.0
f1 (ppm)

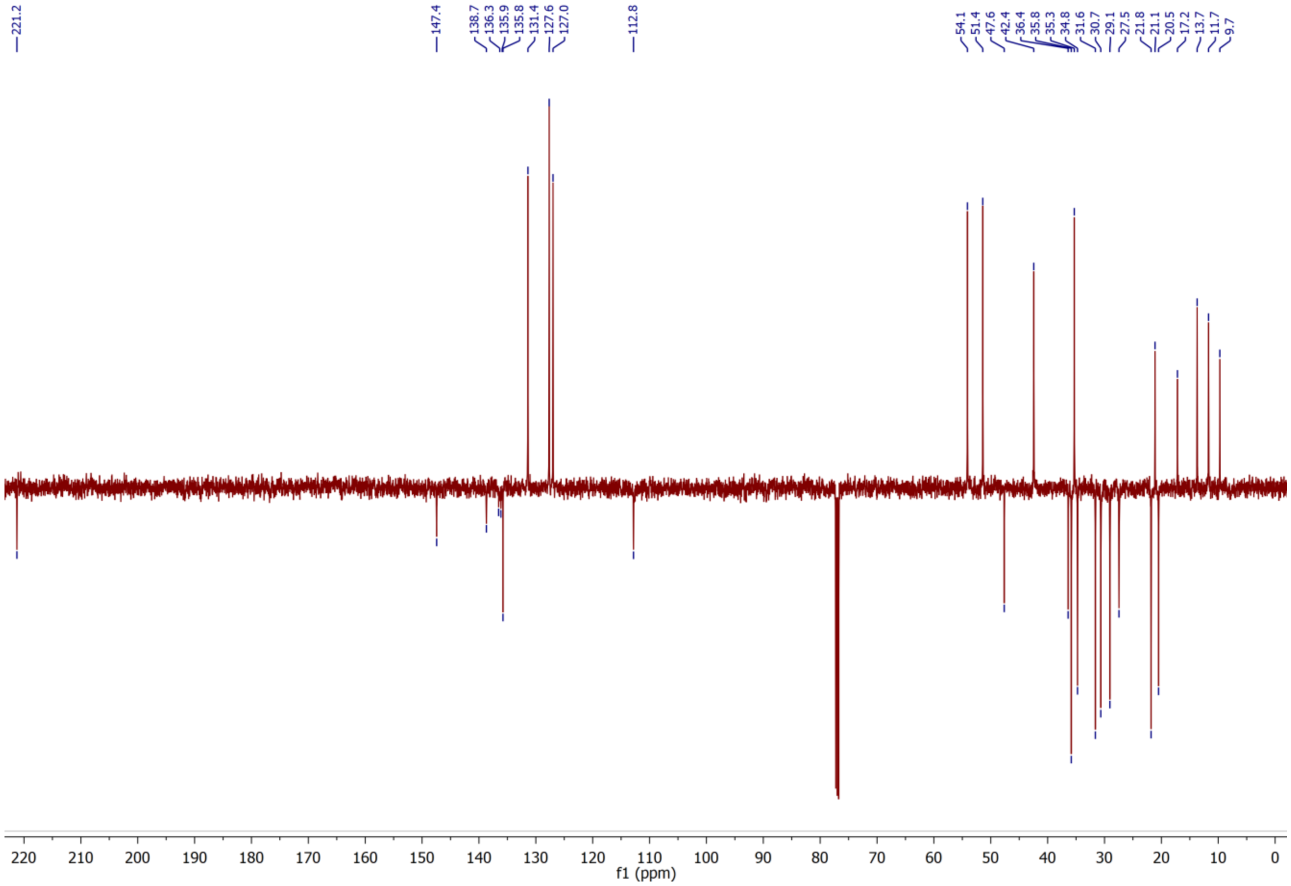
221.2
147.4
138.7
136.3
135.9
135.8
131.4
127.6
127.0
112.8
54.1
51.4
47.6
42.4
36.4
35.8
35.3
34.8
31.6
30.7
29.1
27.5
21.8
21.1
20.5
17.2
13.7
11.7
9.7
220
210
200
190
180
170
160
150
140
130
120
110
100
90
80
70
60
50
40
30
20
10
0
f1 (ppm)

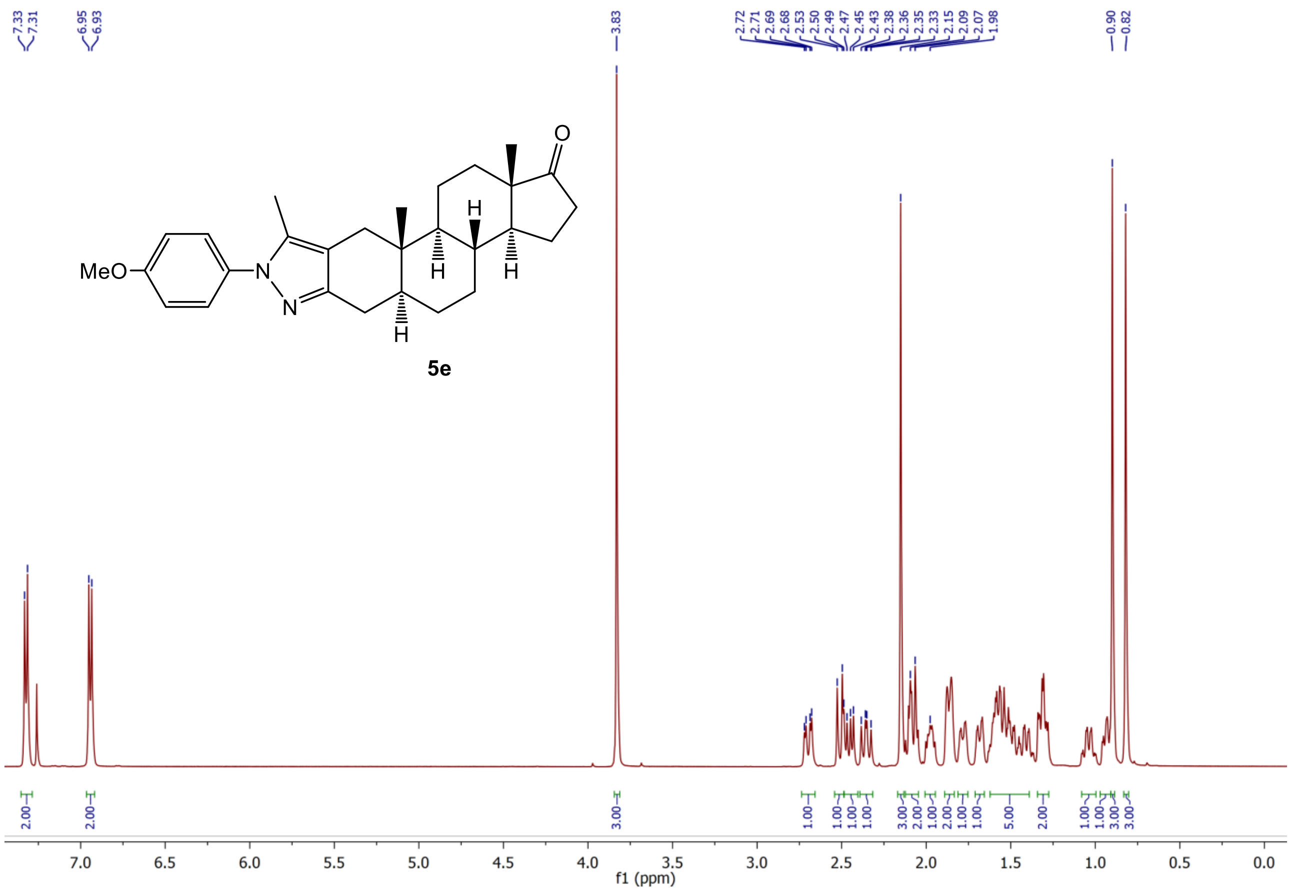
7.33
7.31
6.95
6.93
3.83
2.72
2.71
2.69
2.68
2.53
2.50
2.49
2.47
2.45
2.43
2.38
2.36
2.35
2.33
2.15
2.09
2.07
1.98
0.90
0.82
MeO
N
N
H
H
H
H
O
5e
2.00
2.00
3.00
1.00
1.00
1.00
1.00
3.00
2.00
1.00
2.00
1.00
1.00
5.00
2.00
1.00
1.00
3.00
3.00
f1 (ppm)

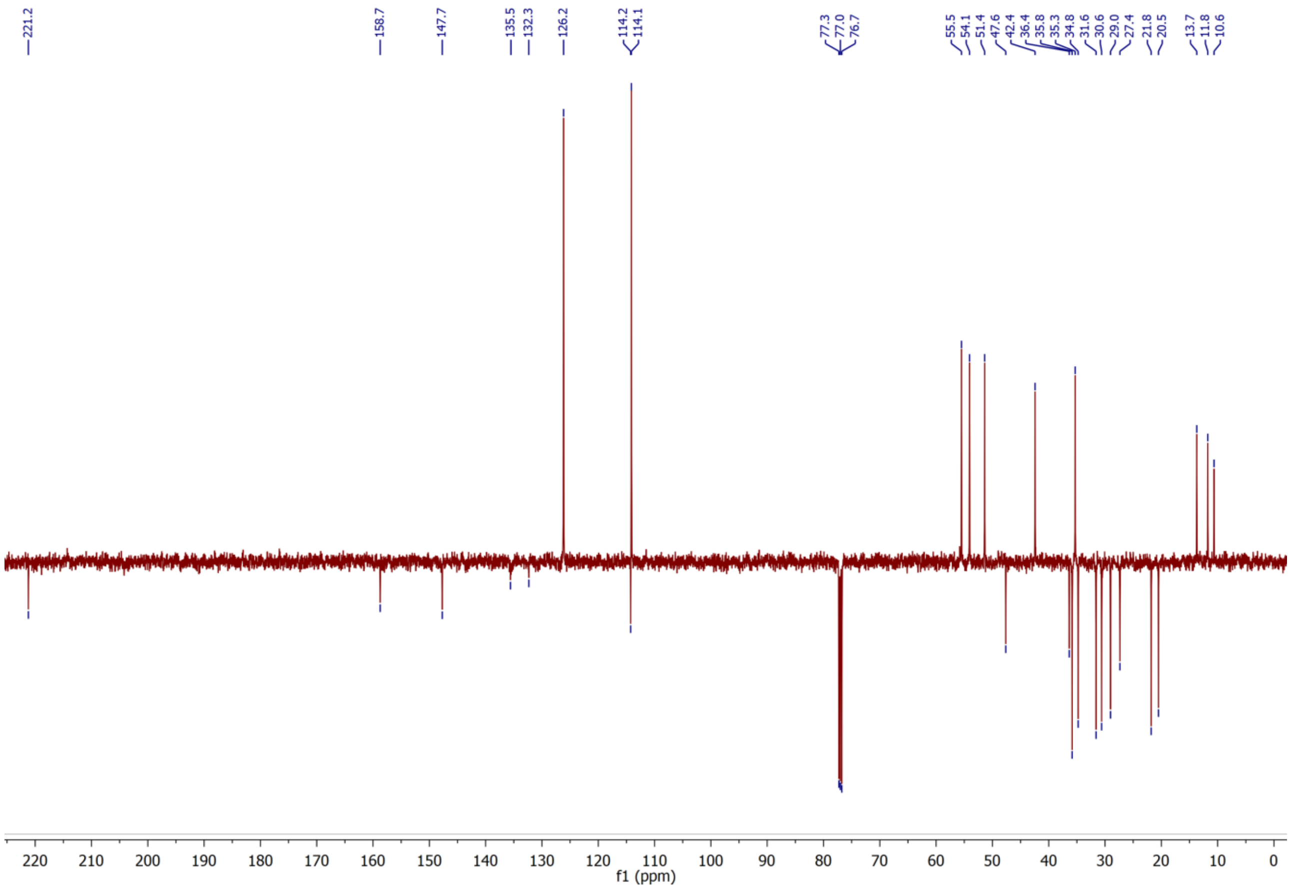
221.2
158.7
147.7
135.5
132.3
126.2
114.2
114.1
77.3
77.0
76.7
55.5
54.1
51.4
47.6
42.4
36.4
35.8
35.3
34.8
31.6
30.6
29.0
27.4
21.8
20.5
13.7
11.8
10.6
f1 (ppm)

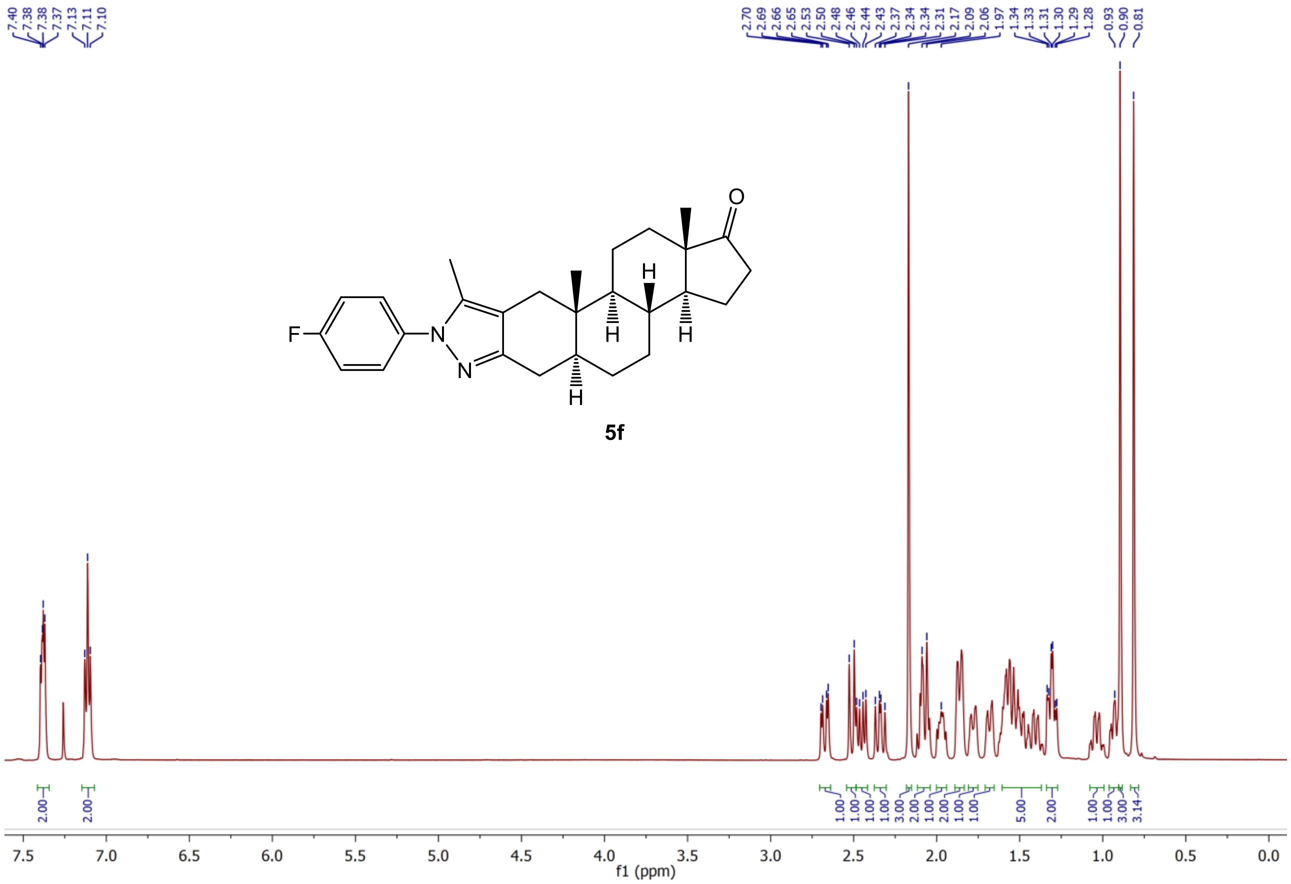
7.40
7.38
7.38
7.37
7.13
7.11
7.10
2.70
2.69
2.66
2.65
2.53
2.50
2.48
2.46
2.44
2.43
2.37
2.34
2.34
2.31
2.17
2.09
2.06
1.97
1.34
1.33
1.31
1.30
1.29
1.28
0.93
0.90
0.81
F
N
N
O
H
H
H
H
5f
2.00
2.00
1.00
1.00
1.00
1.00
3.00
2.00
1.00
2.00
1.00
1.00
5.00
2.00
1.00
1.00
3.00
3.14
f1 (ppm)

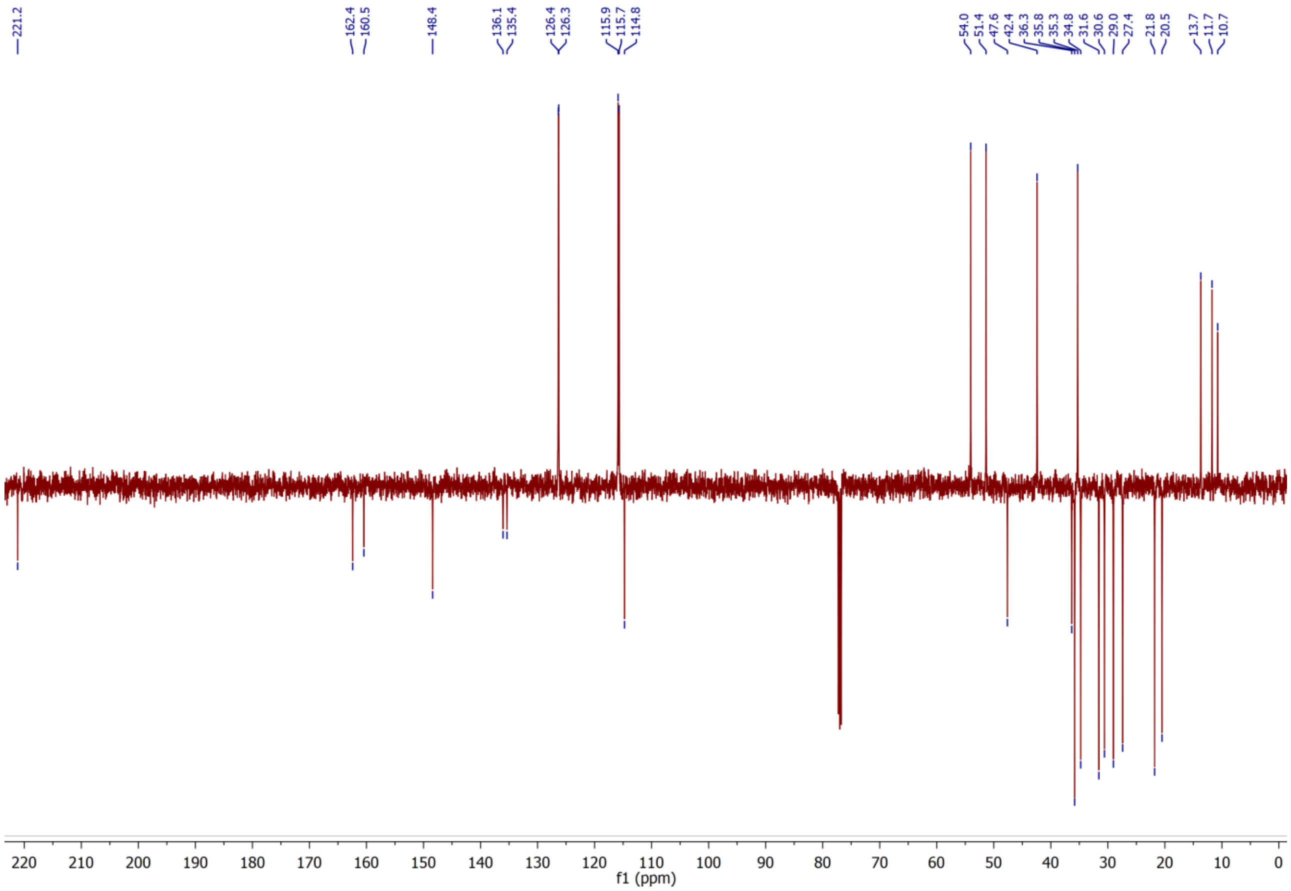
221.2
162.4
160.5
148.4
136.1
135.4
126.4
126.3
115.9
115.7
114.8
54.0
51.4
47.6
42.4
36.3
35.8
35.3
34.8
31.6
30.6
29.0
27.4
21.8
20.5
13.7
11.7
10.7
f1 (ppm)

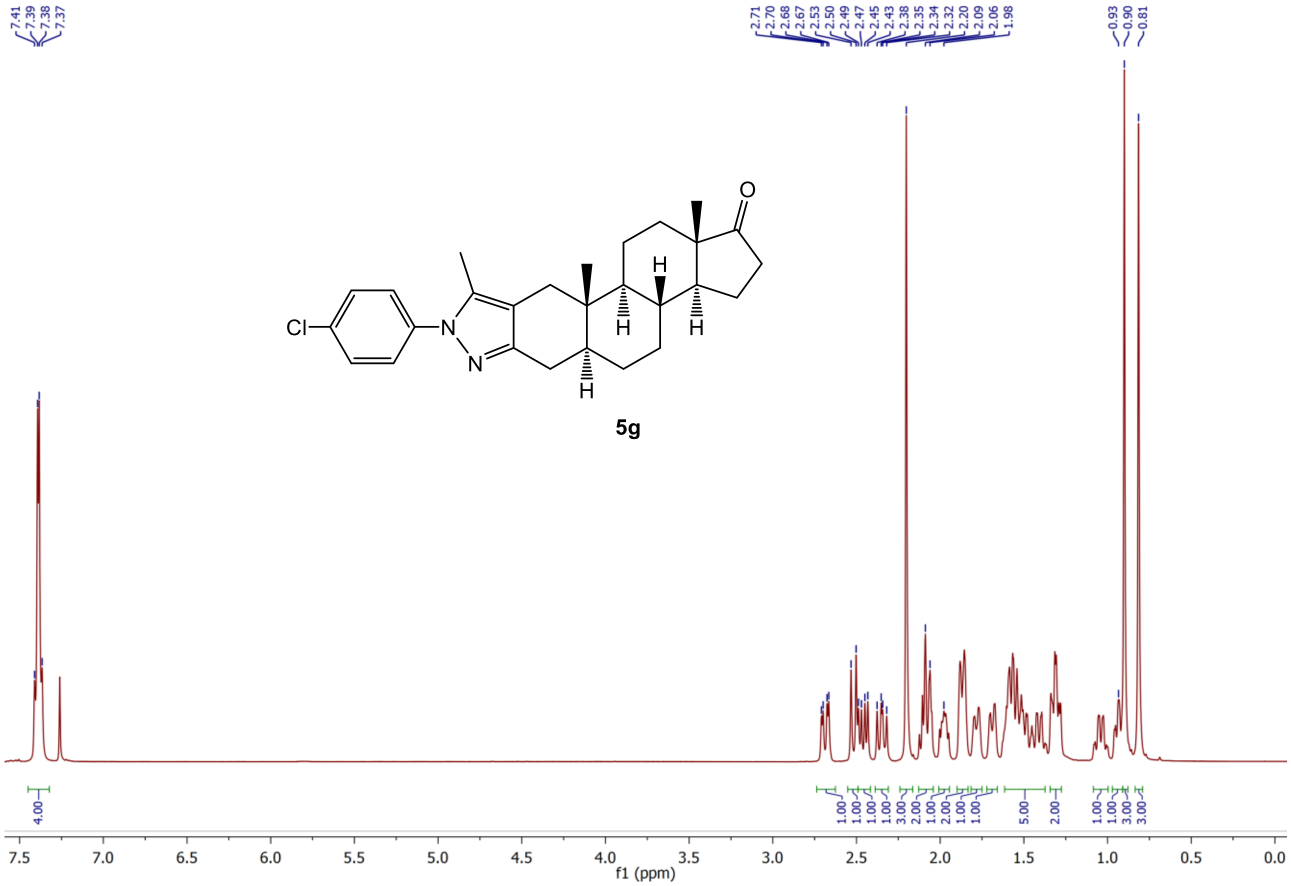
7.41
7.39
7.38
7.37
2.71
2.70
2.68
2.67
2.53
2.50
2.49
2.47
2.45
2.43
2.38
2.35
2.34
2.32
2.20
2.09
2.06
1.98
0.93
0.90
0.81
Cl
N
N
O
H
H
H
H
5g
4.00
1.00
1.00
1.00
1.00
3.00
2.00
1.00
2.00
1.00
1.00
5.00
2.00
1.00
1.00
3.00
3.00
f1 (ppm)

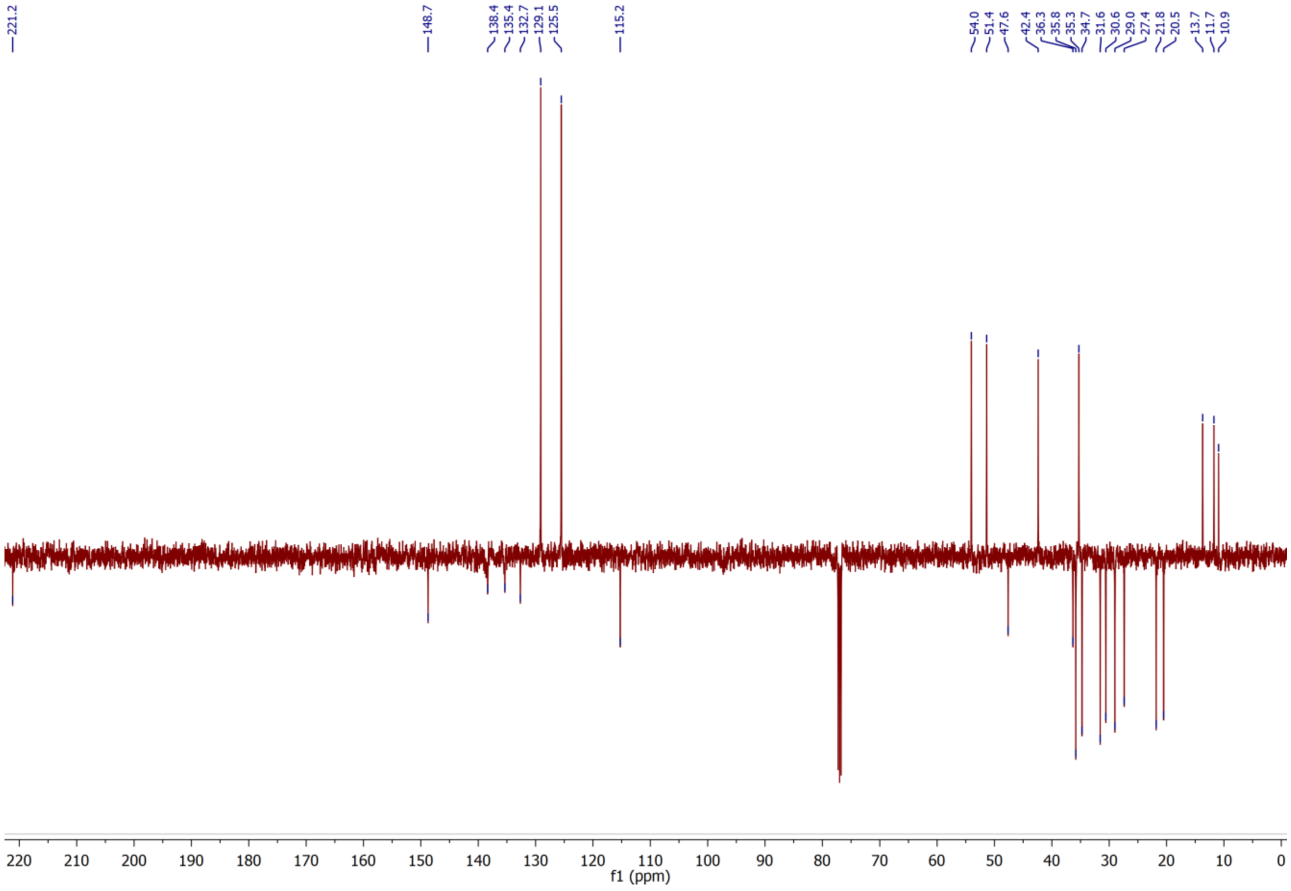
221.2
148.7
138.4
135.4
132.7
129.1
125.5
115.2
54.0
51.4
47.6
42.4
36.3
35.8
35.3
34.7
31.6
30.6
29.0
27.4
21.8
20.5
13.7
11.7
10.9
f1 (ppm)

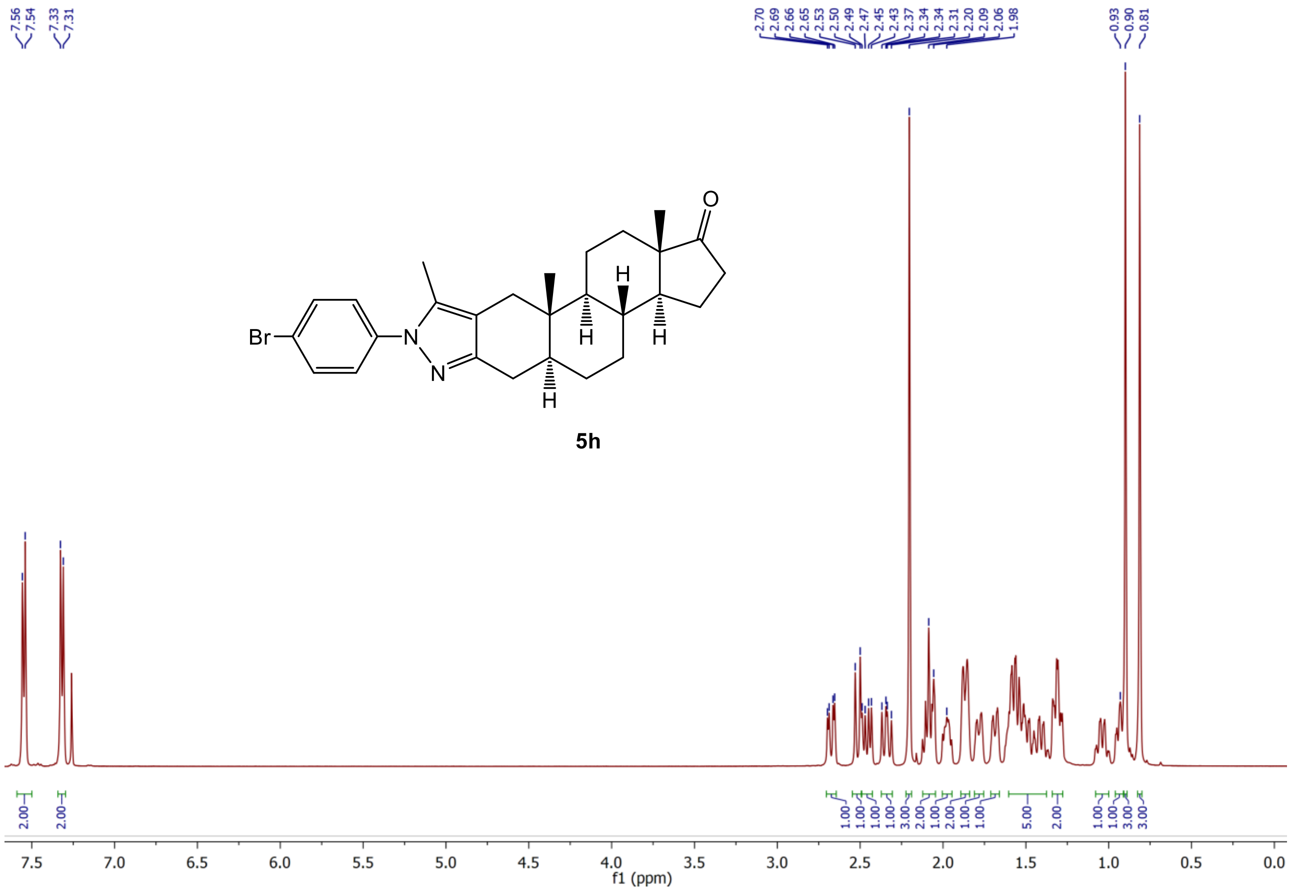
7.56
7.54
7.33
7.31
2.70
2.69
2.66
2.65
2.53
2.50
2.49
2.47
2.45
2.43
2.37
2.34
2.34
2.31
2.20
2.09
2.06
1.98
0.93
0.90
0.81
Br
N
N
O
H
H
H
H
5h
2.00
2.00
1.00
1.00
1.00
1.00
3.00
2.00
1.00
2.00
1.00
1.00
5.00
2.00
1.00
1.00
3.00
3.00
f1 (ppm)

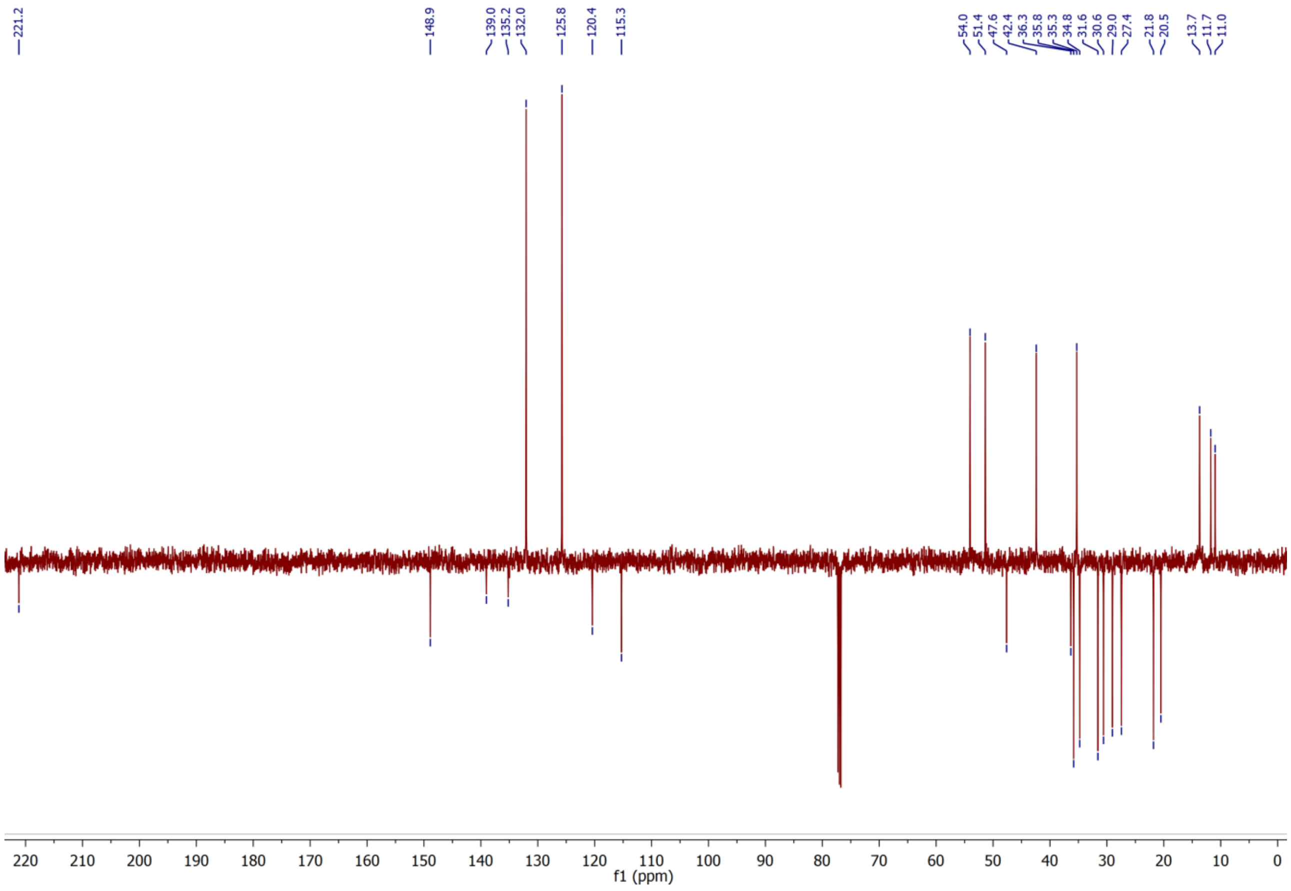
221.2
148.9
139.0
135.2
132.0
125.8
120.4
115.3
54.0
51.4
47.6
42.4
36.3
35.8
35.3
34.8
31.6
30.6
29.0
27.4
21.8
20.5
13.7
11.7
11.0
f1 (ppm)

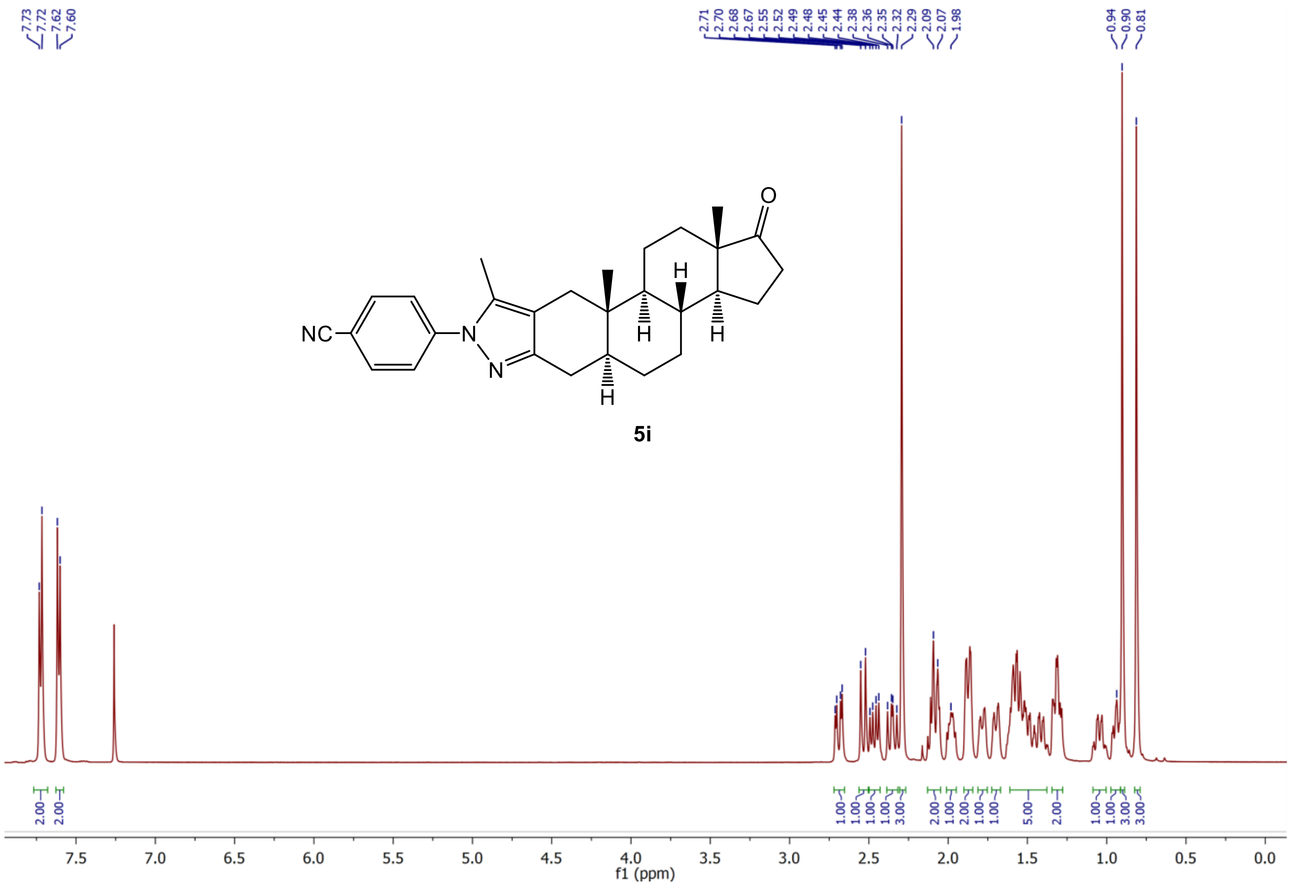
7.73
7.72
7.62
7.60
2.71
2.70
2.68
2.67
2.55
2.52
2.49
2.48
2.45
2.44
2.38
2.36
2.35
2.32
2.29
2.09
2.07
1.98
0.94
0.90
0.81
NC
N
N
O
H
H
H
H
5i
2.00
2.00
1.00
1.00
1.00
1.00
3.00
2.00
1.00
2.00
1.00
1.00
5.00
2.00
1.00
1.00
3.00
3.00
f1 (ppm)

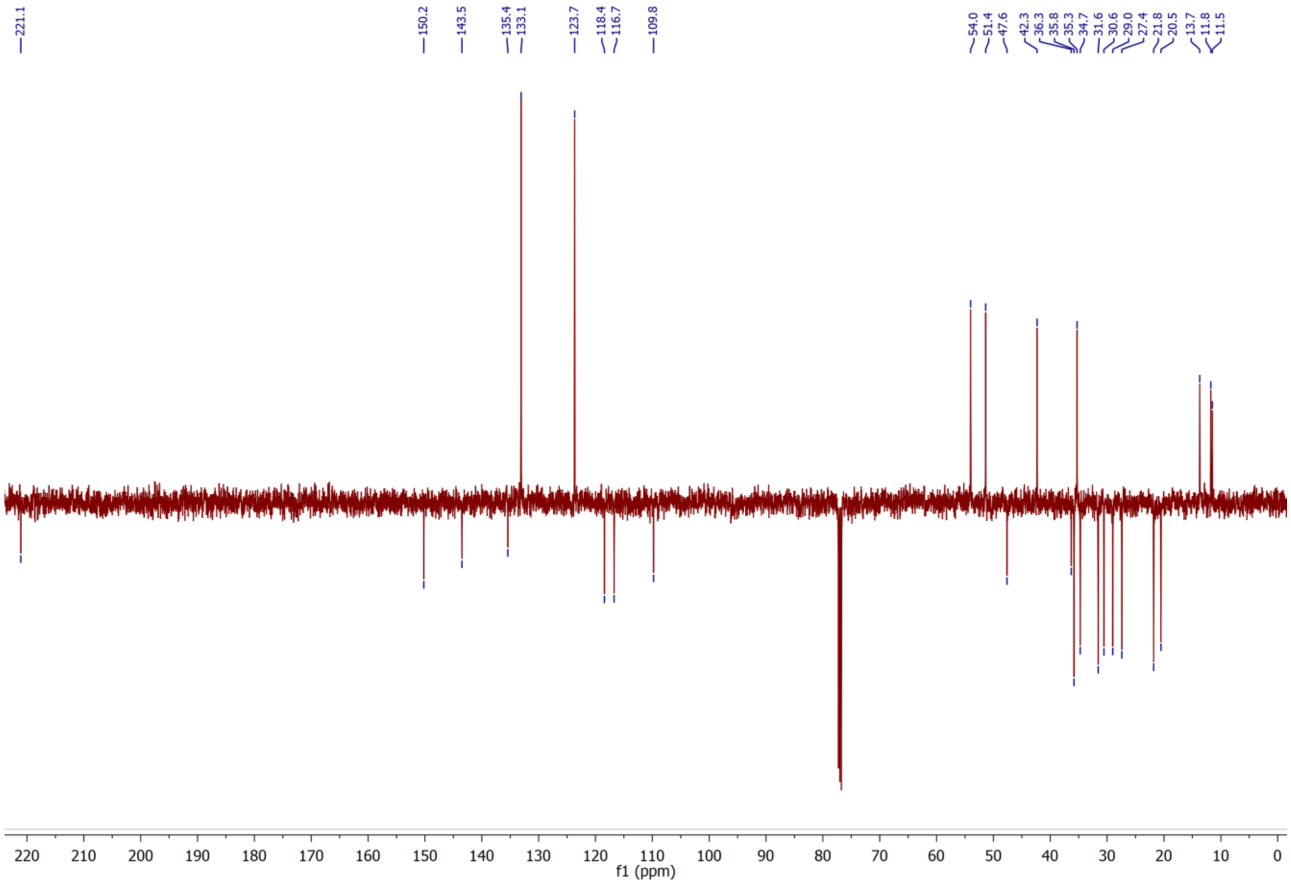
221.1
150.2
143.5
135.4
133.1
123.7
118.4
116.7
109.8
54.0
51.4
47.6
42.3
36.3
35.8
35.3
34.7
31.6
30.6
29.0
27.4
21.8
20.5
13.7
11.8
11.5
f1 (ppm)

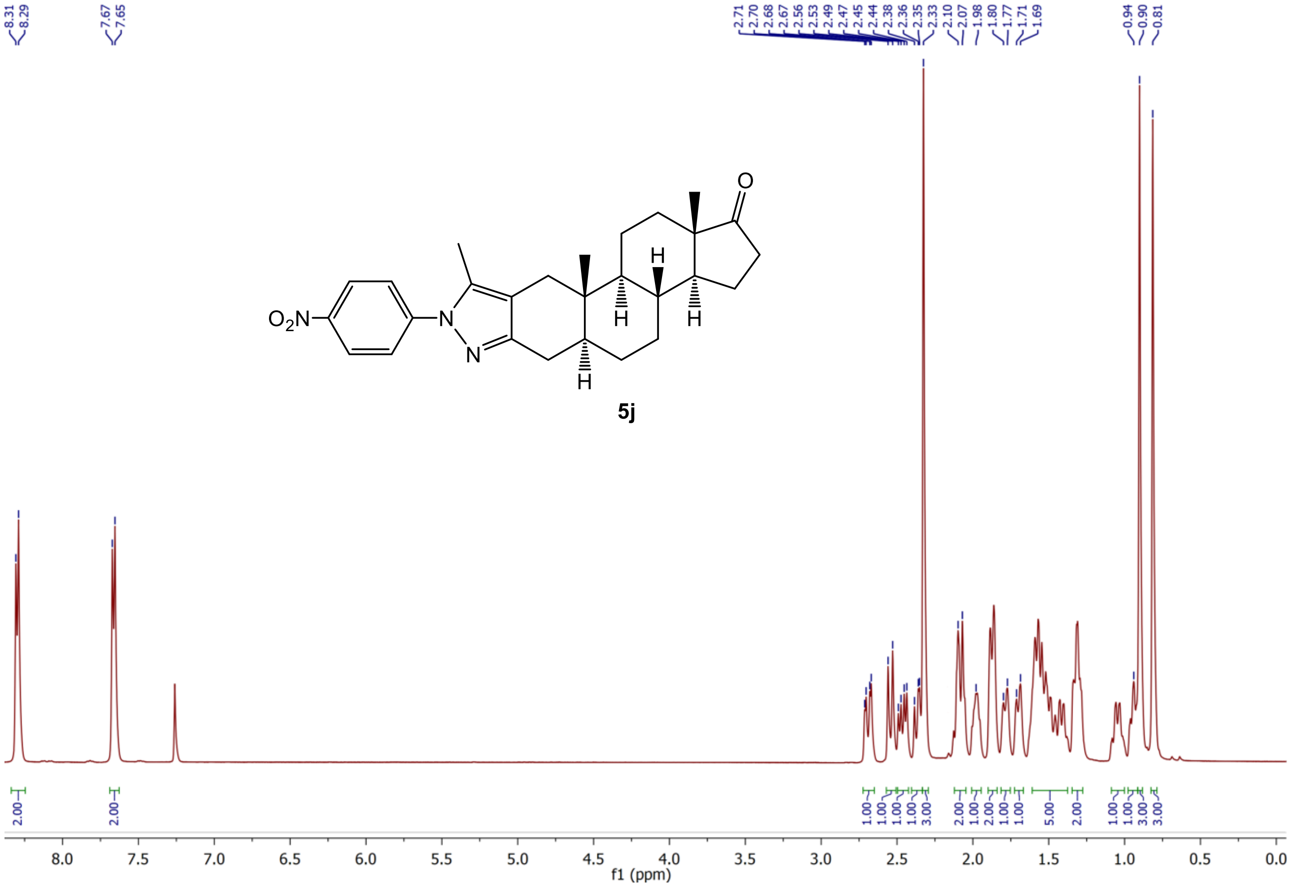
8.31
8.29
7.67
7.65
2.71
2.70
2.68
2.67
2.56
2.53
2.49
2.47
2.45
2.44
2.38
2.36
2.35
2.33
2.10
2.07
1.98
1.80
1.77
1.71
1.69
0.94
0.90
0.81
O
H
H
H
H
O_2N
N
N
5j
2.00
2.00
1.00
1.00
1.00
1.00
3.00
2.00
1.00
2.00
1.00
1.00
5.00
2.00
1.00
1.00
3.00
3.00
f1 (ppm)

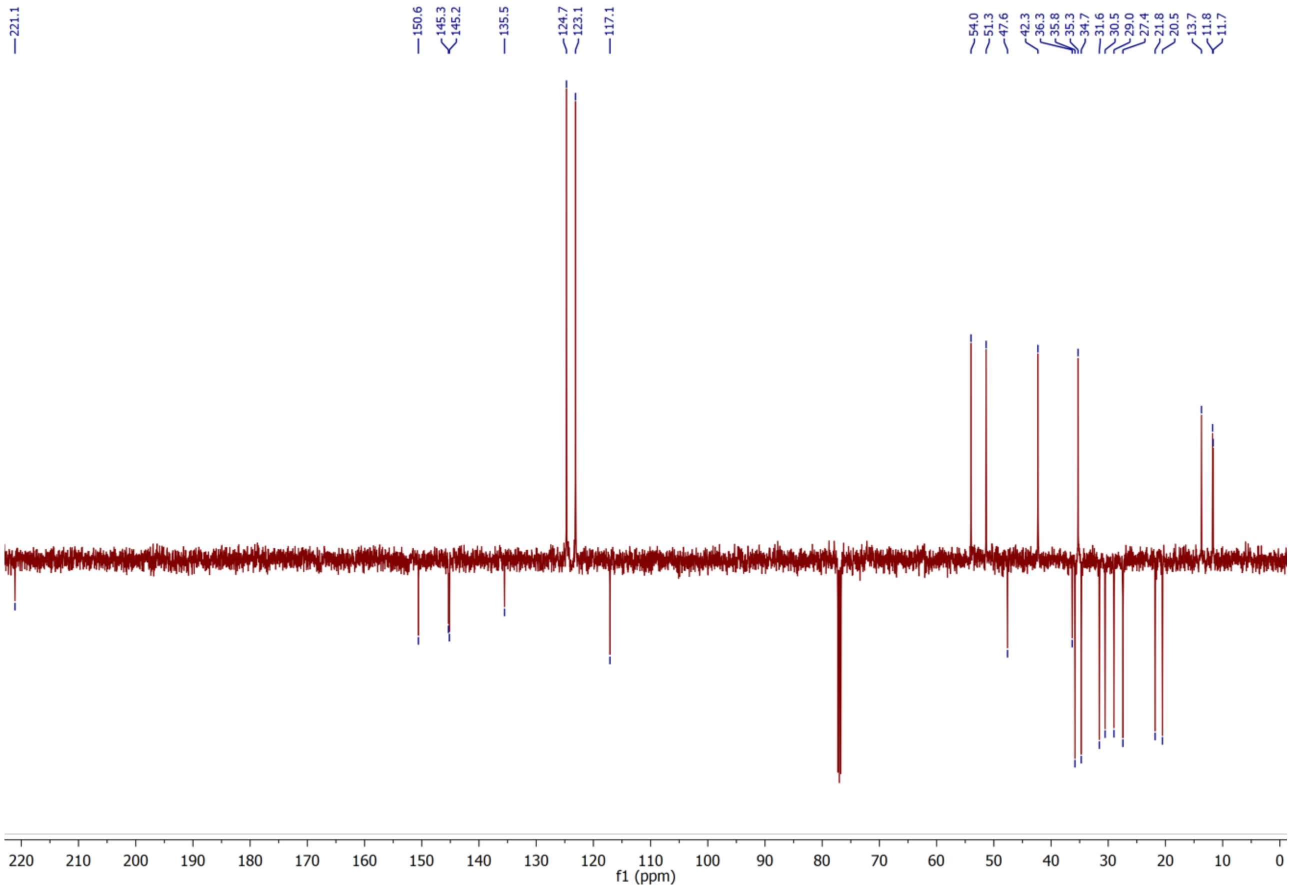
221.1
150.6
145.3
145.2
135.5
124.7
123.1
117.1
54.0
51.3
47.6
42.3
36.3
35.8
35.3
34.7
31.6
30.5
29.0
27.4
21.8
20.5
13.7
11.8
11.7
f1 (ppm)

Table S1. Mean values (%) of primary growth inhibitory screen used for heat map construction. Compounds were tested in 10 μM and 30 μM concentrations

	10 μM						30 μM					
	**MRC-5**	**DU 145**	**PC-3**	**HeLa**	**MCF-7**	**MDA-MB-231**	**MRC-5**	**DU 145**	**PC-3**	**HeLa**	**MCF-7**	**MDA-MB-231**
**4a**	68.3	67.3	36.8	49.6	57.1	75.0	1.2	1.5	3.2	46.7	5.5	32.1
**4b**	72.9	61.1	67.5	36.0	39.1	69.0	64.2	48.3	54.7	34.1	32.4	64.0
**5a**	78.0	80.1	47.7	70.5	73.3	85.0	3.9	4.0	7.6	26.2	7.5	15.2
**5b**	93.2	82.3	62.3	65.5	59.3	83.0	66.2	58.4	43.2	66.8	63.6	76.9
**4c**	75.0	87.3	44.0	56.1	49.5	81.7	37.5	5.1	4.2	8.0	5.4	12.0
**5c**	89.1	86.3	68.1	46.3	63.6	78.7	62.5	51.1	43.6	38.5	48.7	66.2
**4d**	78.9	70.2	53.4	65.6	74.5	92.0	0.8	2.3	3.6	56.1	10.5	47.9
**5d**	80.1	65.6	52.1	39.5	56.3	77.2	21.3	10.0	14.6	30.7	13.3	54.1
**4e**	71.2	23.3	28.4	31.7	20.9	35.2	28.8	5.5	10.8	15.1	14.0	13.6
**5e**	79.3	62.1	45.5	38.4	53.5	72.0	79.3	48.1	29.7	41.8	36.3	61.5
**4f**	68.7	71.6	41.8	46.1	57.1	83.4	14.8	3.9	5.1	7.6	5.4	32.0
**5f**	83.9	74.9	54.3	40.5	72.6	82.3	66.1	50.7	17.6	50.5	30.6	66.0
**4g**	80.1	74.8	44.6	40.1	48.5	78.4	1.1	1.4	3.0	4.7	5.3	5.3
**5g**	94.4	73.7	54.7	38.7	56.3	65.7	18.1	23.2	11.8	39.0	28.0	65.1
**4h**	92.8	95.1	61.6	38.2	54.1	69.4	12.7	2.2	3.6	5.8	5.2	10.5
**4i**	100.3	104.9	56.6	62.9	53.6	93.0	97.4	105.7	53.3	72.5	50.4	89.1
**5i**	90.4	83.3	56.2	42.2	53.2	70.6	87.9	79.9	70.0	36.4	39.4	59.7
**4j**	99.1	82.8	38.2	60.5	55.8	83.8	99.8	60.4	41.6	64.6	49.6	80.6
**5j**	94.8	73.0	36.8	47.4	46.5	83.5	44.6	42.8	16.0	29.4	14.4	71.7

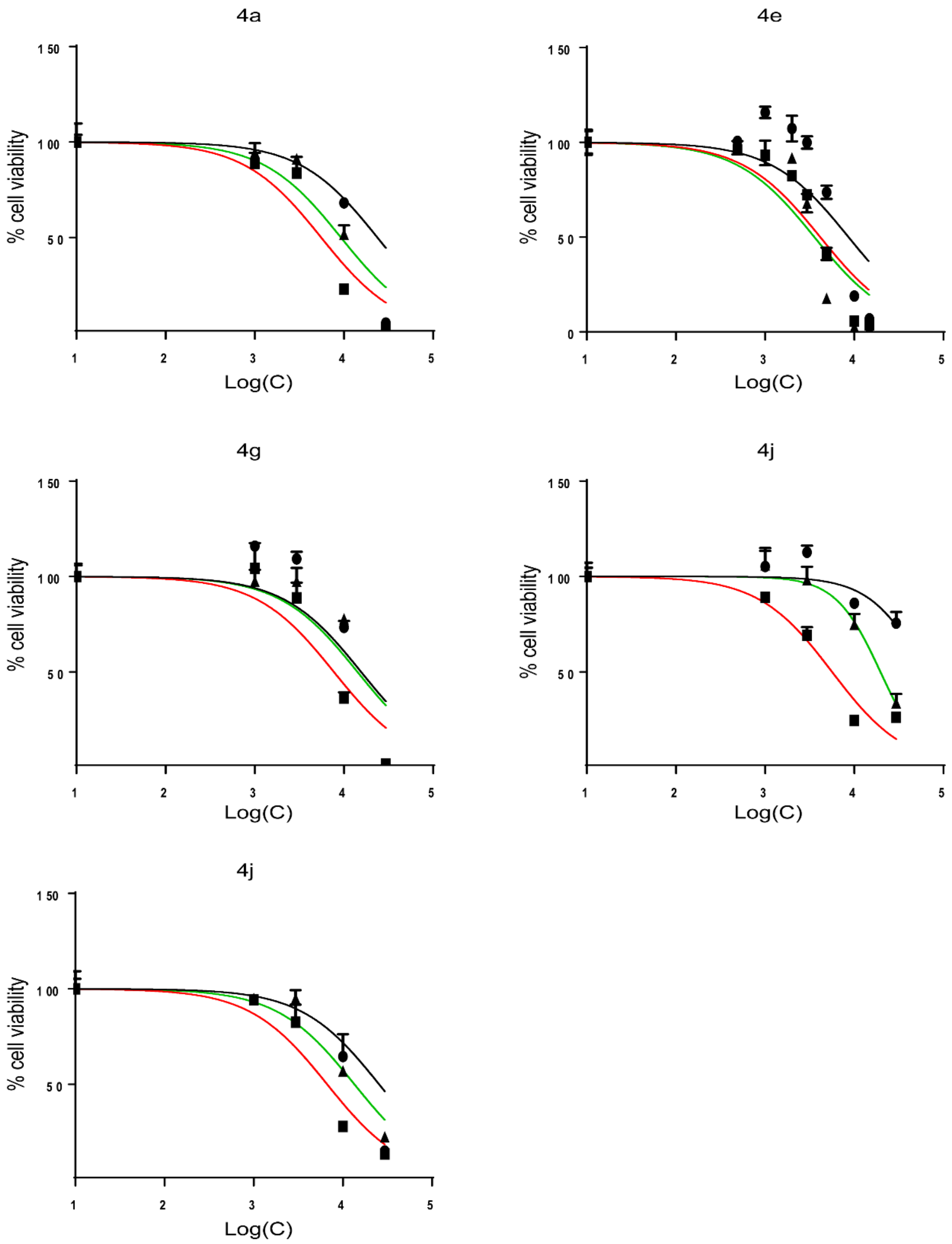

Figure S1a: Representative cell viability curves to determine growth inhibition and IC_{50} values following treatments with compounds **4a, 4e, 4g, 4j** and **5j** on MRC-5 (black), PC-3 (red) and DU 145 (green) cells. Log (C) values were calculated from nM concentrations. Based on the viability data, IC_{50} values (expressed in µM concentration in Table 2.) were determined.

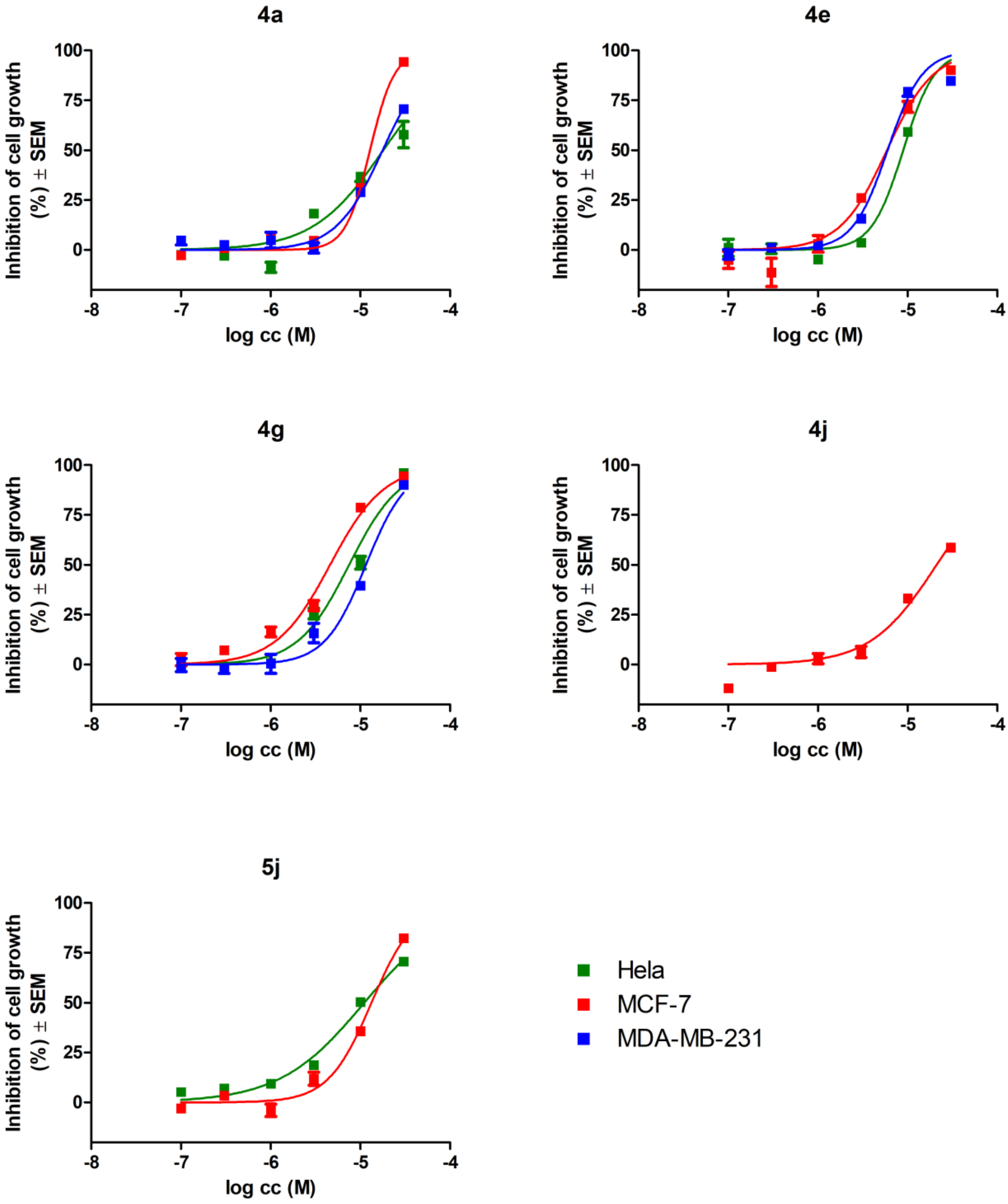

Figure S1b: Representative growth inhibition curves following treatments with compounds **4a, 4e, 4g, 4j** and **5j** on HeLa (green), MCF-7 (red) and MDA-MB-231 (blue) cells. Based on these data, IC_{50} values (expressed in µM concentration in Table 2.) were determined.
